# Development and Mechanistic Studies of Oxidative Prins-Semipinacol Cyclization Reactions

**DOI:** 10.1021/acs.orglett.6c00814

**Published:** 2026-04-13

**Authors:** Max O. Kogut, Karen R. Garn, Michael J. Kerner, Paul E. Floreancig

**Affiliations:** Department of Chemistry, 6614 University of Pittsburgh, Pittsburgh, Pennsylvania 15260, United States

## Abstract

This work shows that allylic alcohols or silyl ethers promote hydride abstraction leading to direct carbon–carbon bond formation or carbon–oxygen bond formation followed by rearrangement, providing the stereoselective construction of spirocyclic ethers through a Prins-semipinacol pathway. A kinetic isotope effect study showed that hydride abstraction is the rate-determining step, consistent with the presence of a distal nucleophilic group lowering the transition state free energy of the oxidation step.

## Graphical abstract

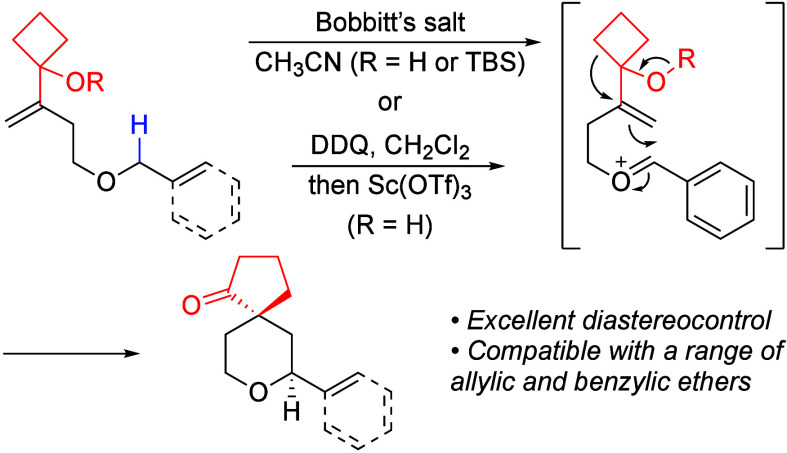

Unique chemoselectivity, mild reaction conditions, readily prepared substrates, and access to cationic intermediates make carbon–hydrogen bond cleavage by organic oxidants highly effective transformations in complex molecule synthesis.^1^ We^2^ and others^3^ have conducted extensive computational and experimental studies in an effort to understand the mechanisms of hydride abstractions by quinone and oxoammonium ion oxidants to gain insights into oxidative entries to oxocarbenium, acyliminium, and thiocarbenium ions. This work has provided a sound foundation for qualitative predictions of kinetic and thermodynamic trends in reactions with a wide variety of substrates. It does not, however, explain our observation that hydride abstraction rates are strongly influenced by the presence of a competent distal nucleophile in all but the most thermodynamically favorable oxidation reactions.^4^ This is illustrated by the differential reactivity shown in Scheme 1, in which enol acetate **1** smoothly forms tetrahydropyrone **2** upon treatment with 2,3-dichloro-4,5-dicyano-1,4-benzoquinone (DDQ)^2c^ while unfunctionalized alkene **3** requires the addition of SnBr_4_ to form tetrahydropyran **4**.^5^ These results led us to hypothesize that the energetic benefit of carbonyl formation expedites the reaction though the origin of this effect remained unclear.

**Scheme 1:**
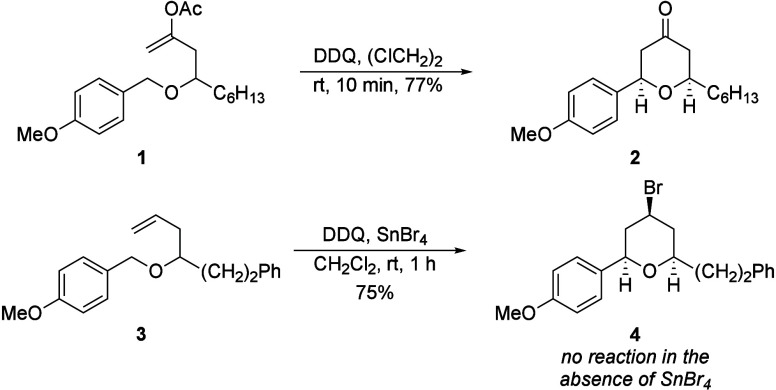
Influence of the Nucleophile in Oxidative Cyclization Reactions

Our postulate of carbonyl group generation driving the enhanced oxidation rates of enol acetate substrates relative to unfunctionalized alkenes led us to design new alkene-based nucleophiles with sufficient reactivity to promote oxidative cyclization reactions. Overman’s studies^6^ on the use of allylic alcohols as nucleophiles to promote Lewis acid-mediated Prins-semipinacol rearrangements^7^ inspired the study described herein, as seen in the generalized conversion of **5** to cation **6** followed by rearrangement to **7** (Scheme 2). This manuscript illustrates the successful realization of the oxidative Prins-semipinacol cyclization to form spirocyclic products. Two variants are reported, with one using *N*-2,2,6,6-tetramethyl-1-oxopiperidin-1-ium-4-yl)­acetamide tetrafluoroborate (Bobbitt’s salt) to initiate the reaction directly from alcohol or silyl ether substrates and the other proceeding stepwise from alcohol substrates with 2,3-dichloro-5,6-dicyano-1,4-benzoquinone (DDQ) to form acetal intermediates followed by Lewis acid-mediated ionization and rearrangement. We also show that the migration mechanism is influenced by the presence or absence of strain release in the transition state. These studies furthermore provide numerous insights into nuances of the oxidation mechanism.

**Scheme 2:**
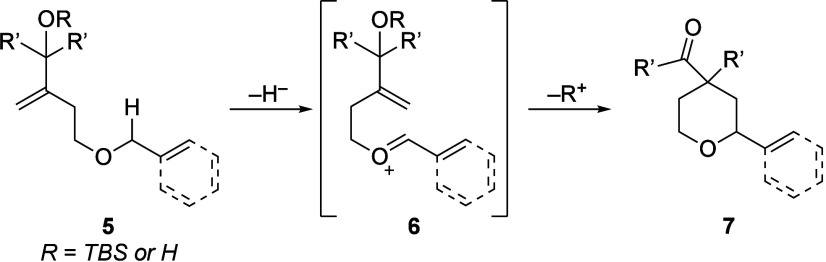
General Oxidative Prins-Semipinacol Cyclization Reaction

Vinyl cyclobutanols show enhanced nucleophilicity compared to unsubstituted alkenes in nucleophilic addition and migration sequences,^8^ leading us to incorporate this group into our initial substrate design. The substrate synthesis proceeded through the straightforward path shown in Scheme 3. Oxidizing **10** with Bobbitt’s salt (**11**) rapidly resulted in the formation of **12** as a single diastereomer in 74% yield, with a similar 71% yield on 1 mmol scale. The stereochemical outcome is consistent with a pathway that proceeds through **13**, whereby the semipinacol ring expansion ensues in a concerted fashion with tetrahydropyran formation via a net *anti*-addition across the alkene. This outcome is consistent with prior Prins-semipinacol studies that result in ring expansion.^9^

**Scheme 3:**
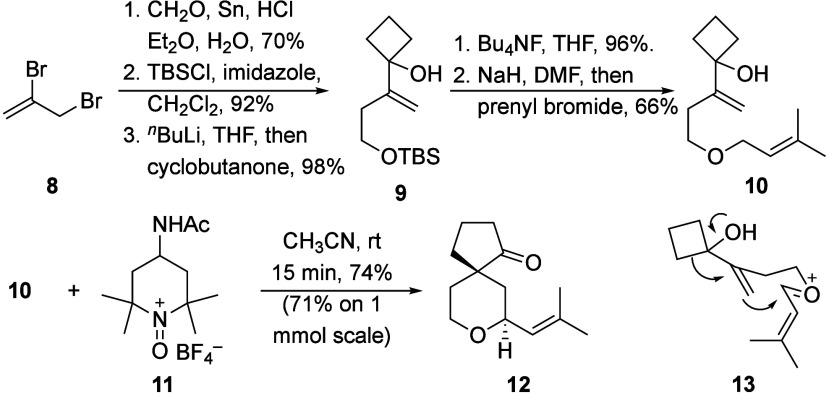
Substrate Synthesis and Initial Demonstration of the Oxidative Prins-Semipinacol Reaction

This reaction showed the viability of this transformation, in which a highly reactive prenyl ether undergoes hydride abstraction, but challenges in scope expansion arose for ethers that show lower hydricity. Oxidative transposition of the vinyl cyclobutanol group^10^ becomes a notable side reaction when ether oxidation is slow, as seen in the reaction of allyl ether **14** to form an approximately 1:1 ratio **15** and **16** (Scheme 4) at less than 20% conversion over 48 h. This led us to explore alternate oxidation conditions. DDQ is a substantially weaker oxidant than Bobbitt’s salt but is highly effective at converting vinyl ethers to oxocarbenium ions.^2a^ Vinyl ether **17** was prepared through McDonald’s conditions.^11^ We employed DDQ as the oxidant since we^2b^ and others^12^ previously observed that Bobbitt’s salt reacts as an electrophile toward electron rich alkenes. This resulted in rapid starting material consumption, but the observed product was acetal **18**, formed as a 3:1 mixture of *E*- and *Z*-isomers, rather than the expected spirocyclic ether. We realized that the relevant oxocarbenium ion for the Prins-semipinacol rearrangement could be regenerated through a Lewis acid-mediated acetal ionization reaction. After screening several common Lewis acids we observed that Sc­(OTf)_3_ converted **18** to **19** within 40 min, in which **19** was isolated as a 4:1 mixture of alkene isomers in 65% yield.

**Scheme 4:**
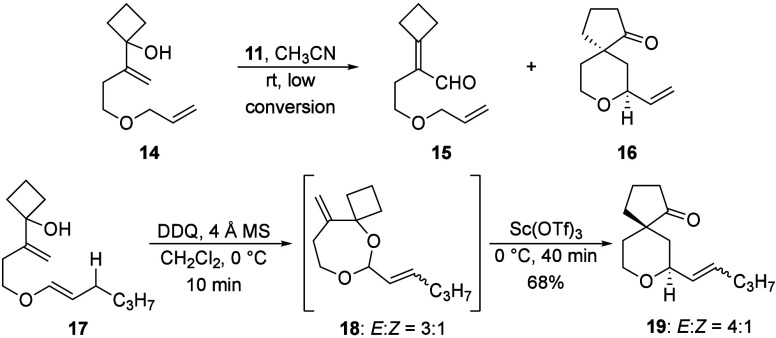
Competitive Rearrangement with Slowly Oxidized Substrates and the Incorporation of DDQ as an Oxidant

The one-pot oxidative acetal formation and rearrangement sequence inspired us to explore allylic and benzylic, rather than alkenyl, ethers as substrates. While this lowers the oxidation rates, it allows for alkene geometry control and for substrate synthesis through facile Williamson etherification conditions. Liu showed^13^ enhanced rates when alcohols or water are used as nucleophiles in hydride abstraction reactions in comparison to alkenes. This can be exploited to compensate for the inherently slower reactions of allylic and benzylic ethers. We tested the viability of this hypothesis as shown in Scheme 5. Exposing ether **20** to DDQ resulted in the formation of acetal **18** after 20 h and adding Sc­(OTf)_3_ provided **19** but as a single stereoisomer in 67% yield (72% based on recovered starting material) without losing alkene geometric integrity.

**Scheme 5:**
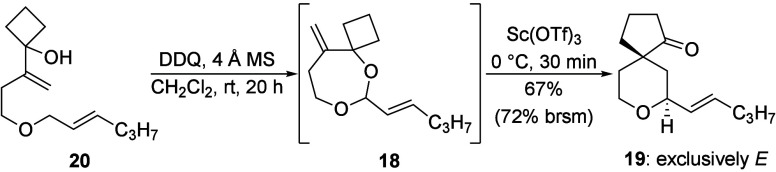
Two-Step Oxidative Rearrangement from an Allylic Ether

The success of this transformation led us to expand the scope of the process (Table 1). The substrates for this study were selected based on our prior observations that unsaturation is required for oxidation and that electron-withdrawing groups on arenes suppress reactivity.^2c^ Under these conditions prenyl ether **10** reacted to form **12** in 68% yield (entry 1), which is similar to the single step protocol with Bobbitt’s salt. Cyclic trisubstituted alkenes react efficiently, as seen in the conversion of **21** to **22**. Allyl ether **14** still proved to be challenging, as expected based in the minimally stabilized cationic intermediate, resulting in a 29% yield of **16** at 60% conversion (entry 3). Benzyl ether **23** required gentle heating to promote the oxidation step but rearranged efficiently to provide **24** in 76% yield (entry 4). 3,5-Dimethoxybenzyl ether **25** oxidized at rt, resulting in the formation of **26** in 80% yield (entry 5). Naphthylmethyl ether **27** was an excellent substrate, leading to the formation of **28** in 93% yield (91% on 1 mmol scale, entry 6). Electron-rich benzylic ether **29** oxidized rapidly and rearranged to form **30** in 63% yield (entry 7). Replacing the cyclobutanol nucleophile with a Boc-protected azetidinol (**31**) resulted in a less efficient transformation but provided the intriguing heterocycle **32** in a usable yield (entry 8). Prolonged exposure of products **22** and **32** to Sc­(OTf)_3_ erodes stereocontrol through Lewis acid-mediated ionization (**22**) or a retro-Mannich/Mannich sequence (**32**).

**Table 1:**
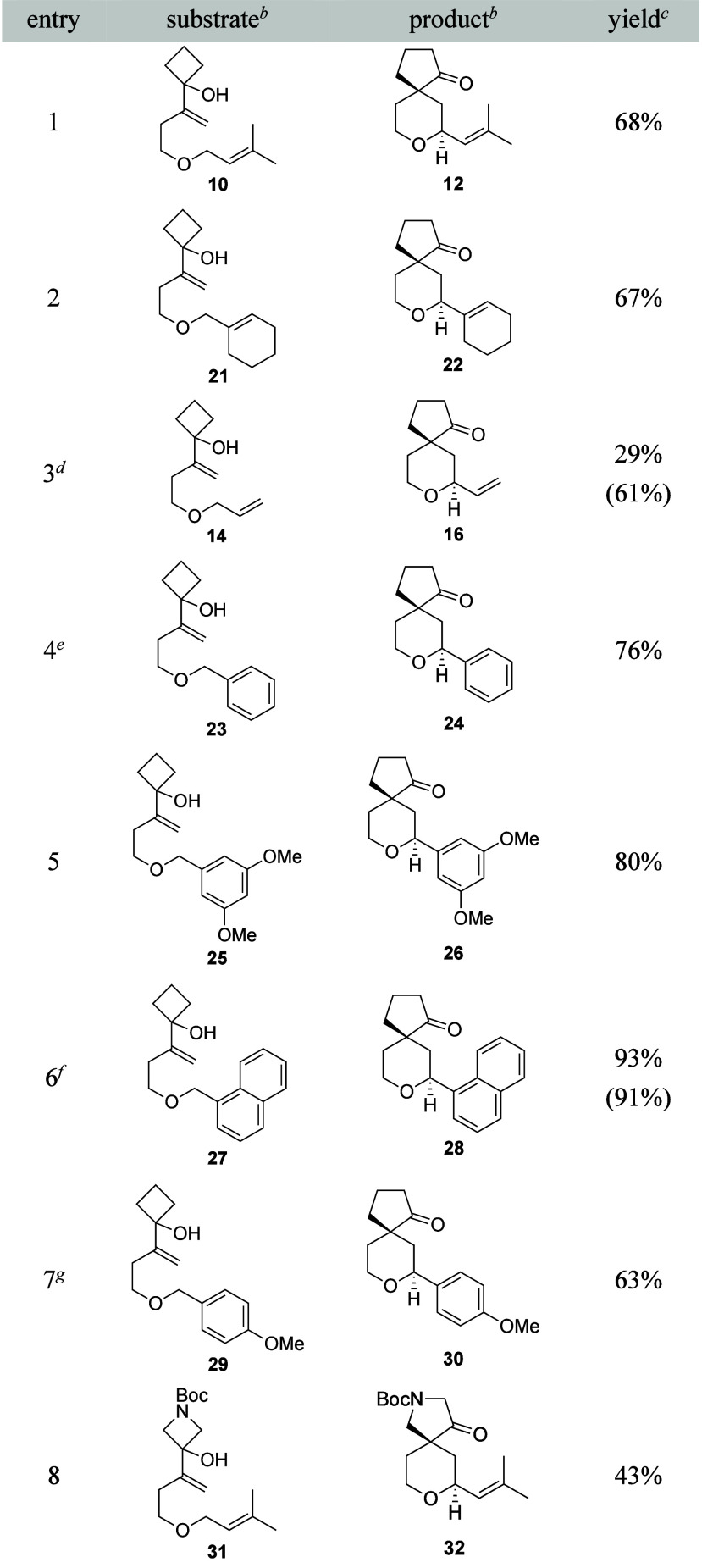
Scope Expansion^a^

a Reaction conditions: Substrate (1 equiv), DDQ (1.5 equiv), and 4 Å MS (1 mass equiv) were stirred in CH_2_Cl_2_ at rt for approximately 20 h. Upon completion of the oxidation the mixture was cooled to 0 °C and Sc­(OTf)_3_ (10 mol %) was added and the reactions were stirred for 10 to 30 min unless otherwise noted. See the for details of individual reactions.

b See the for the syntheses of the substrates characterization data for all substrates and products.

c Yields are for isolated, purified material.

d Parenthetical yield is based on recovered starting material.

e Oxidation required heating to 40 °C.

f Parenthetical yield is on 1 mmol scale.

g Oxidation proceeded to completion within 1 h.

We prepared compound **33** to determine whether a stereocenter in the substrate can dictate the generation of additional stereocenters. Exposure to DDQ (Scheme 6) followed by a subsequent treatment with Sc­(OTf)_3_ proceeded through the expected transition state **34** ^2b–2d^ to deliver **35** as a single diastereomer in 71% yield. Notably, the Sc­(OTf)_3_-mediated rearrangement required 2 h to proceed to completion, in contrast to the shorter reaction times observed for other substrates. The branching in the initially formed acetal most likely inhibits ring opening and rearrangement to intermediate **34**. This route offers opportunities to prepare the spirocyclic products in enantiomerically enriched form through incorporating enantioselective aldehyde bromoallylation reactions^14^ for substrate syntheses.

**Scheme 6:**
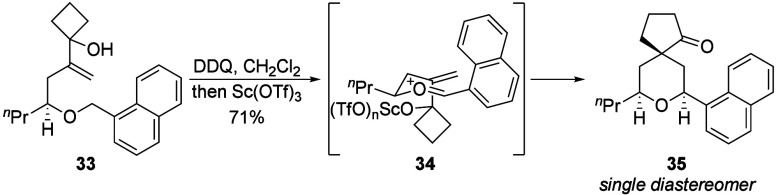
Diastereoselective Reaction from a Chiral Substrate

Nonstrained substrates showed a different reactivity pattern (Scheme 7). Tertiary alcohol **36** proceeded through oxidative acetal formation to form **37**, but this substrate proved to be inert toward Sc­(OTf)_3_-mediated ionization and was isolated in 33% yield. We postulated that ionization was occurring but that the Prins reaction was inhibited in the absence of accompanying strain release. We addressed this problem by forming a silyl ether of the substrate (**38**) to suppress acetal formation. DDQ proved to be an insufficient oxidant to promote oxidative carbon–hydrogen bond cleavage from **38**, but oxidation with Bobbitt’s salt (**11**) formed **39** in 20 h without the formation of the acetal intermediate. This reaction proceeded in good chemical yield but, in contrast to previous reactions, showed no stereocontrol. This suggests that, while strain release promotes a pathway in which the semipinacol and Prins reactions are concerted or rapidly successive, the absence of strain release extends the lifetime of cation **40**, allowing for methyl group migration from either face. We observed similar results in preliminary studies (not shown) of cyclopentanol-containing studies that were not pursued further.

**Scheme 7:**
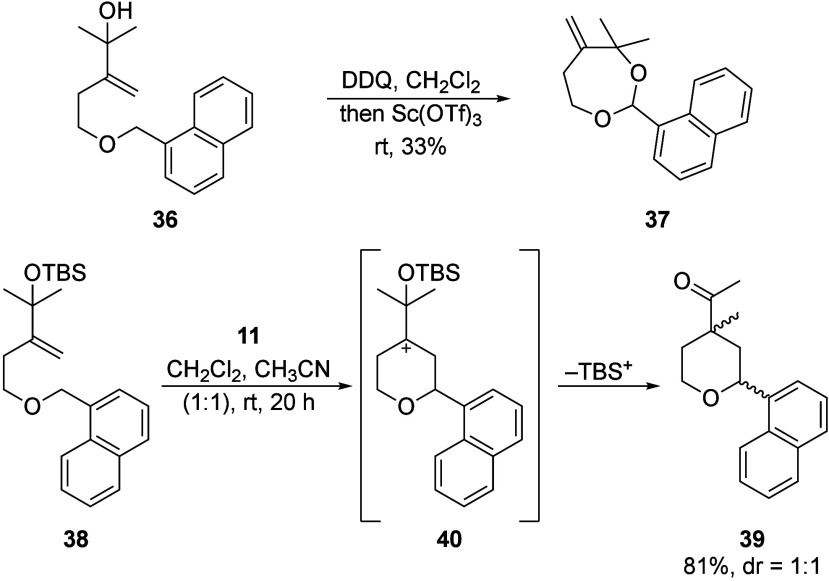
Cyclization and Rearrangement without Strain Release

These results provide useful mechanistic insights into oxidative carbon–hydrogen bond cleavage (Scheme 8). Starting material consumption without acetal formation was not observed unless cyclization was extremely slow. This observation can be explained by cyclization, rather than oxidation, being the rate-determining step in these reactions, or by the oxidation rate being influenced by the presence of a pendent nucleophile. We tested these options by preparing monodeuterated substrate **25-D**_**1**_ and subjecting it to the oxidation conditions to form acetal **40** (Scheme 8). This transformation showed a large primary kinetic isotope effect of 8.1 as determined by ^1^H NMR, indicating that C–H cleavage is the rate-determining step in the oxidative cyclization. This large value matches our previous studies on the DDQ-mediated oxidation of 3,5-dimethoxybenzyl ethers,^15^ and is consistent with reenforcing primary and secondary kinetic isotope effects. When coupled with prior observations of enol acetate nucleophiles enhancing oxidation rates, this strongly suggests that electrostatic interactions with distal nucleophiles can stabilize the transition state for oxidative C–H cleavage. Transition state stabilization in oxocarbenium ion formation is known in acetal hydrolysis reactions^16^ but, to the best of our knowledge, has not been proposed in hydride abstractions. The absence of an acetal intermediate in reactions with Bobbitt’s salt most likely results from the use of the more polar CH_3_CN (required for regent solubility), in contrast to CH_2_Cl_2_, thereby lowering the barrier for acetal ionization and rearrangement.

**Scheme 8:**
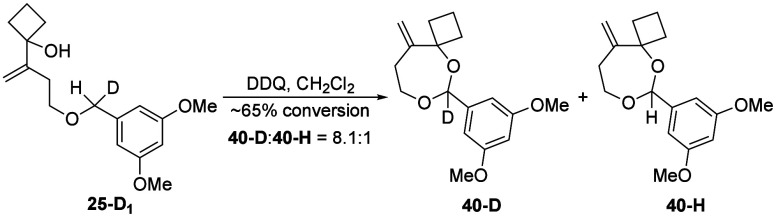
Intramolecular Kinetic Isotope Effect Study

The products of these reactions are can be converted to additional ring systems, with representative examples shown in Scheme 9. Exposing **28** to *m*-CPBA provided lactone **41**. Conversion of **28** to an oxime followed by treatment with catalytic Re_2_O_7_ ^17^ promoted a Beckmann rearrangement to yield **42**. These transformations illustrate the potential for diversifying the products to access alternate spirocycles, which are compounds whose significance is growing in medicinal chemistry.^18^

**Scheme 9:**
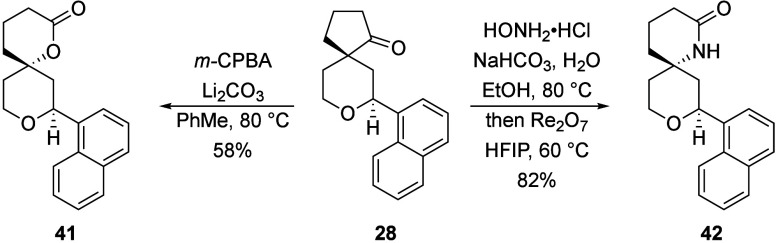
Product Diversification

We have shown that the alkene groups in allylic alcohols can engage in Prins-type cyclization reactions with oxidatively generated oxocarbenium ions whereby the addition is followed by a rapid bond migration resulting in thermodynamically favorable ketone formation. The scope of this Prins-semipinacol reaction can be expanded by switching the hydride abstracting agent from Bobbitt’s salt to DDQ. Vinyl ethers react rapidly to form cyclic acetal intermediates that can be converted to the Prins-semipinacol product by reionization with Sc­(OTf)_3_. Allylic and benzylic ethers react more slowly and require the presence of a hydroxy group. The high stereocontrol in the products suggests that the Prins and semipinacol steps are either concerted or very rapidly consecutive with the two carbon–carbon bonds being formed through a net *anti*-addition across the alkenes. Conducting the reaction without the potential for strain release eliminates the stereocontrol, consistent with a longer-lived intermediate cation from the Prins step. The outcomes of these reactions provided mechanistic insights into oxidative carbocation formation that will be useful in guiding future efforts in this field. Previous studies in which acyliminium ions can be formed from the oxidation of allylic amides and related species by Bobbitt’s salt^2f^ and from vinyl amides with DDQ^19^ strongly suggest that this work can be extended to form nitrogen-containing heterocycles, and this is currently under investigation.

## Supplementary Material





## Data Availability

The data underlying this study are available in the published article and its .

## Notes

Experimental procedures, characterization data (PDF)
FAIR data, including the primary NMR FID files, for compounds **9**, **10**, **12**, **14**, **16**, **17**, **19**–**33**, **35**–**39**, **41**, **42**, **19′**, **25-D_1_**, **39′**, **40-D**, **S1**–**S4** (ZIP)
