# Supplementary material for: Development and Mechanistic Studies of Oxidative Prins-Semipinacol Cyclization Reactions
Source: Org Lett. 2026 Apr 13;28(16):5092–6. doi: 10.1021/acs.orglett.6c00814 (PMC13123410; doi:10.1021/acs.orglett.6c00814)
Supplement: Supplementary file 1 [file ol6c00814_si_001.pdf]

Supporting Information

Development and Mechanistic Studies of Oxidative Prins-Semipinacol Cyclization Reactions

Max O. Kogut, Karen R. Garn, Michael J. Kerner, and Paul E. Floreancig*
Department of Chemistry
University of Pittsburgh
Pittsburgh, Pennsylvania 15260

florean@pitt.edu

Table of Contents

General Experimental S3

Experimental Procedures and Characterization S4

^{1}H and ^{13}C NMR Spectra S19

References S58

General Procedure

Unless otherwise indicated all reactions were performed in flame-dried glassware under an inert atmosphere of dry argon and stirred with Teflon-coated magnetic stir bars. All commercially available compounds were purchased and used as received unless otherwise specified. Dichloromethane (CH_2Cl_2) and acetonitrile (MeCN) were distilled from calcium hydride under N_2 prior to use. Tetrahydrofuran (THF) was distilled from sodium benzophenone ketyl under N_2 prior to use. Ar and N_2 gasses were purchased from Matheson Tri Gas. Analytical TLC was performed on E. Merck pre-coated (25 mm) silica gel 60 F254 plates. Visualization was done under UV (254 nm) and by staining with anisaldehyde or potassium permanganate stain. Purification of compounds by flash chromatography were performed using silica gel (SiliCycle SiliaFlash P60 40-60 63μm 60 Å silica gel). Reagent grade ethyl acetate, diethyl ether, dichloromethane, methanol, and hexanes (commercial mixture) were purchased from Fisher Scientific and were used as-received for chromatography. ^1H and ^{13}C NMR spectra were recorded on Bruker Avance 300, 400, or 500 MHz spectrometers. Spectra were referenced to residual chloroform (7.26 ppm, ^1H; 77.16 ppm ^{13}C). Chemical shifts (δ) are reported in ppm and multiplicities are indicated by s (singlet), d (doublet), t (triplet), q (quartet), and m (multiplet). Coupling constants, *J*, are reported in hertz (Hz). All NMR spectra were obtained at room temperature. Infrared (IR) spectra were taken on a Nicolet IR200 FT-IR spectrometer with an ATR attachment.

General Procedure A: DDQ-mediated oxidative cyclization.

DDQ (1.5 equiv) and 4 Å molecular sieves (1 mass equiv relative to the substrate) were added to a solution of substrate (1 equiv) in CH_2Cl_2 (0.1 M). Upon starting material consumption, as determined by TLC, the mixture was cooled to 0 °C and $Sc(OTf)_3$ (0.1 equiv) was added. The reaction stirred until acetal was fully consumed (10 min to 1 h). The reaction was filtered through cotton and concentrated. The crude mixture was purified by flash chromatography to provide the desired product.

General Procedure B: Ether preparation.

Diol **S3** (1 equiv) was added dropwise to a solution of NaH (60% oil dispersion, 1.2 equiv) in DMF (0.2 M) at 0 °C (*CAUTION* NaH is a strong base that reacts violently with water and other acids. Handle this agent under an inert atmosphere). The mixture was stirred for 20 min, then the appropriate allylic or benzylic halide (1.1 equiv) was added dropwise. Upon completion, the reaction mixture was cooled to 0 °C and water was added dropwise. Then the aqueous layer was extracted with diethyl ether (3x). The organic layers were combined and washed with brine (3x). Then the organic layers were dried over Na_2SO_4, filtered, and concentrated. The crude mixture was purified by flash chromatography to provide the desired product.

General Procedure C: Alternative Ether Preparation

Diol **S3** (1 equiv) was added dropwise to a solution of NaH (60% oil dispersion, 1.2 equiv) in THF (0.33 M) at 0 °C. The mixture was stirred for 20 min, then the appropriate allylic or benzylic halide (1.1 equiv) was added dropwise. Upon completion, the reaction mixture was cooled to 0 °C and water was added dropwise. Then the aqueous layer was extracted with ethyl acetate (3x). The organic layers were combined and washed with brine (3x). Then the organic layers were dried over Na_2SO_4, filtered, and concentrated. The crude mixture was purified by flash chromatography to provide the desired product.

Experimental Protocols

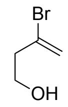

3-Bromobut-3-en-1-ol (S1)

To a solution of formalin (37% in H_2O, 2.9 mL, 1 equiv, 38 mmol) (*CAUTION* formalin is a sensitizer and is toxic. It must be handled in a hood), tin (5.94 g, 1.3 equiv, 50.0 mmol), and 2,3-dibromopropene (5.17 mL, 1.3 equiv, 50.0 mmol) in a 1:1 mixture of water and diethyl ether (83 mL each, 0.3 M), HBr (48% in H_2O, 43 µL, 0.01 equiv, 0.38 mmol) was added dropwise (*CAUTION* HBr is highly acidic and corrosive and can cause severe burns. Avoid direct contact with skin). After stirring for 20 h, the mixture was diluted with diethyl ether (20 mL) and filtered through Celite. The filtrate was extracted with diethyl ether (3 x 50 mL), then the organic layers were combined, dried over Na_2SO_4, filtered, and concentrated to an oil. This oil was purified via flash chromatography (20% Et_2O in pentanes) to yield **S1** (5.02 g, 86%,).

^{1}H NMR: ($CDCl_3$, 500 MHz) δ 5.71 (d, J = 1.4 Hz, 1H), 5.53 (d, J = 1.6 Hz, 1H), 3.81 (dt, J = 5.8, 5.8 Hz, 2H), 2.67 (t, J = 5.6 Hz, 2H), 1.65 (t, J = 5.6 Hz, 1H) ppm
^{13}C NMR: ($CDCl_3$, 125 MHz) δ 130.6, 119.5, 60.2, 44.6 ppm
HRMS: (ESI), *m/z* calcd for C_4H_6Br $[M – OH]^+$ 132.9647, found 132.9649
IR: (ATR, neat) 3429, 2926, 1720, 1373, 1259, 1095, 1024, 794 cm^{-1}

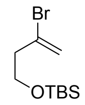

((3-Bromobut-3-en-1-yl)oxy)(*tert*-butyl)dimethylsilane (S2)

To a solution of **S1** (2.06 g, 1 equiv, 13.6 mmol) in CH_2Cl_2 (45.5 mL, 0.1 M) at 0 °C were added TBSCl (2.47 g, 1.2 equiv, 16.4 mmol) and imidazole (1.39 g, 1.5 equiv, 20.5 mmol). After 30 min the reaction was quenched with saturated NH_4Cl (20 mL) and the aqueous layer was extracted with CH_2Cl_2 (3 x 20 mL). The organic layers were combined, dried over Na_2SO_4, filtered, and concentrated to an oil. This oil was purified via flash chromatography (1% Et_2O in hexanes) to yield **S2** (3.33 g, 92%).

^{1}H NMR: ($CDCl_3$, 500 MHz) δ 5.62 (d, J = 0.9 Hz, 1H), 5.45 (d, J = 1.4 Hz, 1H), 3.79 (t, J = 6.3 Hz, 2H), 2.62 (t, J = 6.3 Hz, 2H), 0.89 (s, 9H), 0.07 (s, 6H) ppm
^{13}C NMR: ($CDCl_3$, 125 MHz) δ 130.9, 118.5, 61.0, 44.9, 26.0, 18.4, -5.2 ppm
IR: (ATR, neat) 2929, 2857, 1631, 1472, 1255, 1103, 914, 887, 836, 776, 744 cm^{-1}
HRMS: (ESI), *m/z* calcd for $C_{10}H_{22}OBrSi$ $[M + H]^+$ 265.0618, found 265.0619

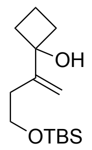

1-(4-((*tert*-butyldimethylsilyl)oxy)but-1-en-2-yl)cyclobutan-1-ol (9)

To a stirring solution of **S2** (2.00 g, 1 equiv, 7.54 mmol) in THF (38 mL, 0.2 M) at –78 °C, was added ^{n}BuLi (2.5 M in hexanes, 3.6 mL, 1.2 equiv, 9.1 mmol) dropwise (*CAUTION n*-Butyllithium is a strong base that reacts violently with water and other acids. Handle this agent under an inert atmosphere. After 15 min, cyclobutanone (0.67 g, 0.71 mL, 1.25 equiv, 9.4 mmol) was added dropwise and the reaction was warmed to 0 °C. When no starting material was observed via TLC (2-3 h), the reaction was quenched with saturated NH_4Cl (10 mL). The aqueous layer was extracted with diethyl ether (3 x 20 mL). The organic layers were combined, dried over Na_2SO_4, filtered, and concentrated to an oil. The crude oil was purified via flash chromatography (20% EtOAc in hexanes) to yield **9** (1.90 g, 98%).

^{1}H NMR: ($CDCl_3$, 500 MHz) δ) 5.16, (d, *J* = 0.7 Hz, 1H), 4.94 (d, *J* = 0.6 Hz, 1H), 4.26 (s, 1H), 3.77 (t, *J* = 5.8 Hz, 2H), 2.34 (t, *J* = 6.1 Hz, 2H), 2.25 (m, 2H), 2.13 (m, 2H), 1.80 (dt, *J* = 4.4, 11.0, Hz, 1H), 1.48 (dt, *J* = 8.3, 10.0 Hz, 1H), 0.89 (s, 9H), 0.06 (s, 6H) ppm
^{13}C NMR: ($CDCl_3$, 100 MHz) δ 150.5, 110.9, 77.4, 65.0, 35.3, 34.7, 26.0, 18.5, 12.8, –5.4 ppm
HRMS: (ESI), *m/z* calcd for $C_{14}H_{29}O_2Si$ 257.1931, found 257.1931
IR: (ATR, neat) 3425, 2956, 2932, 2869, 1642, 1410, 1258, 1086, 1022, 961, 899 cm^{-1}

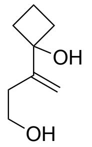

1-(3-Hydroxyprop-1-en-2-yl)cyclobutan-1-ol (S3)
To a stirring solution of **9** (2.98 g, 1 equiv, 11.6 mmol) in THF (89 mL, 0.13 M), was added Bu_4NF (1 M in THF, 11.6 mL, 1 equiv, 11.6 mmol) dropwise (*CAUTION* tetrabutylammonium fluoride (TBAF) is corrosive and an irritant towards skin and eyes and can cause burns. Avoid direct contact with skin). After 1 h the reaction was quenched with saturated NH_4Cl (50 mL) and the aqueous layer was extracted with diethyl ether (3 x 40 mL). The organic layers were combined, dried over Na_2SO_4, filtered, and concentrated to an oil. This oil was purified via flash chromatography (35% hexanes in EtOAc) to yield **S3** (1.59 g, 96%).

^{1}H NMR: ($CDCl_3$, 300 MHz) δ 5.19 (d, *J* = 0.8 Hz, 1H), 5.00 (d, *J* = 1.0 Hz, 1H), 3.81 (t, *J* = 5.9 Hz, 2H), 3.0-2.5 (br s, 2H), 2.39 (td, *J* = 1.1, 6.3 Hz, 2H), 2.36-2.27 (m, 2H), 2.16-2.03 (m, 2H), 1.95-1.81 (m, 1H), 1.64-1.48 (m, 1H) ppm
^{13}C NMR: ($CDCl_3$, 125 MHz) δ 149.7, 111.7, 77.7, 63.2, 35.1, 34.7, 13.0 ppm
HRMS: (ESI), *m/z* calcd for $C_8H_{15}O_2$ $[M + H]^+$ 143.1067, found 143.1068
IR: (ATR, neat) 3302, 2945, 1643, 1432, 1250, 1098, 1039, 958, 900 cm^{-1}

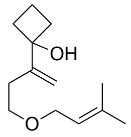

1-(4-((3-Methylbut-2-en-1-yl)oxy)but-1-en-2-yl)cyclobutan-1-ol (10)
Compound **10** was synthesized according to General Procedure **B** with **S3** (1.13 g, 7.95 mmol), NaH (382 mg, 15.9 mmol), and prenyl bromide (1.13 mL, 1.46 g, 9.78 mmol) in DMF (30 mL). The reaction was stirred for 2 h and then was purified by flash chromatography (20% Et_2O in hexanes) to yield **10** as an oil (1.34 g, 80%).

^{1}H NMR: ($CDCl_3$, 300 MHz) δ 5.31 (m, 1H), 5.16 (d, *J* = 1.0 Hz, 1H), 4.96 (d, *J* = 1.0 Hz, 1H), 3.97 (d, *J* = 6.9 Hz, 2H), 3.56 (t, *J* = 5.8 Hz, 2H), 2.38 (td, *J* = 1.0, 6.0 Hz, 2H), 2.32-2.21 (m, 2H), 2.18-2.05 (m, 2H), 1.90-1.76 (m, 1H), 1.73 (s, 3H), 1.65 (s, 3H), 1.58-1.42 (m, 1H) ppm
^{13}C NMR: ($CDCl_3$, 100 MHz) 160.3, 137.2, 120.4, 110.8, 77.0, 70.9, 67.7, 35.0, 32.1, 25.7, 25.6, 18.0, 12.7 ppm
HRMS: (ESI), *m/z* calcd for $C_{13}H_{23}O_2$ $[M + H]^+$ 211.1693, found 211.1691
IR: (ATR, neat) 3442, 2955, 2931, 2858, 1639, 1471, 1388, 1362, 1255, 1188, 1089, 1002, 901, 834, 776, 667 cm^{-1}

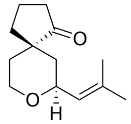

7-(2-methylprop-1-en-1-yl)-8-oxaspiro[4.5]decan-1-one (12)
To a solution of **10** (31.5 mg, 0.15 mmol) in CH_3CN (1.5 ml, 0.1 M) was added Bobbitt's Salt (67.5 mg, 0.23 mmol) in one portion. The reaction was stirred for 20 min then was quenched with water (1 ml). This mixture was extracted with Et_2O (1 x 1 mL) and the combined organic layers were washed with brine (14 ml) before being concentrated to a residue. This residue was purified by flash chromatography (1% Et_2O in methylene chloride) to afford **12** (23.1 mg, 74%).

One millimole scale: To a solution of **10** (210 mg, 1 mmol) in CH_3CN (10 ml, 0.1 M) was added Bobbitt's Salt (450 mg, 1.5 mmol) in one portion. The reaction was stirred for 15 min then was quenched with water (7 ml). This mixture was extracted with Et_2O (3 x 1 mL) and the combined organic layers were washed with brine (14 ml) before being concentrated to a residue. This residue was purified by flash chromatography (2% Et_2O in methylene chloride) to afford **12** (149 mg, 71%).

^{1}H NMR: ($CDCl_3$, 300 MHz) δ 5.06 (m, 1H), 4.59 (td, *J* = 2.4, 8.3 Hz, 1H), 3.96 (td, *J* = 1.6, 11.9 Hz, 1H), 3.80 (ddd, *J* = 1.7, 4.8, 11.5 Hz, 1H), 2.31 (td, *J* = Hz, 2H), 1.91 (m, 2H), 1.77 (m, 2H), 1.70 (d, *J* = Hz, 3H), 1.68 (d, *J* = Hz, 3H), 1.64-1.50 (m, 4H), 1.32 (dd, *J* = Hz, 1H) ppm
^{13}C NMR: ($CDCl_3$, 100 MHz) δ 222.0, 136.1, 125.8, 69.8, 63.5, 45.4, 40.1, 38.2, 37.9, 32.1, 25.7, 18.5, 18.1 ppm
HRMS: (ESI), *m/z* calcd for $C_{13}H_{21}O_2$ $[M + H]^+$ 209.1536, found 209.1530
IR: (ATR, neat) 2962, 1735, 1411, 1258, 1017, 863, 792, 703 cm^{-1}

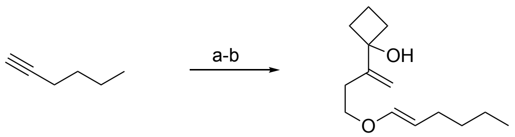

Reagents and conditions
a) Cp_2ZrCl_2, $LiEt_3BH$, THF, then I_2, 65%. b) **S3**, *trans*-N,N'-dimethyl-1,2-diaminocyclohexane, CuI, Cs_2CO_3, DME, 75 °C, 70%.

Scheme S1. Synthesis of **17**.

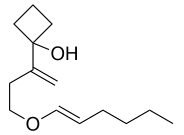

(*E*)-1-(4-(Hex-1-en-1-yloxy)but-1-en-2-yl)cyclobutan-1-ol (17)

A solution of **S3** (165 mg, 1.16 mmol), *trans-N,N'*-dimethyl-1,2-cyclohexane-diamine (66.0 mg, 0.47 mmol), (1*E*)-1-iodo-1-hexene (276 mg, 1.16 mmol),[1] and Cs_2CO_3 (1.13 g, 3.48 mmol) was added to a flask and purged with argon for 5 min. CuI (44 mg, 0.23 mmol) and DME (1.65 mL) were added and argon was bubbled though the solution for an additional 5 min. The solution was then heated to 75 °C using an oil bath and stirred for 22 h. The mixture was cooled to rt, diluted with diethyl ether, filtered through Celite, and concentrated. The crude mixture was purified via flash chromatography (10% EtOAc in hexanes) using silica pre-treated with 2% triethylamine in hexanes to yield **17** as a yellow oil (173 mg, 67%).

^{1}H NMR: ($CDCl_3$, 300 MHz) δ 6.20 (d, *J* = 12.7, 1H), 5.16 (s, 1H), 4.97 (s, 1H), 4.81 (dt, *J* = 7.3, 12.6 Hz, 1H), 3.80 (t, *J* = 7.0 Hz, 2H), 2.61 (s, 1H), 2.46 (t, *J* = 7.0 Hz, 2H), 2.30 (m, 2H), 2.09 (m, 2H), 1.89 (m, 3H), 1.55 (m, 1H), 1.3 (m, 4H), 0.88 (t, *J* = Hz, 2H) ppm
^{13}C NMR: ($CDCl_3$, 125 MHz) δ 149.2, 145.5, 110.9, 105.3, 77.8, 69.3, 35.0, 32.9, 31.1, 27.5, 22.2, 14.0, 13.0 ppm
HRMS: (ESI), *m/z* calcd for $C_{14}H_{25}O_2$ $[M + H]^+$ 225.1849, found 225.1854
IR: (ATR, neat) 3411, 2956, 2927, 2872, 1672, 1653, 1465, 1380, 1249, 1212, 1148, 1101, 1025, 959, 901, 802 cm^{-1}

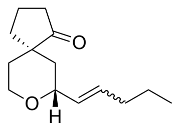

7-(Pent-1-en-1-yl)-8-oxaspiro[4.5]decan-1-one (19*EZ*)

Compound **19** was synthesized according to General Procedure A with **17** (50 mg, 0.22 mmol), 4 Å MS (50 mg), and DDQ (76 mg, 0.33 mmol) in CH_2Cl_2 (2.2 mL).

The oxidation step of the reaction reaction was stirred for 5 min, then the mixture was cooled to 0 °C. $Sc(OTf)_3$ (11.0 mg, 0.022 mmol) was added and the reaction was stirred for 40 min. The mixture was filtered through cotton and concentrated. This residue was purified by flash chromatography (20% Et_2O in hexanes) to yield the spirocycle as an inseparable mixture of *E/Z* isomers (34 mg, 68%, 4:1 *E/Z*) as an oil.

^{1}H NMR: ($CDCl_3$, 500 MHz, 4:1 mixture) δ 5.69 (dt, *J* = 6.7, 15.5 Hz, 0.8H, *E*-isomer), 5.47 (dt, *J* = 7.6, 10.7 Hz, 0.2H, *Z*-isomer), 5.38 (dd, *J* = 6.5, 15.5 Hz, 0.8H, *E* isomer), 5.30 (dd, *J* = 7.7, 9.6 Hz, 0.2H, *Z* isomer), 4.70 (t, *J* = 9.3 Hz, 0.2H, *Z*-isomer), 4.31 (t, *J* = 9.6 Hz, 0.8H, *E*-isomer), 3.96 (td, *J* = 2.9, 11.6 Hz, 1H), 3.83 (dd, *J* = 4.5, 10.9 Hz, 1H), 2.31 (t, *J* = 7.5 Hz, 2H), 2.14-1.96 (m, 2H), 1.96-1.88 (m, 2H), 1.83-1.73 (m, 2H), 1.67 (d, *J* = 13.7 Hz, 0.8H), 1.65-1.52 (m, 2.4H), 1.44-1.29 (m, 3H), 0.89 (t, *J* = 7.3 Hz, 3H) ppm
^{13}C NMR: ($CDCl_3$, 100 MHz, mixture of *E- and Z*-isomers) δ 222.1, 132.7, 131.0, 77.4, 73.4, 63.7, 45.5, 40.3, 38.4, 38.1, 34.6, 32.3, 22.4, 18.3, 13.9
HRMS: (ESI), *m/z* calcd for $C_{14}H_{23}O_2$ $[M + H]^+$ 223.1693, found 223.1697
IR: (ATR, neat) 2958, 1731, 1454, 1408, 1331, 1259, 1154, 1071, 1020, 968, 799 cm^{-1}

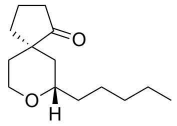

7-Pentyl-8-oxaspiro[4.5]decan-1-one (S4)
*The mixture of alkene isomers was subjected to hydrogenation to support the assignment of **19EZ** as alkene isomers.* To a stirring solution of **19*EZ*** (200 mg, 0.90 mmol) in MeOH (23 mL, 0.04 M) at 0 °C under an H_2 atmosphere, Pd/C (10% by weight, 191 mg) was added in one portion. The reaction stirred for 1 h, when it was determined complete by TLC analysis. The reaction was filtered through Celite and concentrated to a crude oil. This oil was purified via flash chromatography (10% EtOAc in hexanes) to yield **S4** (175 mg, 87%) as a single diastereomer.

^{1}H NMR: ($CDCl_3$, 300 MHz) δ 3.90 (td, *J* = 3.2, 11.7 Hz, 1H), 3.79 (m, 2H), 2.30 (t, *J* = 7.3 Hz, 2H), 1.92 (quintet, *J* = 7.2 Hz, 2H), 1.81-1.70 (m, 2H), 1.69-1.50 (m, 3H), 1.50-1.37 (m, 2H), 1.36-1.16 (m, 7H), 0.87 (t, *J* = 6.8 Hz, 3H) ppm
^{13}C NMR: ($CDCl_3$, 100 MHz) δ 222.2, 78.2, 72.8, 63.9, 45.6, 40.5, 38.5, 38.1, 32.7, 32.2, 25.3, 22.8, 18.3, 14.2 ppm
HRMS: (ESI), *m/z* calcd for $C_{14}H_{25}O_2$ $[M + H]^+$ 225.1849, found 225.1844
IR: (ATR, neat) 2929, 1730, 1408, 1353, 1264, 1153, 1093, 992, 804, 637, cm^{-1}

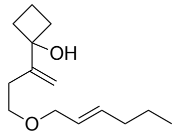

(*E*)-1-(4-((3-Methylhex-2-en-1-yl)oxy)but-1-en-2-yl)cyclobutan-1-ol (20)
This compound was synthesized according to General Procedure B with diol **S3** (100 mg, 0.70 mmol), NaH (60% dispersion, 34 mg, 0.84 mmol), and *(E)*-1-bromo-hex-2-ene[2] (126 mg, 0.77 mmol) in DMF (3.5 mL). The reaction was stirred for 2 h and then was purified by flash chromatography (20% Et_2O in hexanes) to yield **20** as an oil (66.9 mg, 42%).

^{1}H NMR: ($CDCl_3$, 300 MHz) δ 5.68 (dt, *J* = 6.6, 15.4 Hz, 1H), 5.52 (dt, *J* = 6.2, 15.4 Hz, 1H), 5.16 (d, *J* = 0.9 Hz, 1H), 4.96 (d, *J* = 0.9 Hz, 1H), 4.01 (s, 1H), 3.93 (dd, *J* = 1.1, 6.3 Hz, 2H), 3.56 (t, *J* = 5.8 Hz, 2H), 2.38 (t, *J* = 5.3 Hz, 2H), 2.26 (m, 2H), 2.12 (m, 2H), 2.01 (m, 2H), 1.83 (m, 1H), 1.51 (m, 2H), 1.39 (sextet, *J* = 7.2 Hz, 2H), 0.88 (t, *J* = 7.3 Hz, 3H) ppm

^{13}**C NMR:** ($CDCl_3$, 100 MHz) δ 150.3, 135.4, 125.8, 111.0, 77.1, 72.1, 70.9, 35.1, 34.5, 32.1, 22.3, 13.8, 12.9 ppm
HRMS: (ESI), *m/z* calcd for $C_{14}H_{25}O_2$ $[M + H]^+$ 225.1849, found 225.1848
IR: (ATR, neat) 3418, 2931, 2862, 1643, 1363, 1247, 1149, 1105, 1058, 970, 899 cm^{-1}

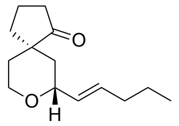

(*E*)-7-(Pent-1-en-1-yl)-8-oxaspiro[4.5]decan-1-one (19)
Compound **19** was synthesized according to General Procedure A with **20** (24.8 mg, 0.111 mmol), 4 Å MS (24.8 mg), and DDQ (37.9 mg, 1.67 mmol) in CH_2Cl_2 (1.11 mL). After 23 h, full conversion to the acetal was not seen so and additional DDQ (7.6 mg, 0.2 eq) was added. The reaction was stirred for an additional 3 h before $Sc(OTf)_3$ (5.4 mg, 011 mmol) was added. The mixture was stirred for 1 h, then was filtered through cotton and concentrated to a residue. This residue was purified by flash chromatography (5% Et_2O in hexanes) to yield **19** (16.6 mg, 67%) as an oil along with recovered **19** (3 mg, 72% based on recovered starting material).

^{1}**H NMR:** ($CDCl_3$, 300 MHz) δ 5.69 (dt, *J* = 6.7, 15.5 Hz, 1H), 5.38 (dd, *J* = 6.6, 15.5 Hz, 1H), 4.31 (dd, *J* = 7.5, 9.9Hz, 1H), 3.97 (td, *J* = 3.4, 11.6 Hz, 1H), 3.83 (dd, *J* = 3.6, 10.2 Hz, 1H), 2.32 (t, *J* = 7.5 Hz, 2H), 2.04-1.86 (m, 4H), 1.78 (m, 2H), 1.70-1.51 (m, 3H), 1.44-1.22 (m, 3H), 0.88 (t, *J* = 7.4 Hz, 3H) ppm
^{13}**C NMR:** ($CDCl_3$, 100 MHz) δ 222.1, 132.6, 130.9, 73.3, 63.7, 45.5, 40.3, 38.4, 38.1, 34.6, 32.3, 22.3, 18.3, 13.8 ppm
HRMS: (ESI), *m/z* calcd for $C_{14}H_{23}O_2$ $[M + H]^+$ 223.1693, found 223.1686
IR: (ATR, neat) 2957, 2928, 2870, 1730, 1455, 1260, 1154, 1071, 1022, 968 cm^{-1}

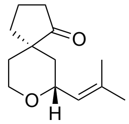

7-(2-methylprop-1-en-1-yl)-8-oxaspiro[4.5]decan-1-one (12)
Compound **12** was synthesized according to General Procedure A with **10** (50 mg, 0.24 mmol), 4 Å MS (50 mg), and DDQ (81 mg, 0.36 mmol) in CH_2Cl_2 (2.4 mL). The oxidation step of the reaction was stirred for 20 h, then the mixture was cooled to 0 °C. $Sc(OTf)_3$ (11.7 mg, 0.02 mmol) was added and the reaction was stirred for 30 min. The mixture was filtered through cotton and concentrated to a residue. This residue was purified by flash chromatography (20% Et_2O in hexanes) to yield **12** (34 mg, 68%) as an oil. Spectral data matched those from the compound reported above.

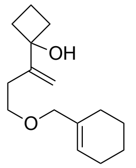

1-(4-(Cyclohex-1-en-1-ylmethoxy)but-1-en-2-yl)cyclobutan-1-ol (21)
This compound was synthesized according to General Procedure **B** with **S3** (55.7 mg, 0.392 mmol), NaH (18.8 mg, 0.470 mmol), and 1-bromomethyl cyclohexene[3] (75.5 mg, 0.431 mmol) in THF (1.19 ml, .33 M) The reaction was stirred for 19 h and then was purified by flash chromatography (10% EtOAc in hexanes) to afford **21** (48.6 mg, 53%) as a clear oil.

^{1}**H NMR:** ($CDCl_3$, 500 MHz) δ 5.65 (s, 1H), 5.16 (s, 1H), 4.96 (s, 1H), 4.05 (s, 1H), 3.83 (s, 2H), 3.51 (t, J = 5.94 Hz, 2H), 2.38 (t, *J* = 5.90 Hz, 2H), 2.28-2.23 (m, 2H), 2.14-2.08 (m, 2H), 2.05-1.98 (m, 2H), 1.98-1.93 (m, 2H) 1.87-1.78 (m, 1H), 1.65-1.1.59 (m, 2H), 1.59-1.54 (m, 2H), 1.65-1.45 (m, 1H)
^{13}**C NMR:** ($CDCl_3$, 100 Hz) δ 150.3, 134.4, 125.4, 110.8, 76.9, 76.1, 70.7, 35.0, 31.9, 25.9, 25.0, 22.5, 22.3, 12.7 ppm

HRMS: (ESI), *m/z* calcd for $C_{15}H_{24}O_2Na$ $[M+Na]^+$ 259.1669, found 259.1677
IR: (ATR, neat): 3412, 2928, 2857, 1642, 1357, 1150, 1081, 961, 829 cm^{-1}

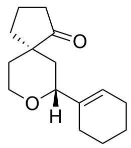

22

(5*R*,7*R*)-7-(cyclohex-1-en-1-yl)-8-oxaspiro[4.5]decan-1-one (22)
This compound was synthesized according to General Procedure A with **21** (25.4 mg, 0.107 mmol), 4 Å MS (25.4 mg), and DDQ (36.5 mg, 0.161 mmol) in CH_2Cl_2 (1.07 mL). The oxidation step of the reaction was stirred for 18 h. $Sc(OTf)_3$ was added (5.27 mg, 0.011 mmol) and the reaction was stirred 1 h The mixture was filtered through Celite and cotton and concentrated to a residue. This residue purified by flash chromatography (5% EtOAc in hexanes) to afford an 11:1 diastereomeric mixture of **22** (16.9 mg, 67%) as a yellow oil.

^{1}H NMR: ($CDCl_3$, 400 MHz) δ 5.69 (s, 1H), 4.92* (dd, *J* = 4.7, 11.1 Hz, .09H), 3.98 (td, *J* = 3.3, 12.0 Hz, 1H) 3.84 (ddd, *J* = 1.7, 5.0, 11.3 Hz, 1H), 2.31 (t, J = 7.5 Hz, 2H) 2.04-1.96 (m, 4H), 1.92 (p, *J* = 7.2 Hz, 2H) 1.78 (t, *J* = 6.5 Hz, 1H), 1.81-1.75 (m, 2H), 1.67 (dt, *J* = 2.2, 13.6 Hz, 1H) 1.64- 1.50 (m, 7H), 1.43 (dd, *J* = 11.7, 13.7 Hz, 1H) ppm
^{13}C NMR: ($CDCl_3$, 100 MHz) δ 211.9, 138.3, 122.9, 76.3, 63.9, 45.5, 40.3, 36.4, 32.3, 25.0, 24.7, 22.7, 22.5, 18.1
HRMS: *m/z* calcd for $C_{15}H_{23}O_2$ $[M+H]^+$ 235.1693, found 235.1709
IR: (ATR, neat) 2990, 2931, 2870, 1731, 1450, 1381, 1262, 1143, 1071 cm^{-1}
* Diagnostic peak from the minor diastereomer

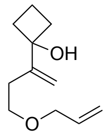

1-(4-(Allyloxy)but-1-en-2-yl)cyclobutan-1-ol (14)
This compound was synthesized according to General Procedure B with diol **S3** (150 mg, 1.05 mmol), NaH (51 mg, 1.3 mmol), and allyl bromide (140 mg, 1.16 mmol) in DMF (5.3 mL). The reaction was stirred for 1 h and then was purified by flash chromatography (20% Et_2O in hexanes) to yield **14** as an oil (129 mg, 67%).

^{1}H NMR: ($CDCl_3$, 400 MHz) δ 5.88 (ddt, *J* = 5.7, 10.8, 16.2 Hz, 1H), 5.26 (dd, *J* = 1.0, 17.2 Hz, 1H), 5.18 (dd, *J* = 0.6, 12.5 Hz, 1H), 5.16 (d, *J* = 0.5 Hz, 1H), 4.97 (d, *J* = 0.9 Hz, 1H), 3.99 (d, *J* = 5.6 Hz, 2H), 3.88 (bs, 1H), 3.59 (t, *J* = 5.9 Hz, 2H), 2.40 (t, *J* = 6.0 Hz, 2H), 2.30-2.24 (m, 2H), 2.15-2.07 (m, 2H), 1.88-1.78 (m, 1H), 1.58-1.45 (m, 1H) ppm
^{13}C NMR: ($CDCl_3$, 125 MHz) δ 150.2, 134.2, 117.6, 111.1, 77.2, 72.2, 71.3, 35.1, 32.0, 12.9 ppm
HRMS: (ESI), *m/z* calcd for $C_{11}H_{19}O_2$ [M + H]$^+$ 183.1380, found 183.1381
IR: (ATR, neat) 3353, 2957, 1723, 1642, 1409, 1257, 1145, 1016, 902, 864, 792 cm^{-1}

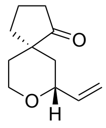

7-Vinyl-8-oxaspiro[4.5]decan-1-one (16)
Compound **15** was synthesized according to General Procedure A, though with the oxidation step being conducted at 40 °C with **14** (62.9 mg, 0.345 mmol), 4Å MS (62.9 mg), and DDQ (117 mg, 0.518 mmol) in DCM (3.45 mL) for 23 h. The mixture was cooled to 0 °C. $Sc(OTf)_3$ (17.7 mg, 0.04 mmol) was added and the reaction was stirred for 1 h. The mixture was filtered through cotton and concentrated to a residue. This residue was purified by flash chromatography (20% Et_2O in hexanes) to yield **15** (29%, 18.8 mg, 0.10 mmol) as an oil.

^{1}H NMR: ($CDCl_3$, 300 MHz) δ 5.78 (ddd, *J* = 5.7, 10.6, 16.6 Hz, 1H), 5.24 (d, *J* = 17.2 Hz, 1H), 5.10 (d, *J* = 10.5 Hz, 1H), 4.39 (dd, *J* = 5.8, 11.2 Hz, 1H), 3.97 (td, *J* = 3.3, 11.6 Hz, 1H), 3.85

(ddd, $J = 2.1, 4.6, 11.6$ Hz, 1H), 2.33 (t, $J = 7.2$ Hz, 2H), 1.93 (quintet, $J = 7.2$ Hz, 2H), 1.83-1.51 (m, 5H), 1.32 (dd, $J = 11.6, 13.6$ Hz, 1H) ppm
^{13}C NMR: ($CDCl_3$, 100 MHz) δ 221.9, 139.0, 115.1, 77.2, 73.2, 63.5, 45.3, 40.0, 37.9, 37.8, 32.1, 30.9, 18.2 ppm
HRMS: (ESI), *m/z* calculated for $[M + H]^+$ 181.1223, found 181.1224
IR: (ATR, neat) 2952, 2868, 1727, 1647, 1407, 1264, 1159, 1083, 922, 832 cm^{-1}

Isomerization experiment: To a stirring solution of **22** (18.5 mg, 0.079 mmol, >20:1 d.r.) in CH_2Cl_2 (0.79 mL, 0.1 M) was added $Sc(OTf)_3$ (3.9 mg, 0.0079 mmol) at rt. After 23 h the mixture was filtered through Celite and concentrated without further purification to give **22** as a 9:1 mixture of diastereomers.

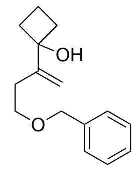

1-(4-(Benzyloxy)but-1-en-2-yl)cyclobutan-1-ol (23)
This compound was synthesized according to General Procedure B with diol **S3** (400 mg, 2.81 mmol), NaH (60% dispersion, 135 mg, 3.38 mmol), and benzyl bromide (0.37 mL, 3.09 mmol) in DMF (14 mL). The reaction was stirred for 2 h and then was purified by flash chromatography (20% Et_2O in hexanes) to yield **23** as an oil (387 mg, 59%).

^{1}H NMR: ($CDCl_3$, 500 MHz) δ 7.36-7.27 (m, 5H), 5.16 (d, $J = 0.5$ Hz, 1H), 5.00 (d, $J = 0.7$ Hz, 1H), 4.53 (s, 2H), 3.68 (s, 1H), 3.63 (t, $J = 5.6$ Hz, 2H), 2.42 (td, $J = 0.6, 6.1$ Hz, 2H), 2.30-2.25 (m, 2H), 2.14-2.08 (m, 2H), 1.87-1.79 (m, 1H), 1.54-1.47 (m, 1H) ppm
^{13}C NMR: ($CDCl_3$, 125 MHz) δ 150.1, 137.8, 128.6, 127.9, 127.8, 111.0, 77.3, 73.4, 71.3, 35.1, 31.9, 12.9 ppm
HRMS: (ESI), *m/z* calcd for $C_{15}H_{21}O_2$ 233.1536, found 233.1536
IR: (ATR, neat) 3392, 2951, 1720, 1453, 1275, 1098, 1070, 1026, 906, 800 cm^{-1}

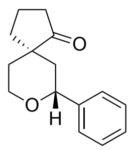

7-Phenyl-8-oxaspiro[4.5]decan-1-one (24)
This compound was synthesized according to General Procedure A with **23** (100 mg, 0.43 mmol), 4 Å MS (100 mg), and DDQ (147 mg, 0.65 mmol) in DCE (4.3 mL) except that the oxidation step was heated conducted 40 °C for 20 h. The mixture was cooled to 0 °C and $Sc(OTf)_3$ (21 mg, 0.04 mmol) was added and the reaction was stirred for 30 min. The mixture was filtered through cotton and concentrated. The resulting residue was purified by flash chromatography (20% Et_2O in hexanes) to yield **24** (75 mg, 76%) as an oil.

^{1}H NMR: ($CDCl_3$, 500 MHz) δ 7.37-7.30 (m, 4H), 7.26 (m, 1H), 4.94 (dd, $J = 2.3, 11.6$ Hz, 1H), 4.11 (td, $J = 4.3, 11.6$ Hz, 1H), 3.97 (ddd, $J = 1.8, 4.6, 11.6$ Hz, 1H), 2.36 (td, $J = 2.7, 7.5$ Hz, 2H), 1.99-1.90 (m, 2H), 1.89-1.75 (m, 3H), 1.73-1.67 (m, 2H), 1.55 (dd, $J = 11.6, 13.7$ Hz, 1H) ppm
^{13}C NMR: ($CDCl_3$, 100 MHz) δ 222.2, 143.0, 128.5, 127.6, 126.0, 74.9, 64.3, 45.9, 40.4, 40.3, 38.2, 32.3, 18.3 ppm
HRMS: (ESI), *m/z* calcd for $C_{15}H_{19}O_2$ $[M + H]^+$ 231.1380, found 231.1372
IR: (ATR, neat) 2954, 1728, 1494, 1406,1261, 1211, 1154, 1070, 1026, 991, 912 cm^{-1}

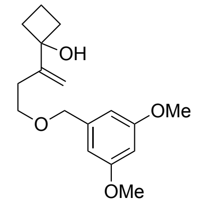

1-(4-((3,5-Dimethoxybenzyl)oxy)but-1-en-2-yl)cyclobutan-1-ol (25)
This compound was synthesized according to General Procedure B with diol **S3** (80 mg, 0.56 mmol), NaH (27 mg, 0.68 mmol), and 3,5-dimethoxybenzyl bromide[4] (143 mg, 0.619 mmol) in DMF (2.8 mL). The reaction was stirred for 1 h and then was purified by flash chromatography (20% Et_2O in hexanes) to yield **25** as an oil (109 mg, 66%).

^{1}H NMR: ($CDCl_3$, 300 MHz) δ 6.46 (d, J = 2.0 Hz, 2H), 6.38 (t, J = 2.1 Hz, 1H), 5.16 (d, J = 0.6 Hz, 1H), 4.96 (d, J = 0.6 Hz, 1H), 4.47 (s, 2H), 3.78 (s, 6H), 3.62 (m, 3H), 2.42 (t, J = 6.0 Hz, 2H), 2.33-2.22 (m, 2H), 2.18-2.04 (m, 2H), 1.91-1.76 (m, 1H), 1.59-1.43 (m, 1H) ppm
^{13}C NMR: ($CDCl_3$, 125 MHz) δ 160.9, 149.9, 140.1, 110.8, 105.5, 99.9, 77.2, 73.1, 71.0, 55.3, 35.0. 31.7, 12.8 ppm
HRMS: (ESI), *m/z* calcd for $C_{17}H_{25}O_4$ $[M + H]^+$ 293.1747, found 293.1738
IR: (ATR, neat) 3418, 2939, 1597, 1461, 1368, 1297, 1203, 1100, 960, 903 cm^{-1}

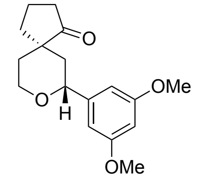

7-(3,5-Dimethoxyphenyl)-8-oxaspiro[4.5]decan-1-one (26)
This compound was synthesized according to General Procedure A with **25** (80 mg, 0.27 mmol), 4Å MS (80 mg), and DDQ (93 mg, 0.41 mmol) in CH_2Cl_2 (2.7 mL). The oxidation reaction was stirred for 21 h, then the mixture was cooled to 0 °C. $Sc(OTf)_3$ (13.5 mg, 0.03 mmol) was added and the reaction was stirred for 30 min. The mixture was filtered through cotton and concentrated. The resulting residue was purified by flash chromatography (20% Et_2O in hexanes) to yield **26** (63 mg, 80%) as an oil.

^{1}H NMR: ($CDCl_3$, 300 MHz) δ 6.51 (d, J = 2.0 Hz, 2H), 6.36 (t, J = 2.1 Hz, 1H), 4.87 (dd, J = 2.2, 11.4 Hz, 1H), 4.13-4.02 (m, 1H), 3.96 (m, 1H), 3.78 (s, 6H), 2.36 (t, J = 7.2 Hz, 2H), 1.99-1.86 (m, 3H), 1.85-1.74 (m, 2H), 1.73-1.64 (m, 2H), 1.53 (dd, J = 11.6, 13.7 Hz, 1H) ppm
^{13}C NMR: ($CDCl_3$, 100 MHz) δ 222.1, 160.9, 145.5, 103.8, 99.9, 75.0, 64.2, 55.5, 45.9, 40.3, 40.2, 38.2, 32.3, 18.3 ppm
HRMS: (ESI), *m/z* calcd for $C_{17}H_{23}O_4$ $[M + H]^+$ 291.1591, found 291.1580
IR: (ATR, neat) 2961, 1727, 1600, 1413, 1258, 1148, 1015, 863, 788, 701 cm^{-1}

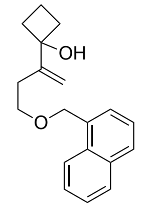

1-(4-(Naphthalen-1-ylmethoxy)but-1-en-2-yl)cyclobutan-1-ol (27)
This compound was synthesized according to General Procedure B with diol **S3** (150 mg, 1.05 mmol), NaH (51 mg, 1.3 mmol), and 1-(bromomethyl)naphthalene (257 mg, 1.16 mmol) in DMF (5.3 mL). The reaction was stirred for 1 h and then was purified by flash chromatography (20% Et_2O in hexanes) to yield **27** as an oil (275 mg, 92%).

^{1}H NMR: ($CDCl_3$, 500 MHz) δ 8.07 (d, J = 8.2 Hz, 1H), 7.87 (d, J = 9.2 Hz, 1H), 7.82 (d, J = 7.9 Hz, 1H), 7.56-7.41 (m, 4H), 5.16 (d, J = 0.9 Hz, 1H), 4.99 (s, 2H), 4.96 (d, J = 0.9 Hz, 1H), 3.70 (t, J = 6.2 Hz, 2H), 3.53 (bs, 1H), 2.44 (td, J = 0.9, 6.2 Hz, 2H), 2.30-2.23 (m, 2H), 2.13-2.05 (m, 2H), 1.87-1.78 (m, 1H), 1.56 (m, 1H) ppm
^{13}C NMR: ($CDCl_3$, 125 MHz) δ 150.0, 133.9, 133.3, 131.9, 128.9, 128.7, 126.6, 126.4, 125.9, 125.3, 124.0, 110.8, 77.3, 71.8, 71.1, 35.1, 31.8 ppm
HRMS: (ESI), *m/z* calcd for $C_{19}H_{21}O$ $[M – OH]^+$ 265.1588, found 265.1582
IR: (ATR, neat) 3401, 3048, 2940, 2864, 1642, 1511, 1247, 1092, 1045, 960, 900 cm^{-1}

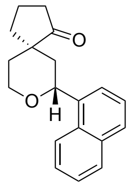

7-(Naphthalen-1-yl)-8-oxaspiro[4.5]decan-1-one (28)

This compound was synthesized according to General Procedure A with **27** (80 mg, 0.28 mmol), 4 Å MS (80 mg), and DDQ (96 mg, 0.43 mmol) in CH_2Cl_2 (2.8 mL). The oxidation reaction was stirred for 22 h, then the mixture was cooled to 0 °C. $Sc(OTf)_3$ (13.9 mg, 0.03 mmol) was added and the reaction was stirred for 30 min. The mixture was filtered through cotton and concentrated to a residue. This residue was purified by flash chromatography (20% Et_2O in hexanes) to yield **28** (74 mg, 93%) as a white solid.

Ome mmol scale: To a solution of **27** (282 mg, 1 mmol) and 4 Å MS (282 mg) stirring in DCM (10 ml) was added DDQ (341 mg, 1.5 eq). The mixture stirred for 22 h at rt, then was cooled to 0 °C and $Sc(OTf)_3$ (49 mg, 0.1 eq) was added. The mixture was warmed to rt and stirred for an additional 2.5 h, then was filtered through Celite and concentrated to a residue. This residue was purified by flash chromatography (20% Et_2O in hexanes) to yield **28** as a white solid (256 mg, 91%).

^{1}H NMR: ($CDCl_3$, 400 MHz) δ 8.05 (d, J = 8.4 Hz, 1H), 7.84 (d, J = 7.8 Hz, 1H), 7.74 (d, J = 8.1 Hz, 1H), 7.65 (d, J = 7.1 Hz, 1H), 7.53-7.43 (m, 3H), 5.84 (d, J = 10.1 Hz, 1H), 4.21 (td, J = 3.1, 11.7 Hz, 1H), 4.10 (dd, J = 3.7, 10.8 Hz, 1H), 2.50-2.34 (m, 2H), 2.08 (d, J = 13.9 Hz, 1H), 2.03-1.90 (m, 2H), 1.88-1.74 (m, 4H), 1.64 (dd, J = 11.6, 13.8 Hz, 1H) ppm
^{13}C NMR: ($CDCl_3$, 100 MHz) δ 222.5, 139.1, 133.8, 130.3, 128.9, 127.8, 126.2, 125.7, 125.5, 123.4, 122.6, 71.9, 63.8, 46.2, 40.2, 40.0, 38.1, 32.6, 18.3 ppm
HRMS: (ESI), *m/z* calcd for $C_{19}H_{21}O_2$ $[M + H]^+$ 281.1536, found 281.1526
IR: (ATR, neat) 2962, 1726, 1698, 1599, 1520, 1408, 1345, 1258, 1072, 1014, 862 cm^{-1}
Mp: 119.0 – 120.3 °C

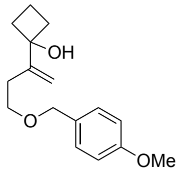

1-(4-((4-Methoxybenzyl)oxy)but-1-en-2-yl)cyclobutan-1-ol (29)

This compound was synthesized according to General Procedure B with diol **S3** (200 mg, 1.41 mmol), NaH (67.5 mg, 1.69 mmol), and *p*-methoxybenzyl bromide (311 mg, 1.55 mmol) in DMF (7.0 mL). The reaction was stirred for 1 h and then was purified by flash chromatography (20% Et_2O in hexanes) to yield **29** an oil (277 mg, 75%).

^{1}H NMR: ($CDCl_3$, 300 MHz) δ 7.23 (d, J = 8.6 Hz, 2H), 6.87 (d, J = 8.6 Hz, 2H), 5.15 (d, J = 0.9 Hz, 1H), 4.95 (d, J = 1.0 Hz, 1H), 4.45 (s, 2H), 3.80 (s, 4H), 3.60 (t, J = 5.8 Hz, 2H), 2.40 (td, J = 0.9, 6.1 Hz, 2H), 2.32-2.21 (m, 2H), 2.17-2.04 (m, 2H), 1.90-1.75 (m, J = Hz, 1H), 1.57-1.42 (m, 1H) ppm
^{13}C NMR: ($CDCl_3$, 100 MHz) δ 159.3, 150.1, 129.7, 129.4, 113.9, 110.9, 77.2, 72.9, 55.3, 35.0, 31.9, 12.7 ppm
HRMS: (ESI), *m/z* calcd for $C_{16}H_{23}O_3$ $[M + H]^+$ 263.1642, found 263.1636
IR: (ATR, neat) 3396, 2938, 2864, 1611, 1586, 1513, 1361, 1247, 1148, 1089, 1032, 961 cm^{-1}

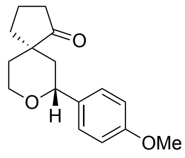

7-(4-Methoxyphenyl)-8-oxaspiro[4.5]decan-1-one (30)

This compound was synthesized according to General Procedure A with **29** (69 mg, 0.26 mmol), 4Å MS (69 mg), and DDQ (90 mg, 0.40 mmol) in CH_2Cl_2 (2.6 mL). The oxidation step was stirred for 1 h, then the reaction was cooled to 0 °C and $Sc(OTf)_3$ (12.9 mg, 0.03 mmol) was added. The mixture was stirred for 30

min, then was filtered through cotton and concentrated to a residue. This residue was purified by flash chromatography (20% Et_2O in hexanes) to yield **30** (43 mg, 63%) as an oil.

^{1}H NMR: ($CDCl_3$, 300 MHz) δ 7.27 (d, *J* = 8.6, Hz, 2H), 6.85 (d, *J* = 8.7 Hz, 2H), 4.87 (dd, *J* = 2.3, 11.6 Hz, 1H), 4.14-4.06 (m, 1H), 3.94 (ddd, *J* = 2.1, 4.4, 11.6 Hz, 1H), 3.79 (s, 3H), 2.35 (td, *J* = 1.2, 7.5 Hz, 2H), 1.99-1.89 (m, 2H), 1.86-1.74 (m, 3H), 1.41-1.64 (m, 2H), 1.55 (dd, *J* = 11.6, 13.8 Hz, 1H) ppm
^{13}C NMR: ($CDCl_3$, 125 MHz) δ 222.2, 159.1, 135.2, 127.3, 113.9, 74.6, 64.3, 55.4, 46.0, 40.3, 40.2, 38.2, 32.4, 18.3 ppm
HRMS: (ESI), *m/z* calcd for $C_{16}H_{21}O_3$ $[M + H]^+$ 261.1485, found 261.1478
IR: (ATR, neat) 2951, 1726, 1613, 1586, 1461, 1332, 1244, 1154, 1089, 1070, 1031, 902 cm^{-1}

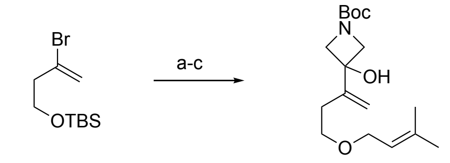

Reagents and conditions
a) ^{n}BuLi, THF, –78 °C, then 1-Boc-3-oxoazetidine, 0 °C, 98%. b) Bu_4NF, THF, 0 °C 96%. NaH, DMF, then prenyl bromide, 74%.

Scheme S2. Synthesis of a heterocyclic substrate.

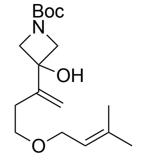

***tert*-Butyl 3-hydroxy-3-(4-((3-methylbut-2-en-1-yl)oxy)but-1-en-2-yl)azetidine-1-carboxylate (31)**

^{1}H NMR: ($CDCl_3$, 400 MHz) δ 5.31 (t, *J* = 6.8 Hz, 1H), 5.28 (s, 1H), 5.22 (s, 1H), 4.03 (d, *J* = 9.2 Hz, 2H), 3.95 (d, *J* = 9.3 Hz, 2H), 3.75 (m, 4H), 2.33 (t, *J* = 6.3 Hz, 2H), 1.97 (bs, 1H), 1.75 (s, 3H), 1.66 (s, 3H), 1.44 (s, 9H) ppm
^{13}C NMR: ($CDCl_3$, 75 MHz) δ 119.8, 79.4, 77.2, 71.5, 67.7, 33.1, 28.4, 25.7, 18.1 ppm
HRMS: (ESI), *m/z* calcd for $C_{17}H_{30}O_4N$ [M+H] 312.2169, found 312.2164
IR: (ATR, neat) 3384, 2974, 2931, 1703, 1678, 1394, 1255, 1080, 906, 770 cm^{-1}

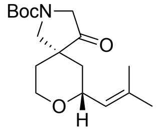

***tert*-Butyl 7-(2-methylprop-1-en-1-yl)-4-oxo-8-oxa-2-azaspiro[4.5]decane-2-carboxylate (32)**

Compound **32** was synthesized according to General Procedure A with **31** (83.4 mg, 0.27 mmol), 4Å MS (83.4 mg), and DDQ (91.3 mg, 0.40 mmol) in CH_2Cl_2 (2.7 mL). The reaction was stirred for 26 h, then the reaction was cooled to 0 °C and $Sc(OTf)_3$ (13.2 mg, 0.027 mmol) was added and stirred for 30 min. The reaction was filtered through cotton and concentrated to a residue. This residue was purified by flash chromatography (20% Et_2O in hexanes) to yield **32** as an inseparable mixture of diastereomers as a clear oil (43%, 35.6 mg, 0.12 mmol, 5:1).

^{1}H NMR: ($CDCl_3$, 300 MHz) δ 5.08 (d, *J* = 7.5 Hz, 1H), 4.55 (t, *J* = 9.6 Hz, 1H), 3.88 (m, 4H), 3.57-3.40 (m, 2H), 1.71 (m, 10H), 1.48 (s, 9H) ppm
^{13}C NMR: (Acetond-*d*6, 100 MHz) δ 219.0, 159.3, 139.1, 131.6, 84.3, 75.9, 75.0, 68.8, 68.0, 41.6, 36.4, 32.8, 29.9 ppm
HRMS: (ESI), *m/z* calcd for $C_{17}H_{27}NO_4$ $[M + H]^+$ 310.2013, found 310.2026
IR: (ATR, neat) 2972, 2931, 1751, 1702, 1478, 1367, 1260, 1134, 1072, 1007 cm^{-1}

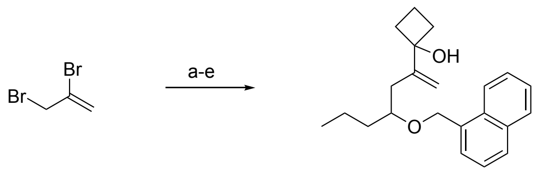

Reagents and conditions
a) Sn, PrCHO, HBr, Et_2O, H_2O, 86%. b) TBSCl, imidazole, CH_2Cl_2, 92%.
c) ^{n}BuLi, THF, –78 °C, then cyclobutanone, 0 °C, 98%. d) Bu_4NF, THF,
0 °C, 96%. e) NaH, DMF, then 1-bromomethylnaphthalene, 30%.

Scheme S3. Synthesis of a branched substrate.

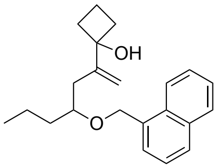

1-(4-(Naphthalen-1-ylmethoxy)hept-1-en-2-yl)cyclobutan-1-ol (33)

This compound was synthesized according to General Procedure **C** with the appropriate diol (119 mg, 1.08 mmol), NaH (52 mg, 1.30 mmol), and 1-bromomethylnapthalene (263 mg, 1.19 mmol) in THF (3.3 ml, .33 M). The reaction was stirred for 20 h, then was purified by flash chromatography (15% EtOAc in hexanes) to afford **33** as a separable mixture (5:1 2° ether:3° ether) of regioisomers (221 mg, 63%) as a clear oil.

^{1}H NMR: ($CDCl_3$, 300 MHz) δ 8.02 (d, *J* = 8.1 Hz, 1H), 7.50 (m, 3H), 7.42 (dd, *J* = 7.0, 7.6 Hz, 1H) 5.14 (s, 1H), 5.02 (d, *J* = 11.7 Hz, 1H), 4.94 (d, *J* = 13.1 Hz, 1H), 4.92 (s, 1H), 4.0 (s, 1H), 2.42 (dd, *J* = 3.9, 14.4 Hz, 1H), 2.33 (dd, *J* = 9.9, 14.4 Hz, 1H), 2.26 (m, 1H), 2.15 (m, 1H), 2.07 (dd, *J* = 8.9, 18.5 Hz, 1H), 2.01 (dd, *J* = 11.1, 20.4 Hz, 1H), 1.77 (m, 1H), 1.67 (m, 1H), 1.57 (m, 1H), 1.47 (dd, *J* = 8.5, 16.9 Hz, 1H), 1.38 (sextet, *J* = 7.4 Hz, 2H), 0.89 (t, *J* = 8.4 Hz, 3H) ppm
^{13}C NMR: ($CDCl_3$, 125 MHz) δ 149.2, 133.9, 133.6, 131.8, 128.8, 128.6, 126.7, 126.3, 125.9, 125.4, 124.2, 110.6, 80.4, 69.7, 36.0, 35.8, 35.6, 34.8, 18.7, 14.3, 12.8 ppm
HRMS: (ESI), *m/z* calcd for $C_{22}H_{29}O_2$ $[M + H]^+$ 325.2162, 325.2152
IR: (ATR, neat) 3384, 2958, 2938, 2871, 1641, 1511, 1463, 1260, 1087, 1029, 902, 860 cm^{-1}

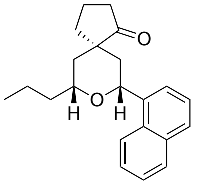

7-(Naphthalen-1-yl)-9-propyl-8-oxaspiro[4.5]decan-1-one (35)

This compound was synthesized according to General Procedure A with **33** (34.4 mg, 0.075 mmol), 4 Å MS (34.4 mg), and DDQ (25.7 mg, 0.113 mmol) in CH_2Cl_2 (0.75 mL). The oxidation step of the reaction was stirred for 20 h. $Sc(OTf)_3$ was added (3.69 mg, 0.0075 mmol) and the reaction was stirred for 2 h. The mixture was filtered through celite and cotton and concentrated to a residue. This residue purified by flash chromatography (5% EtOAc in hexanes) to yield a clear oil (24.3 mg, 71%).

^{1}H NMR: ($CDCl_3$, 500 MHz) δ 8.02 (d, *J* = 8.1 Hz, 1H), 7.84 (d, *J* = 7.7Hz, 1H), 7.75 (d, *J* = 8.1 Hz, 1H), 7.65 (d, *J* = 7.5 Hz, 1H), 7.54-7.42 (m, 3H), 5.79 (d, *J* = 10.4 Hz, 1H), 4.19-4.10 (m, 1H), 2.51-2.33 (m, 2H), 2.09 (dt, *J* = 2.0, 13.9 Hz, 1H), 2.04-1.88 (m, 2H), 1.88-1.74 (m, 3H), 1.66-1.40 (m, 6H), 1.36 (dd, *J* = 11.6, 13.7 Hz, 1H), 0.93 (t, *J* = 7.0 Hz, 3H) ppm
^{13}C NMR: ($CDCl_3$, 100 MHz) δ 221.4, 138.0, 132.6, 129.4, 127.6, 124.9, 124.3, 122.5, 121.5, 72.1, 70.3, 45.7, 39.1, 38.3, 37.7, 36.9, 28.7, 17.6, 17.2, 13.2 ppm
HRMS: (ESI), *m/z* calcd for $C_{22}H_{27}O_2$ $[M + H]^+$ 323.2006, found 323.1994
IR: (ATR, neat) 2962, 1729, 1410, 1258, 1014, 862, 789 cm^{-1}

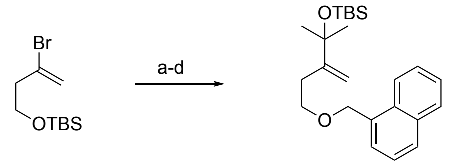

Reagents and conditions
a) ^{n}BuLi, THF, –78 °C, then acetone, 0 °C, 98%. b) Bu_4NF, THF, 0 °C 96%. NaH, DMF, then 1-bromomethylnaphthalene, 60%. d) TBSOTf, 2,6-lutidine, 0 °C, 82%.

Scheme S4. Synthesis of an acyclic allylic alcohol nucleophile.

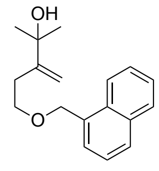

2-Methyl-3-methylene-5-(naphthalen-1-ylmethoxy)pentan-2-ol (36)

This compound was synthesized according to General Procedure B with the appropriate diol (150 mg, 1.15 mmol), NaH (55 mg, 1.4 mmol), and 1-(bromomethyl)naphthalene (280 mg, 1.27 mmol) in DMF (5.8 mL). The reaction was stirred for 2 h and then was purified by flash chromatography (20% Et_2O in hexanes) to yield **36** as an oil (252 mg, 81%).

^{1}H NMR: ($CDCl_3$, 300 MHz) δ 8.09 (dd, *J* = 1.7, 9.1 Hz, 1H), 7.86 (dd, *J* = 2.2, 6.6 Hz, 1H), 7.81 (d, *J* = 8.2 Hz, 1H), 7.56-7.39 (m, 4H), 5.08 (d, *J* = 0.5 Hz, 1H), 4.99 (s, 2H), 4.83 (d, *J* = 0.8 Hz, 1H), 3.71 (t, *J* = 6.3 Hz, 2H), 2.48 (td, *J* = 1.0, 6.4 Hz, 2H), 1.32 (s, 6H) ppm
^{13}C NMR: ($CDCl_3$, 125 MHz) δ 154.2, 133.9, 133.5, 131.8, 128.8, 128.7, 126.6, 126.4, 125.9, 125.3, 124.1, 109.5, 72.6, 71.8, 71.4, 31.9, 29.7 ppm
HRMS: (ESI), *m/z* calcd for $C_{18}H_{23}O_2$ $[M + H]^+$ 271.1693, found 271.1693
IR: (ATR, neat) 3413, 3048, 2973, 2864, 1639, 1511, 1363, 1164, 1042, 956, 859 cm^{-1}

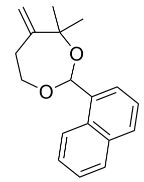

4,4-Dimethyl-5-methylene-2-(naphthalen-1-yl)-1,3-dioxepane (37)

This compound was synthesized according to General Procedure A with **36** (93.6 mg, 0.346 mmol), 4 Å MS (93.6 mg), and DDQ (118 mg, 0.519 mmol) in CH_2Cl_2 (3.5 mL). The oxidation step of the reaction was stirred for 18 h. $Sc(OTf)_3$ was added (17.2 mg, 0.035 mmol) and the reaction was stirred 1 h. The mixture was filtered through celite and cotton and concentrated to a residue. This residue purified by flash chromatography (20% hexanes in toluene) to afford **37** (31.1 mg, 33%) as a crystalline solid.

^{1}H NMR: ($CDCl_3$, 400 MHz) δ 8.13 (d, *J* = 8.4 Hz, 1H), 7.84-7.74 (m, 3H) 7.52-7.43 (m, 3H), 5.91 (s, 1H), 5.07 (s, 1H), 5.06 (s, 1H), 4.23 (dt, *J* = 3.6, 11.3, 1H) 3.74 (dt, *J* = 0.9, 11.6 Hz, 1H), 3.02 (td, *J* = 3.4, 12.7 Hz, 1H), 2.42 (dd, *J* = 2.0, 13.2, 1H), 1.46 (s, 3H), 1.44 (s, 3H) ppm
^{13}C NMR: ($CDCl_3$, 125 MHz) δ 155.6, 135.8, 133.9, 130.4, 128.9, 128.4, 125.9, 125.4, 125.0, 124.5, 123.7, 112.4, 79.5, 71.4, 37.4, 30.0, 25.6 ppm
HRMS: (ESI), *m/z* calcd for $C_{18}H_{21}O_2$ $[M+H]^+$ 269.1536, found 269.1532
IR: (ATR, neat) 2970, 2918, 1641, 1509, 1406, 1339, 1204, 1099, 1068, 1008, 904, 768 cm^{-1}
MP: 85.3 - 87.1 °C

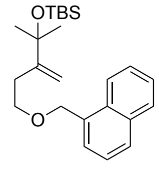

***tert*-Butyldimethyl((2-methyl-3-methylene-5-(naphthalen-1-ylmethoxy)pentan-2-yl)oxy)silane (38)**

TBSOTf (0.14 mL, 161 mg, 1.1 equiv, 0.61 mmol) was added dropwise to a stirring solution of **36** (150 mg, 1 equiv, 0.555 mmol) and 2,6-lutidine (0.13 mL, 119 mg, 2 equiv, 1.11 mmol) in CH_2Cl_2 (5.5 mL, 0.1 M). The mixture stirred for 1 h until no

more starting material remained by TLC. The reaction was quenched with saturated NH_4Cl (3 mL), then aqueous layer was extracted with CH_2Cl_2 (x3, 10 mL). The organic layers combined, dried over Na_2SO_4, filtered, and concentrated to clear oil. The crude oil was purified by flash chromatography (5% Et_2O in hexanes) to yield title compound (108 mg, 51%).

^{1}H NMR: ($CDCl_3$, 300 MHz) δ 8.13 (dd, *J* = 1.8, 9.1 Hz, 1H), 7.86 (dd, *J* = 2.2, 6.3 Hz, 1H), 7.80 (d, *J* = 8.2 Hz, 1H), 7.55-7.40 (m, 4H), 5.07 (d, *J* = 1.1 Hz, 1H), 4.98 (s, 2H), 4.75 (d, *J* = 1.2 Hz, 1H), 3.71 (t, *J* = 7.6 Hz, 2H), 2.47 (t, *J* = 7.3 Hz, 2H), 1.33 (s, 6H), 0.87 (s, 9H), 0.07 (s, 6H) ppm
^{13}C NMR: ($CDCl_3$, 75 MHz) δ 153.5, 134.2, 134.0, 132.0, 128.7, 126.5, 126.3, 125.9, 125.4, 124.3, 108.0, 77.2, 75.9, 71.6, 70.7, 31.2, 29.9. 26.1, 25.9, 18.4, –1.6, –2.8 ppm
HRMS: (ESI), *m/z* calcd for $C_{24}H_{37}O_2Si$ $[M+H]^+$ 385.2557, found 385.2548
IR: (ATR, neat) 3064, 2955, 2930, 2856, 1641, 1512, 1361, 1158, 1006, 937, 902, 832, cm^{-1}

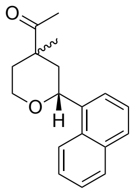

1-(4-Methyl-2-(naphthalen-1-yl)tetrahydro-2*H*-pyran-4-yl)ethan-1-one (39)
To a solution of **38** (100 mg, 1 equiv, 0.26 mmol) and 4 Å MS (100.0 mg) in a 1:1 mixture of CH_2Cl_2/MeCN (2.6 mL, 0.1 M), was added Bobbitt's salt (117 mg, 0.39 mmol) in a single portion. The reaction was stirred for 23 h before being filtered through cotton and concentrated to a residue. This residue was purified by flash chromatography (10% EtOAc in hexanes) to yield **39** as a mixture of diastereomers (56.5 mg, 81%). While these diastereomers were difficult to separate, small amounts could be isolated to deliver clean ^{1}H and ^{13}C NMR spectra. These samples were used for structural determination through NOESY analysis.

HRMS: (ESI), *m/z* calcd for $C_{18}H_{20}O_2$ $[M + H]^+$ 269.1542, found 269.1538
IR: (ATR, neat) 2963, 1702, 1261, 1092, 799 cm^{-1}

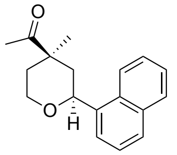

39

^{1}H NMR: ($CDCl_3$, 300 MHz) δ 7.99 (d, *J* = 8.36 Hz, 1H), 7.88 (m, 1H), 7.79 (d, *J* = 8.21 Hz, 1H), 7.66 (d, *J* = 7.16 Hz, 1H) 7.55-7.44 (m, 3H), 5.32 (dd, *J* = 2.9, 10.8 Hz, 1H), 4.22 (ddd, *J* =1.6, 5.4, 12.0 Hz, 1H), 3.99 (td, *J* = 2.3, 12.4, 1H), 2.15 (s, 3H), 2.11-2.01 (m, 1H), 2.01-1.87 (m, 2H), 1.61 (dq, *J* =2.1, 13.6 Hz, 1H), 1.53, (s, 3H) ppm
^{13}C NMR: ($CDCl_3$, 100 MHz) δ 212.2, 138.0, 133.8, 130.1, 129.0, 128.0, 126.0, 125.6, 125.4, 123.1, 122.7, 71.9, 64.3, 46.1, 40.0, 32.4, 24.3, 19.3 ppm

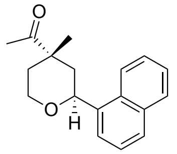

39'

^{1}H NMR: ($CDCl_3$, 300 MHz) δ 8.25 (d, *J* = 8.6, 1H), 7.85 (d, *J* = 8.2, 1H), 7.76 (d, *J* = 8.2 Hz, 1H), 7.64 (d, *J* = 7.2 Hz, 1H) 7.56 (m, 1H), 7.61-7.52 (m, 2H), 5.12 (dd, *J* = 1.7, 11.7 Hz, 1H) 4.18 (ddd, *J* = 1.6, 4.8, 11.9 Hz, 1H), 3.64 (ddd, *J* = 2.1, 12.1, 13.0 Hz, 1H), 2.63 (dt, *J* = 2.3, 13.9 Hz, 1H). 2.29 (s, 3H), 2.22 (dq, *J* = 2.1, 14.2, 1H), 1.80 (ddd, *J* = 4.75, 13.2, 14.0 Hz, 1H), 1.43 (m, 1H), 1.18 (s, 3H) ppm
^{13}C NMR: ($CDCl_3$, 100 MHz) δ 213.1, 139.1, 133.8, 130.5, 128.9, 127.9, 126.3, 125.7, 125.6, 123.6, 122.4, 66.4, 48.3, 42.5, 35.7, 27.4, 25.5 ppm

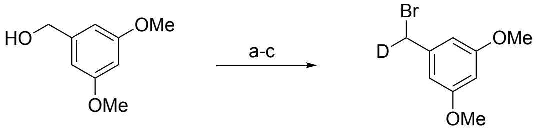

Reagents and conditions
a) Dess-Martin periodinane, $NaHCO_3$, CH_2Cl_2, 75%. b) $NaBD_4$, CH_2Cl_2, 0 °C to rt, 37%. c) PBr_3, Et_2O, 0 °C, 68%.

Scheme S5. Synthesis of deuterated bromide **S4**.

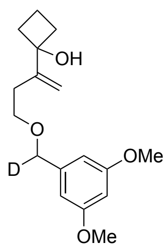

1-(4-((3,5-Dimethoxyphenyl)methoxy-*d*)but-1-en-2-yl)cyclobutan-1-ol (25-D1)
This compound was synthesized according to General Procedure **B** with **S3** (35.6 mg, 0.250 mmol), NaH (12.0 mg, 0.30 mmol), and **S4** (63.8 mg, 0.275 mmol) in THF (0.8 ml). The reaction was stirred for 19 h and then was purified by flash chromatography (20% EtOAc in hexanes) to afford **25-D1** (21.7 mg, 30%) as a light brown oil.

^{1}H NMR: ($CDCl_3$, 300 MHz) δ 6.47 (d, J = 2.2 Hz, 2H), 6.38 (t, J =2.3 Hz, 1H) 5.17 (d, J = 0.9 Hz, 1H), 4.96 (d, J = 0.9 Hz, 1H), 4.45 (s, 1H), 3.78 (s, 6H), 3.64 (s, 1H), 3.61 (t, J = 6.0 Hz, 2H), 2.42 (td, J = 0.9, 6.9 Hz, 2H), 2.33-2.22 (m, 2H), 2.17-2.04 (m, 2H), 1.91-1.76 (m, 1H), 1.55-1.44 (m, 1H)
^{13}C NMR: ($CDCl_3$, 100 MHz) δ 160.9, 149.9, 140.0, 110.8, 105.4, 99.9, 77.2, 72.8 (t, J = 21.9), 70.9, 55.3, 35.0, 31.7, 29.7, 12.8 ppm
HRMS: *m/z* cald for $C_{17}H_{22}{}^2HO_3$ $[M\text{-}OH]^+$ 276.1705, found 276.1715
IR: (ATR, neat) 3412, 2935, 2860, 1597, 1428, 1294, 1205, 1095, 902 cm^{-1}

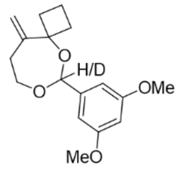

Kinetic isotope effect study
To a mixture of **25-D1** (19.9 mg, .0678 mmol) in CH_2Cl_2 (0.68 mL, .1 M) were added 4 Å MS (19.9 mg) and DDQ (23.1 mg, 0.102 mmol). The mixture was stirred for 4 h then was filtered through Celite and cotton and concentrated to a residue. This residue was purified by flash chromatography (20% EtOAc in hexanes) to yield an 8.1:1 mixture of **40-D** and **40-H** (6.5 mg, 33%). This value was calculated based on the integration of the acetal hydrogen to 0.11H, leading to the assumption that the remaining 0.89H at this center is D.

^{1}H NMR: ($CDCl_3$, 400 MHz) δ 6.66 (d, J = 2.3 Hz, 2H), 6.41 (t, J = 2.34 Hz, 1H), 5.29 (s, 0.1H), 5.22 (s, 1H), 5.09 (s, 1H), 4.20 (dt, J = 3.6, 11.3 Hz, 1H), 3.80 (s, 6H), 3.57 (td, J = 1.8, 11.6 Hz, 1H), 2.51 (qt, J = 4.1, 8.4 Hz, 1H), 2.42 (dt, J = 9.7, 10.6 Hz, 1H), 2.34 (ddt, J = 1.5, 2.0, 13.3, 1H), 2.25 (dt, J = 9.6, 11.1 Hz, 1H), 2.10-2.03 (m, 1H), 1.94-1.84 (m, 1H), 1.74-1.63 (m, 1H) ppm
^{13}C NMR: ($CDCl_3$, 100 MHz) δ 160.7, 152.1, 142.5, 111.8, 104.0, 110.5, 82.9, 71.0, 55.4, 36.9, 36.5, 33.0, 13.0 ppm
HRMS: *m/z* calcd for $C_{17}H_{22}{}^2HO_4$ $[M + H]^+$ 292.1654, found 292.1648
IR: (ATR, neat) 2963, 1600, 1461, 1430, 1261, 1153, 1028, 800 cm^{-1}

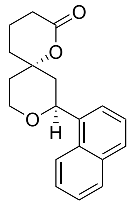

8-(Naphthalen-1-yl)-1,9-dioxaspiro[5.5]undecan-2-one (41)

To a stirring solution of **41** (70 mg, 0.25 mmol) in toluene (5 ml, 0.05 M) was added Li_2CO_3 (370 mg, 5.0 mmol) and *m*CPBA (284 mmol, 1.5 mmol) (*CAUTION m*CPBA) is an oxidant and can cause skin and eye damage. Avoid skin contact and wear eye protection). The mixture was heated to 80 °C using an oil bath and stirred for 22 h. The reaction was quenched with saturated sodium thiosulfate (10 ml) and extracted with CH_2Cl_2 (3 x 5 ml). The combined organic layers were washed with brine (10 ml), dried over Na_2SO_4, and concentrated to a residue. This residue was purified by flash chromatography (30% EtOAc in hexanes) to afford **41** as an off-white solid (42.6 mg, 58%).

^{1}H NMR: ($CDCl_3$, 500 MHz) δ 8.05 (d, *J* = 8.4 Hz, 1H), 7.85 (d, *J* = 7.6 Hz, 1H), 7.78 (d, *J* = 8.2 Hz, 1H), 7.64, (d, *J* = 7.1 Hz, 1H), 7.52 (td, *J* = 1.3, 6.8 Hz, 1H), 7.27 (m, 2H), 5.62 (dd, *J* = 1.7, 11.4 Hz, 1H), 4.26 (td, *J* = 3.1, 11.8 Hz, 1H), 4.15 (ddd, *J* = 1.7, 4.7, 11.7 Hz, 1H), 2.59 (td, *J* = 2.9, 6.7 Hz, 2H), 2.27 (td, *J* = 2.1, 14.1 Hz, 1H), 1.98-1.87 (m, 4H), 1.86-1.72 (m, 3H) ppm
^{13}C NMR: ($CDCl_3$, 125 MHz) δ 170.7, 137.9, 133.8, 130.2, 128.9, 128.2, 126.4, 125.7, 125.6, 123.2, 122.9, 80.9, 71.5, 63.8, 44.6, 37.2, 34.4, 29.7, 15.8 ppm
HRMS: (ESI), *m/z* calcd for $C_{19}H_{21}O_3$ $[M + H]^+$ 297.1485, found 297.1471
IR: (ATR, neat) 3052, 2952, 1726, 1597, 1422, 1263, 1079, 992, 899 cm^{-1}

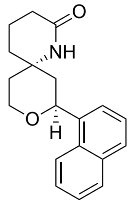

8-(Naphthalen-1-yl)-9-oxa-1-azaspiro[5.5]undecan-2-one (42)

Compound **27** (100 mg, 1 equiv, 0.358 mmol) was added to a solution of hydroxylamine hydrochloride (248 mg, 10 equiv, 3.58 mmol) (*CAUTION* hydroxylamine hydrochloride is a corrosive and orally toxic skin sensitizer. Avoid contact with skin and do not ingest) and $NaHCO_3$ (300 mg, 10 equiv, 3.58 mmol) in EtOH/H_2O (15:1, 4.8 mL, 0.075 M). This mixture was heated to reflux and stirred for 20 h until no more starting material was observed via ^{1}H NMR. Upon cooling to rt, CH_2Cl_2 (5 mL) was added to the mixture. The aqueous layer was then extracted with CH_2Cl_2 (5 mL, 3x). The organic layers were combined, dried over Na_2SO_4, filtered, and concentrated. This residue was dissolved in hexafluoroisopropanol (HFIP) (0.71 mL), then Re_2O_7 (5.7 mg, 0.05 equiv, 0.018 mmol) was added and heated to 60 °C for 4 h (*CAUTION* HFIP can cause burns and eye damage. Avoid skin contact and wear eye protection). The mixture was cooled to rt, then was filtered through a silica gel plug with CH_2Cl_2 and EtOAc to yield the title product (86 mg, 82%).

^{1}H NMR: ($CDCl_3$, 300 MHz) δ 8.09 (d, *J* = 9.4 Hz, 1H), 7.86 (d, *J* = 9.5 Hz, 1H), 7.77 (d, *J* = 8.1 Hz, 1H), 7.71 (d, *J* = 7.1 Hz, 1H), 7.47 (m, 3H), 5.91 (dd, *J* = 1.8, 11.4 Hz, 1H), 4.34 (td, *J* = 3.9, 11.4 Hz, 1H), 4.15 (ddd, *J* = 1.8, 4.7, 11.7 Hz, 1H), 2.68 (t, *J* = 7.5 Hz, 2H), 2.15 (d, *J* = 14.1 Hz, 1H), 1.90-1.57 (m, 7H) ppm
^{13}C NMR: ($CDCl_3$, 125 MHz) δ 171.0, 139.5, 133.8, 130.4, 128.9, 127.6, 126.0, 125.8, 125.4, 123.6, 122.5, 72.5, 65.0, 44.1, 42.6, 42.2, 34.5, 27.2, 19.9 ppm
HRMS: (ESI), *m/z* calcd for $C_{19}H_{22}O_2N$ $[M + H]^+$ 296.1645, found 296.1641
IR: (ATR, neat) 2946, 2834, 1653, 1450, 1113, 1020 cm^{-1}

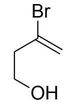

S1, ^{1}H NMR
$CDCl_3$, 500 MHz

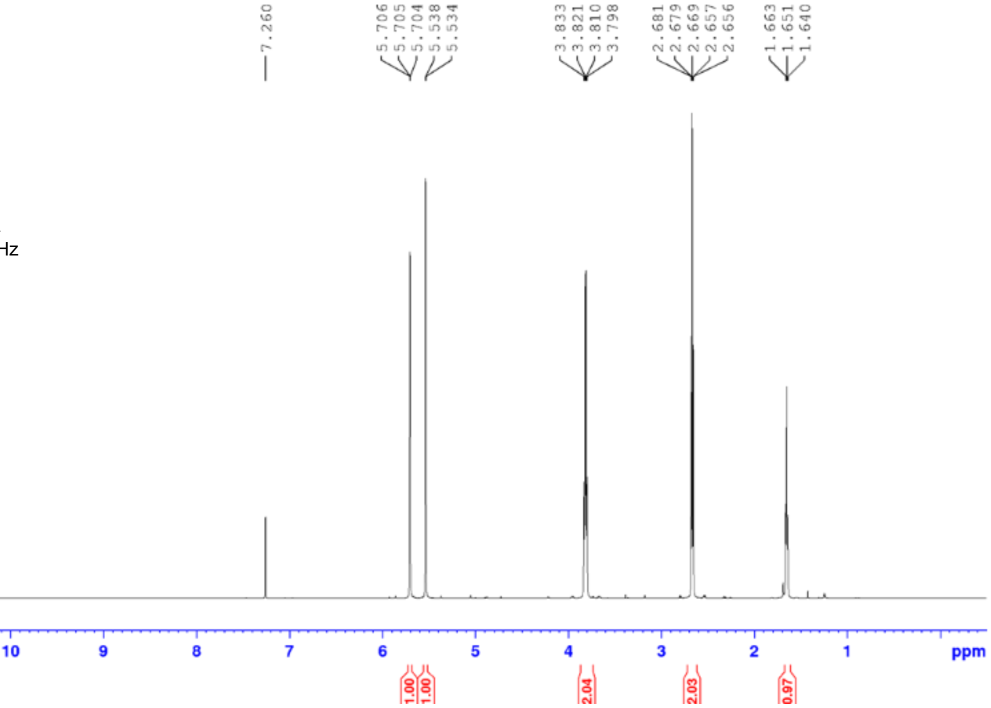

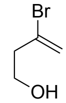

S1, ^{13}C NMR
$CDCl_3$, 125 MHz

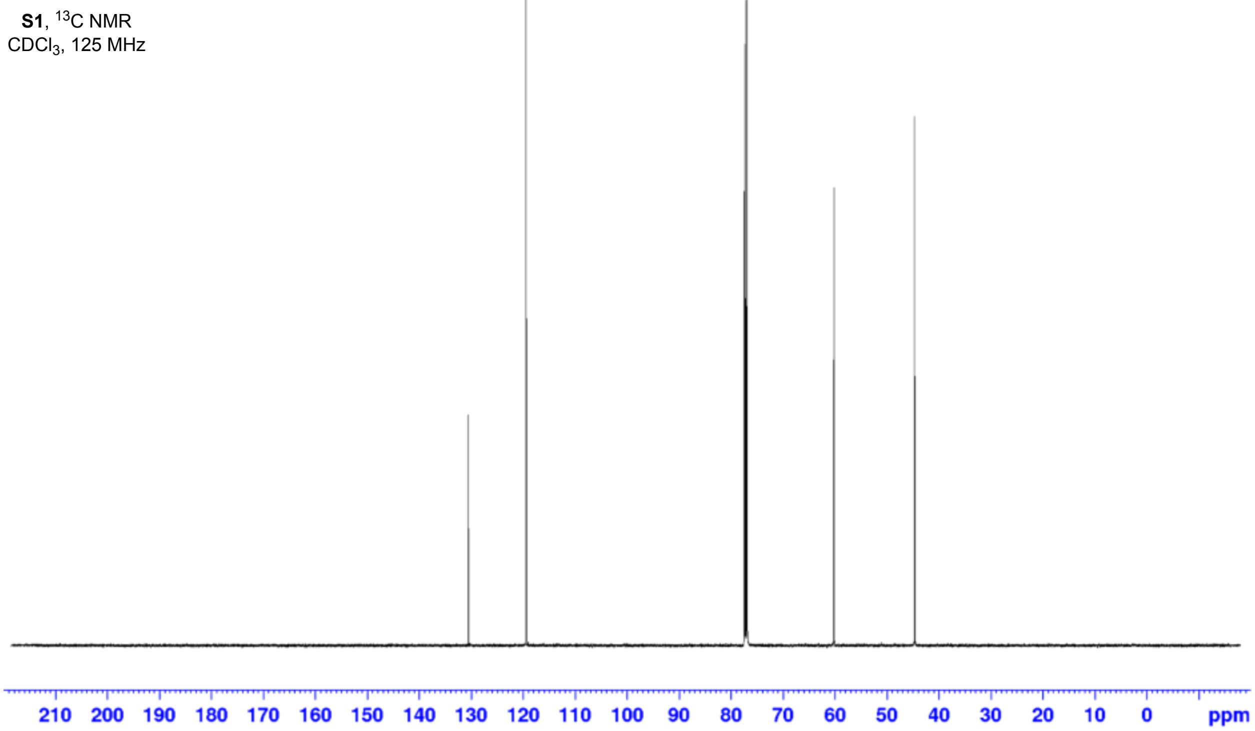

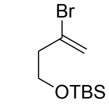

S2, ^{1}H NMR
$CDCl_3$, 400 MHz

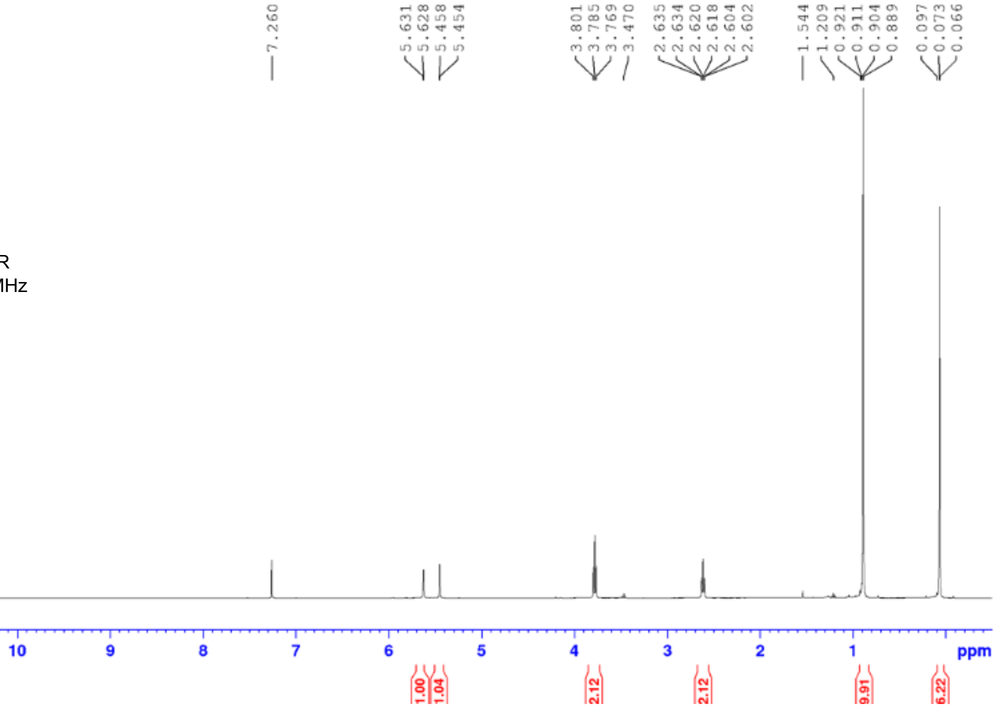

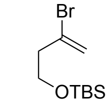

S2, ^{13}C NMR
$CDCl_3$, 100 MHz

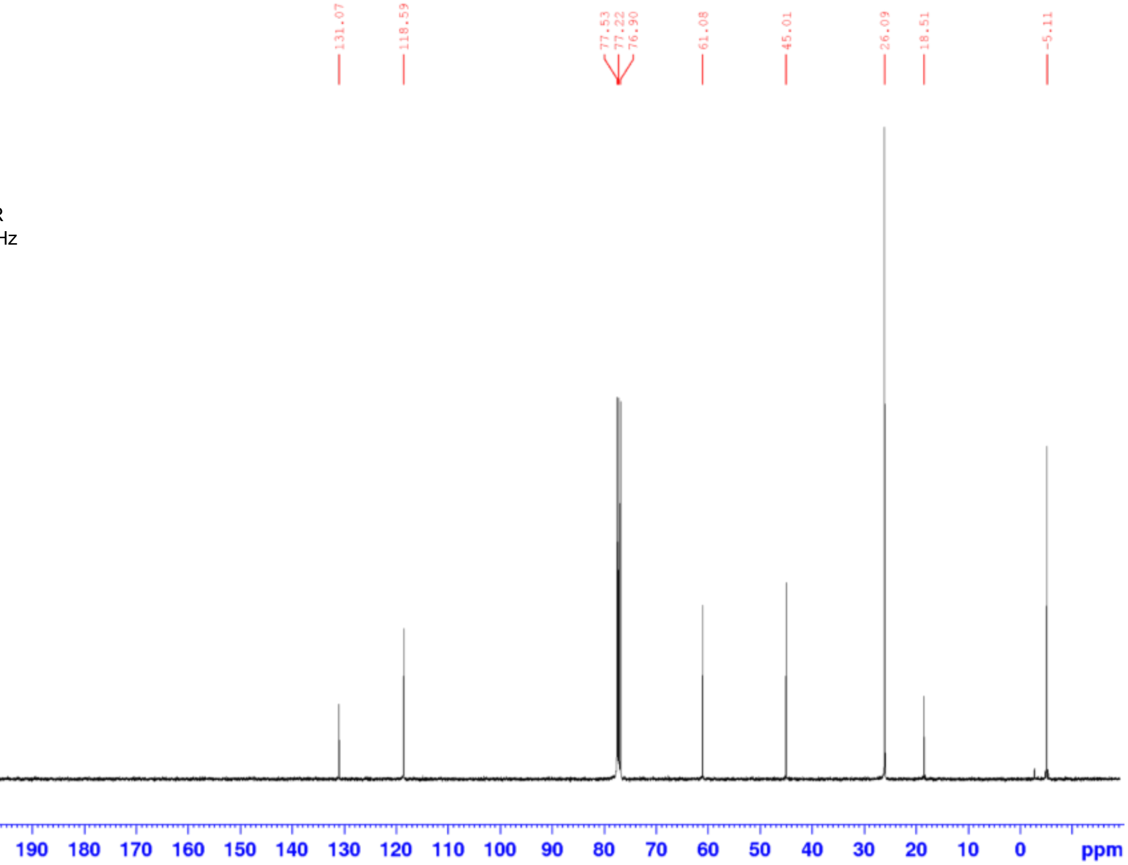

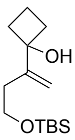

9, ^{1}H NMR
$CDCl_3$, 400 MHz

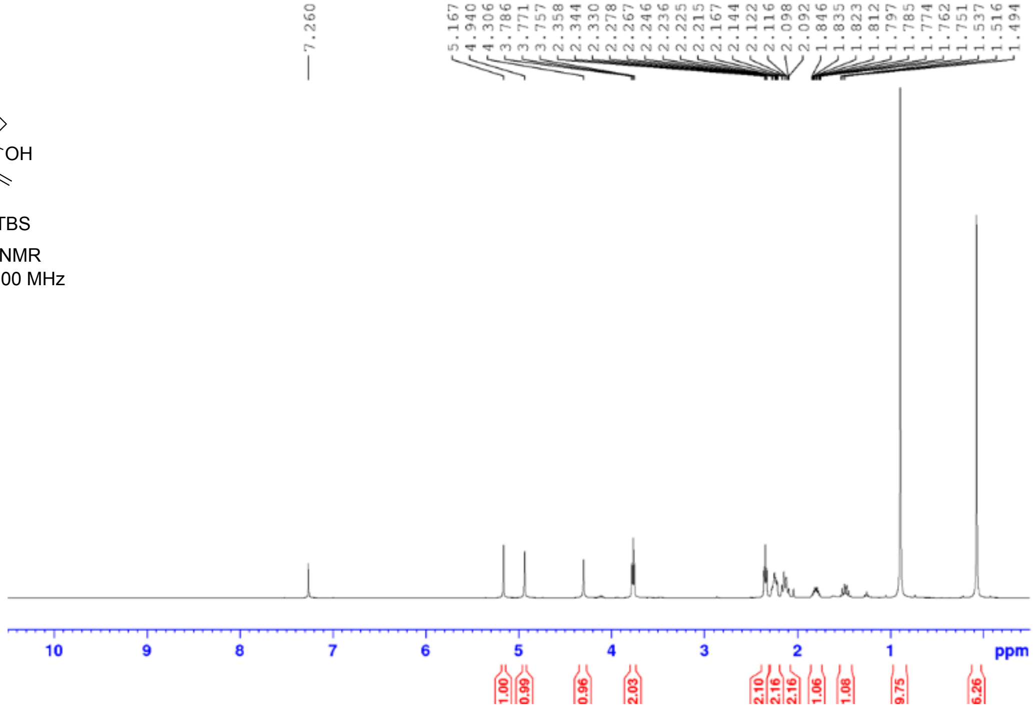

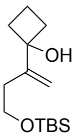

9, ^{13}C NMR
$CDCl_3$, 100 MHz

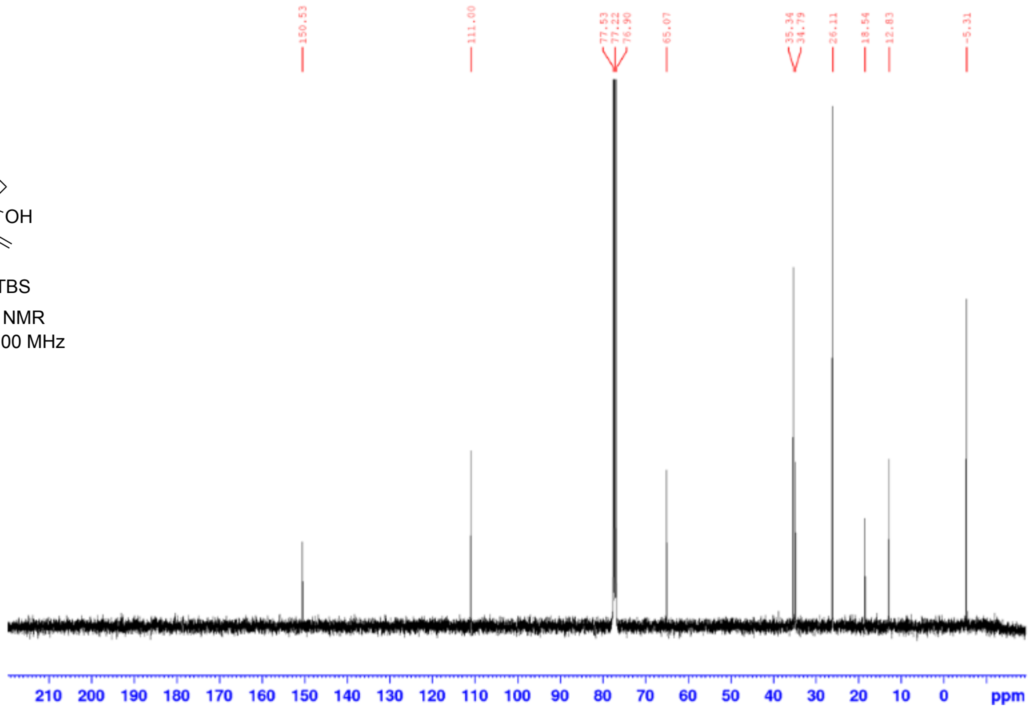

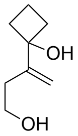

S3, ^{1}H NMR
$CDCl_3$, 300 MHz

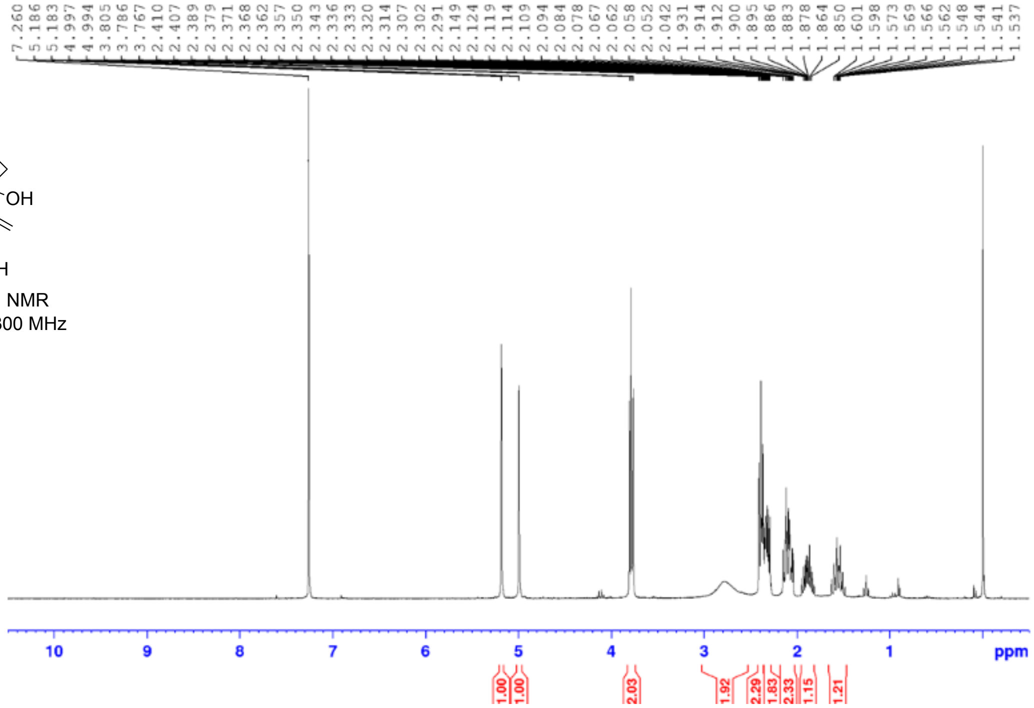

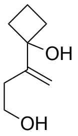

S3, ^{13}C NMR
$CDCl_3$, 125 MHz

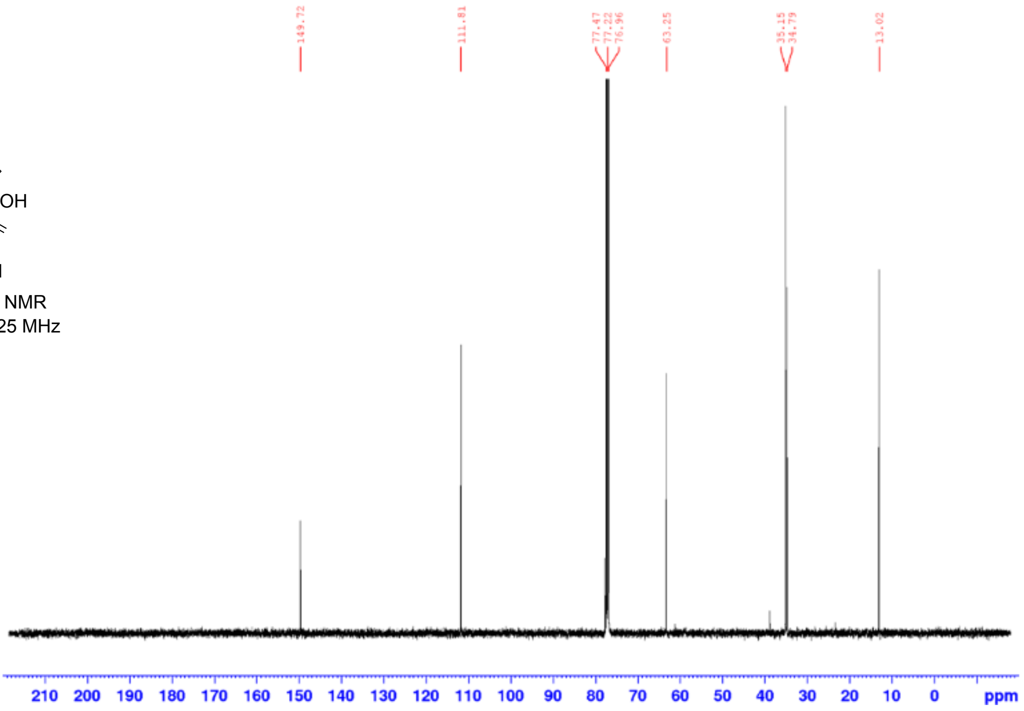

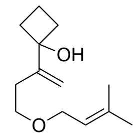

10, ^{1}H NMR
$CDCl_3$, 300 MHz

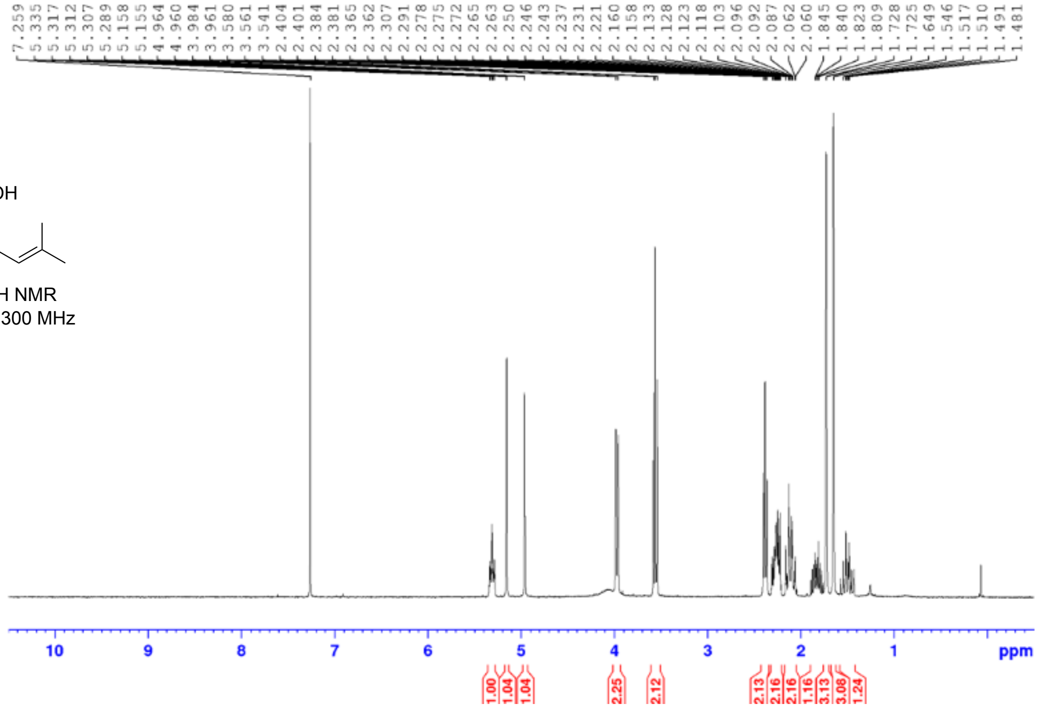

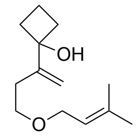

10, ^{13}C NMR
$CDCl_3$, 100 MHz

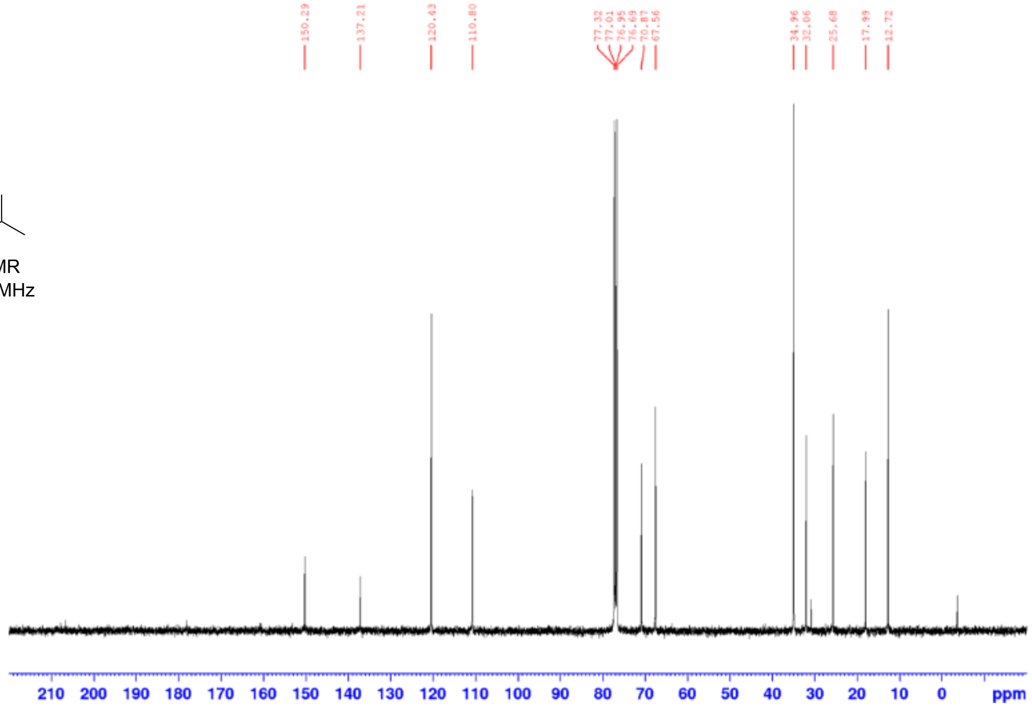

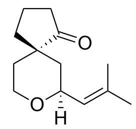

12, ^{1}H NMR
$CDCl_3$, 300 MHz

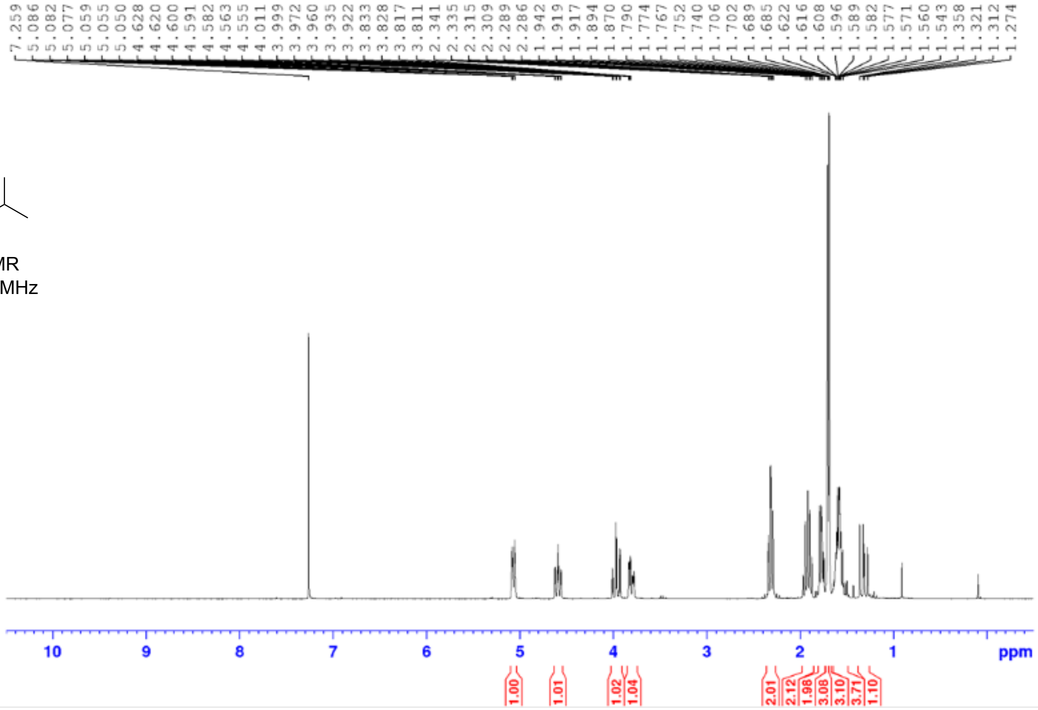

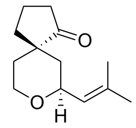

12, ^{13}C NMR
$CDCl_3$, 100 MHz

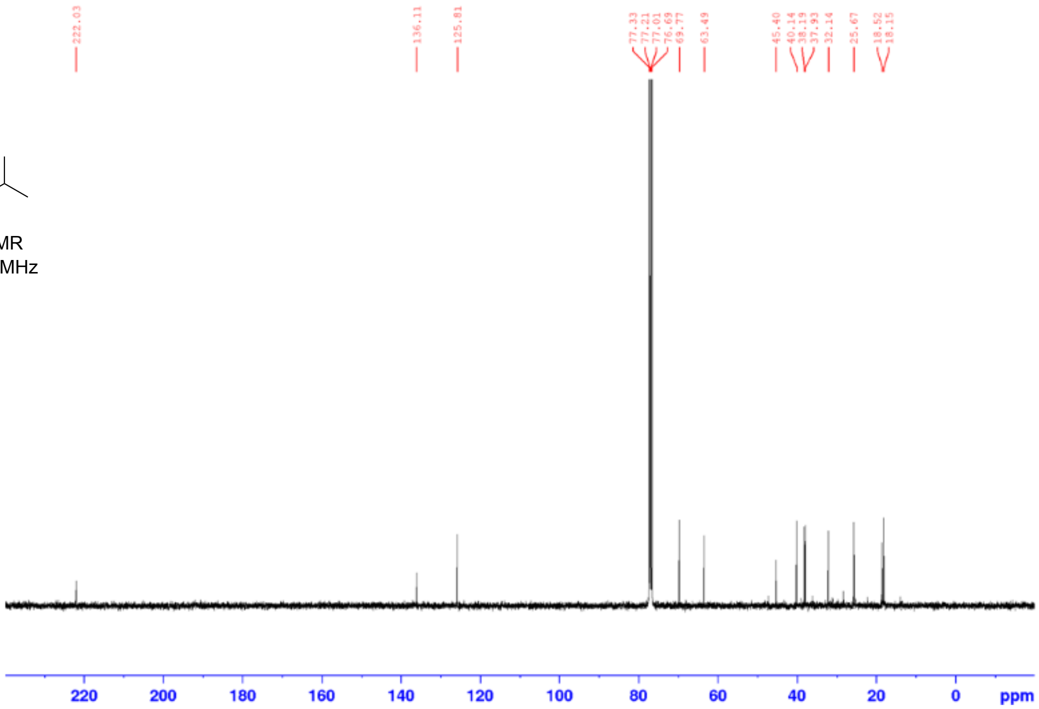

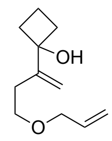

14, ^{1}H NMR
CDCl$_3$, 400 MHz

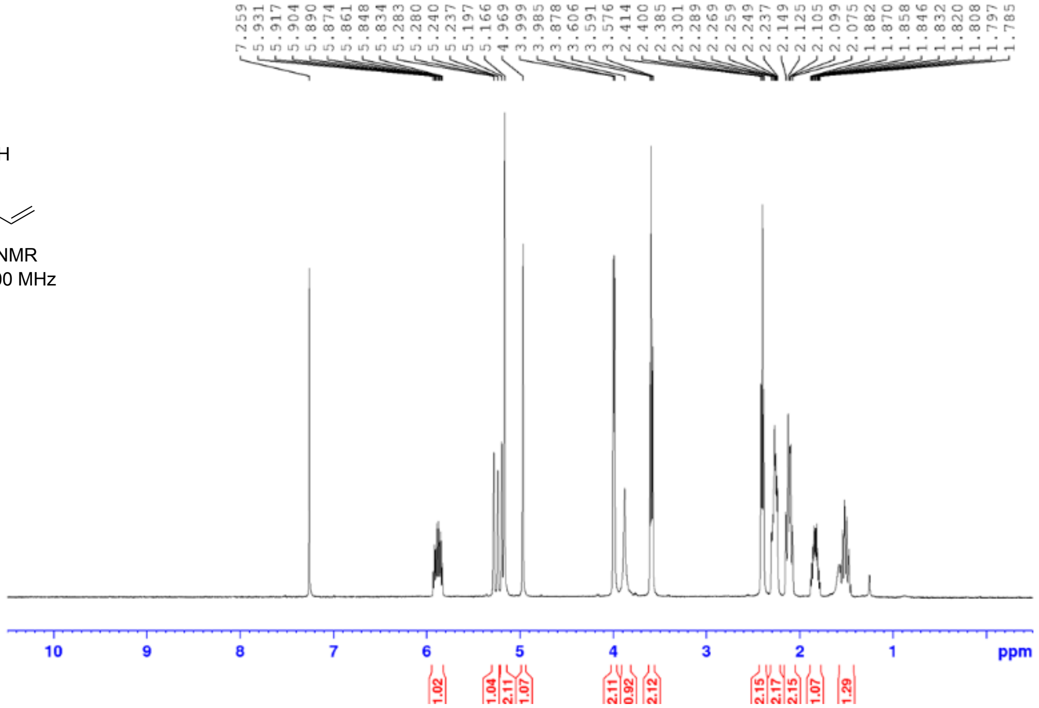

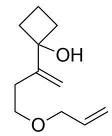

14, ^{13}C NMR
CDCl$_3$, 125 MHz

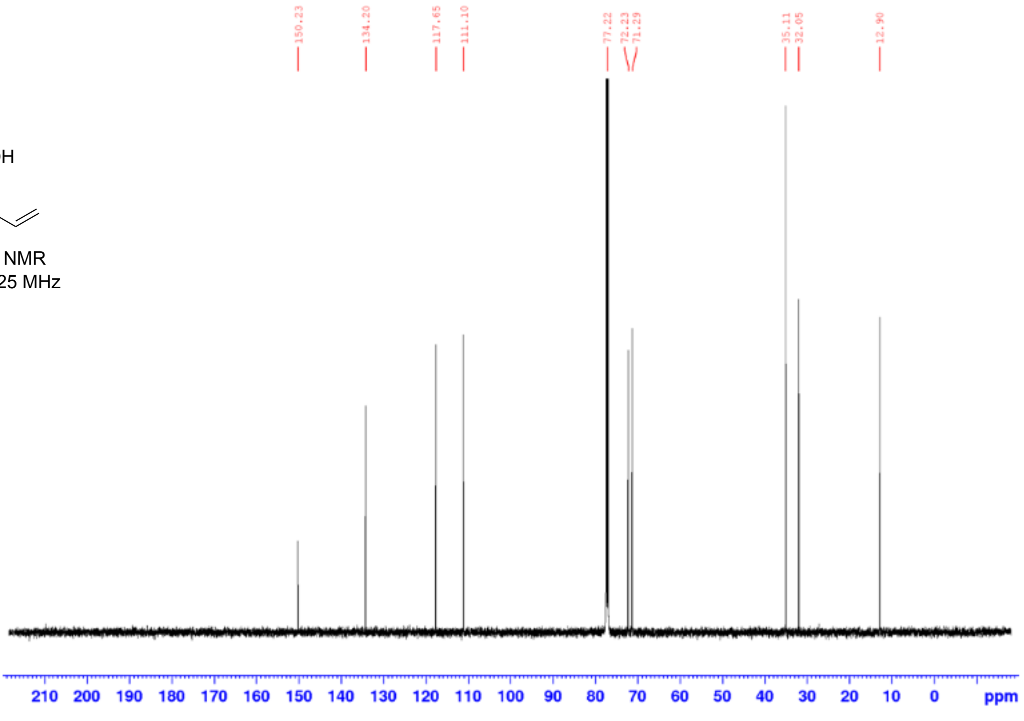

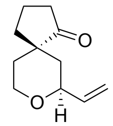

16, ^{1}H NMR
$CDCl_3$, 300 MHz

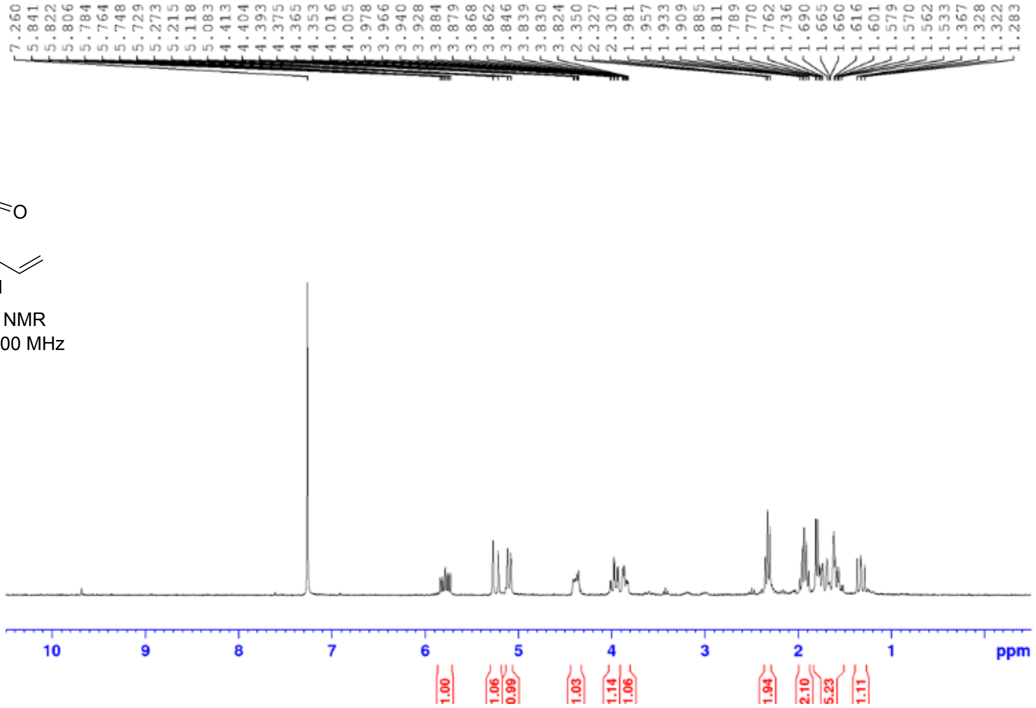

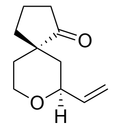

16, ^{13}C NMR
$CDCl_3$, 100 MHz

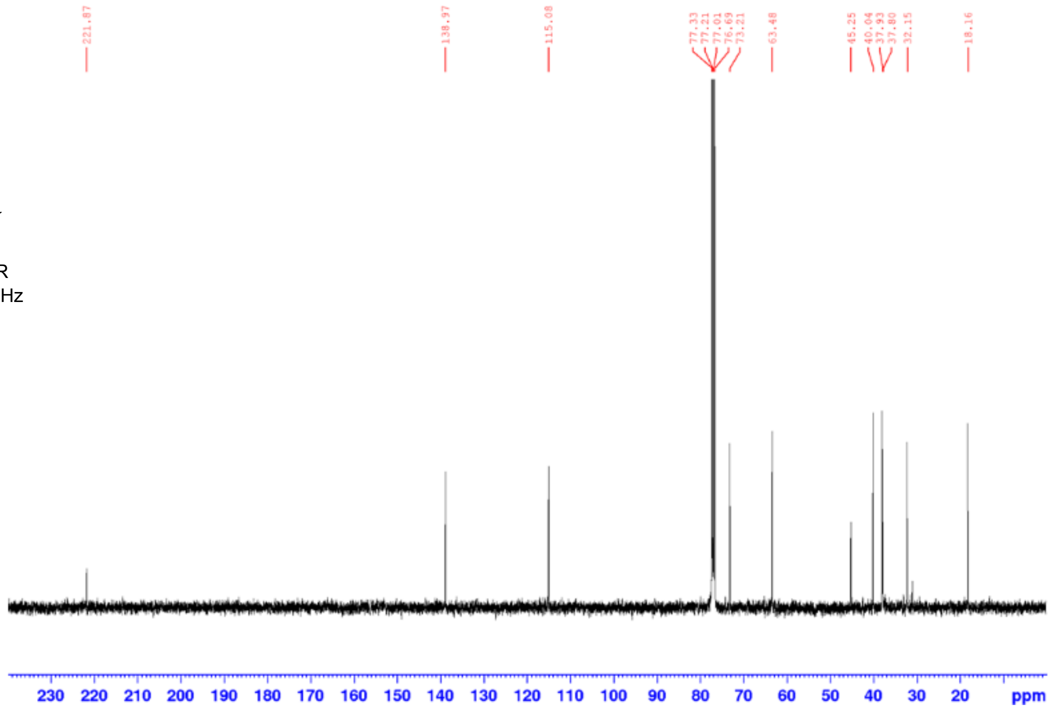

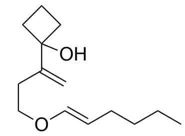

17, ^{1}H NMR
$CDCl_3$, 400 MHz

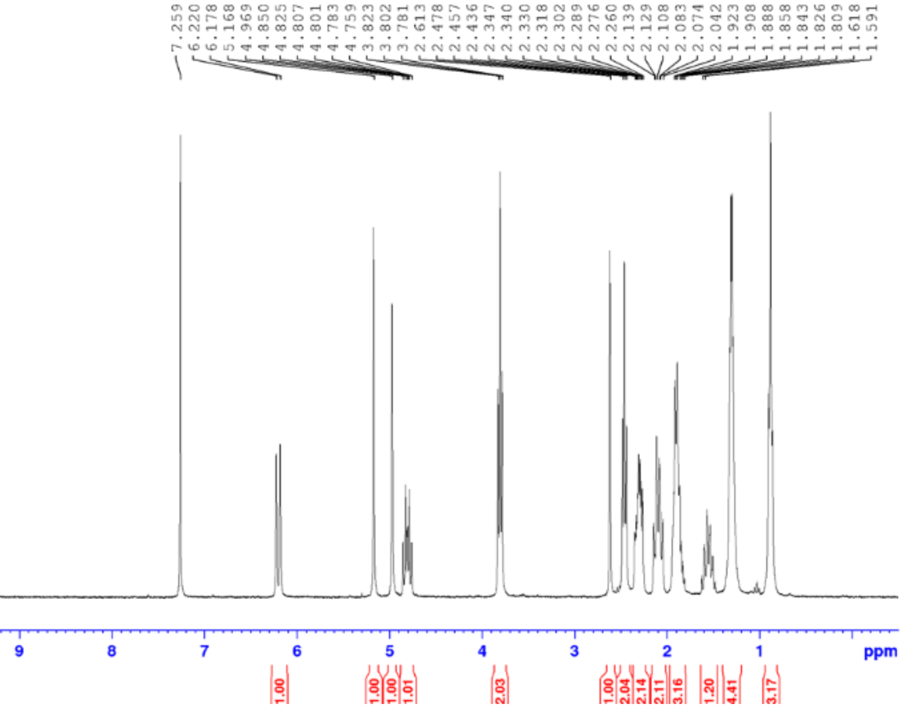

17, ^{13}C NMR
$CDCl_3$, 100 MHz

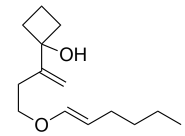

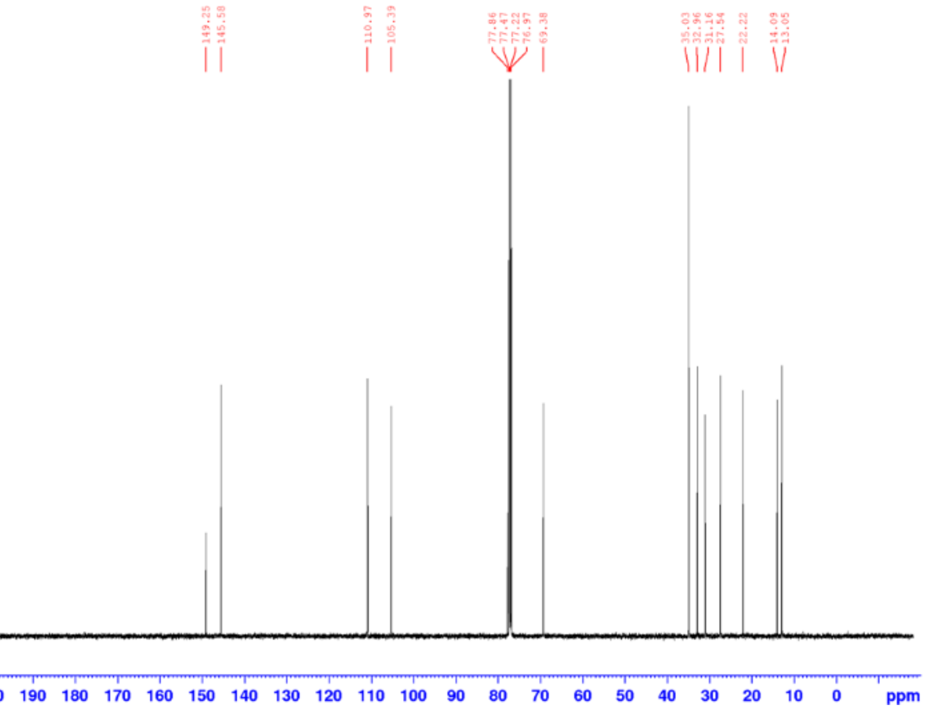

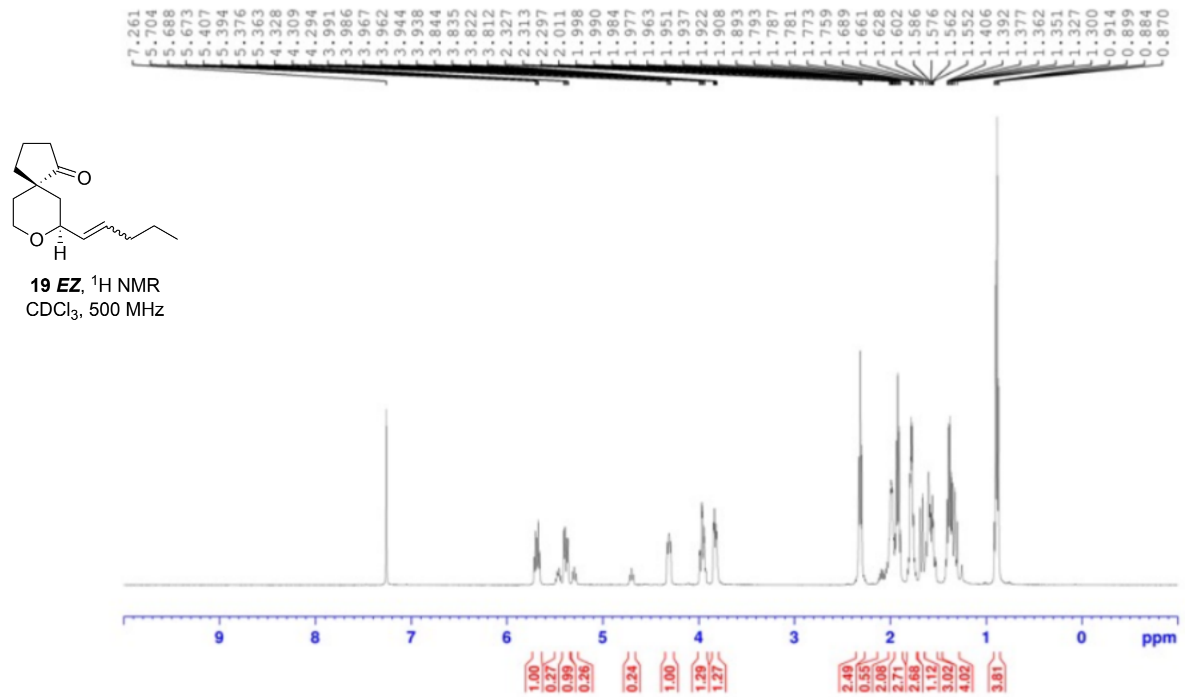

19 ***EZ***, ^{1}H NMR
$CDCl_3$, 500 MHz

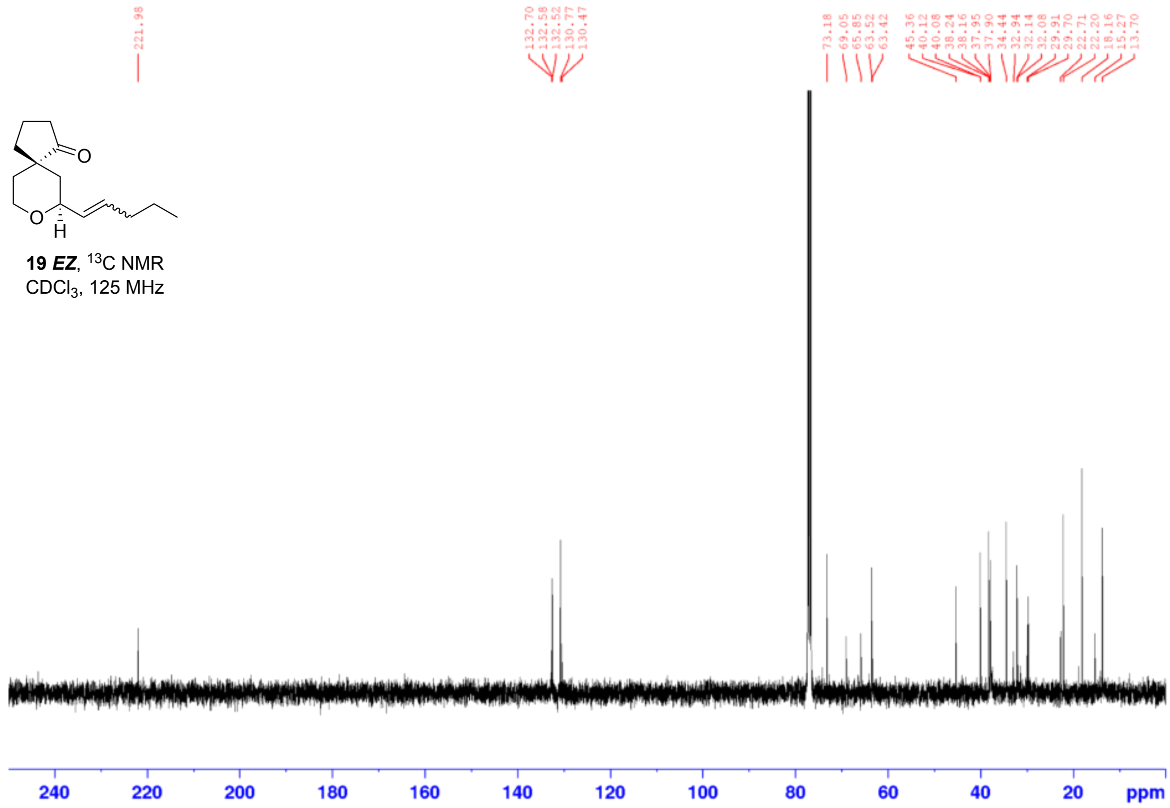

19 ***EZ***, ^{13}C NMR
$CDCl_3$, 125 MHz

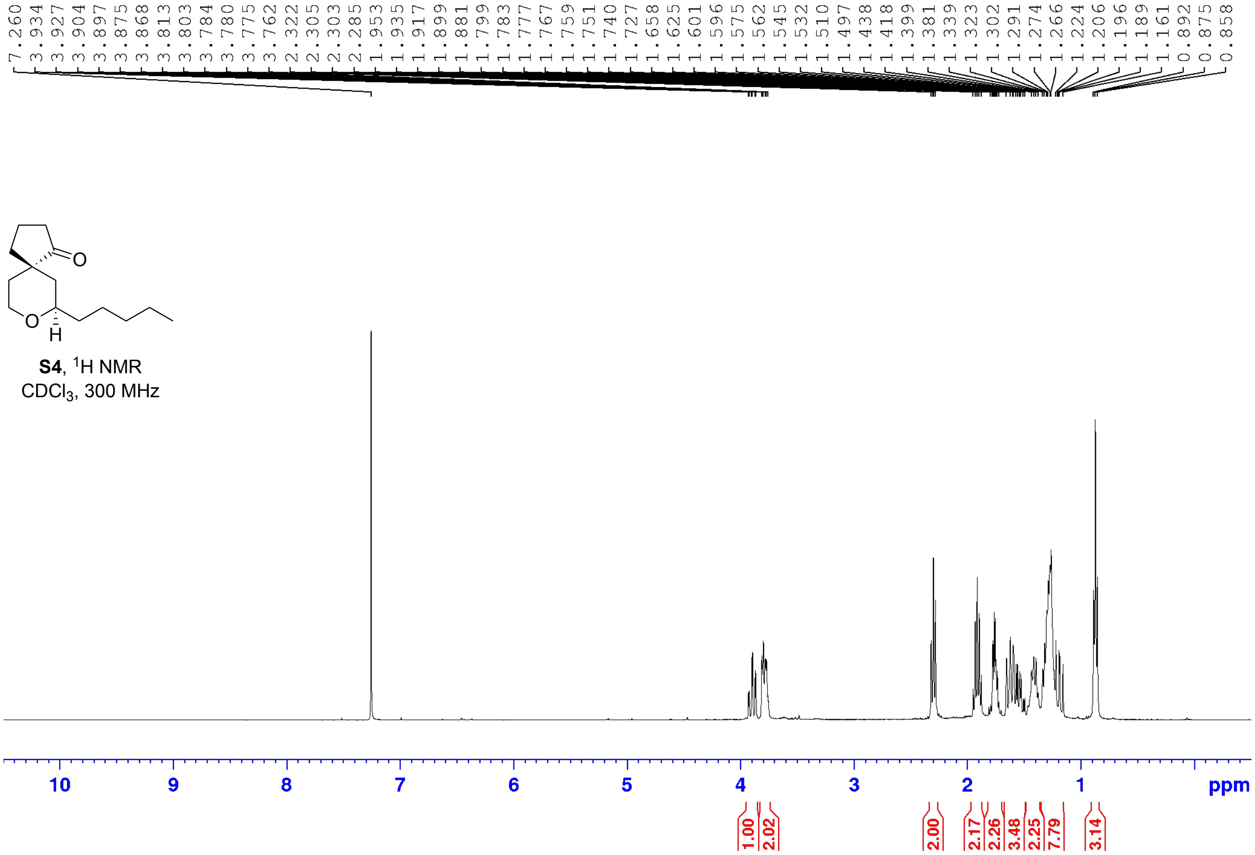

S4, ^{1}H NMR
$CDCl_3$, 300 MHz

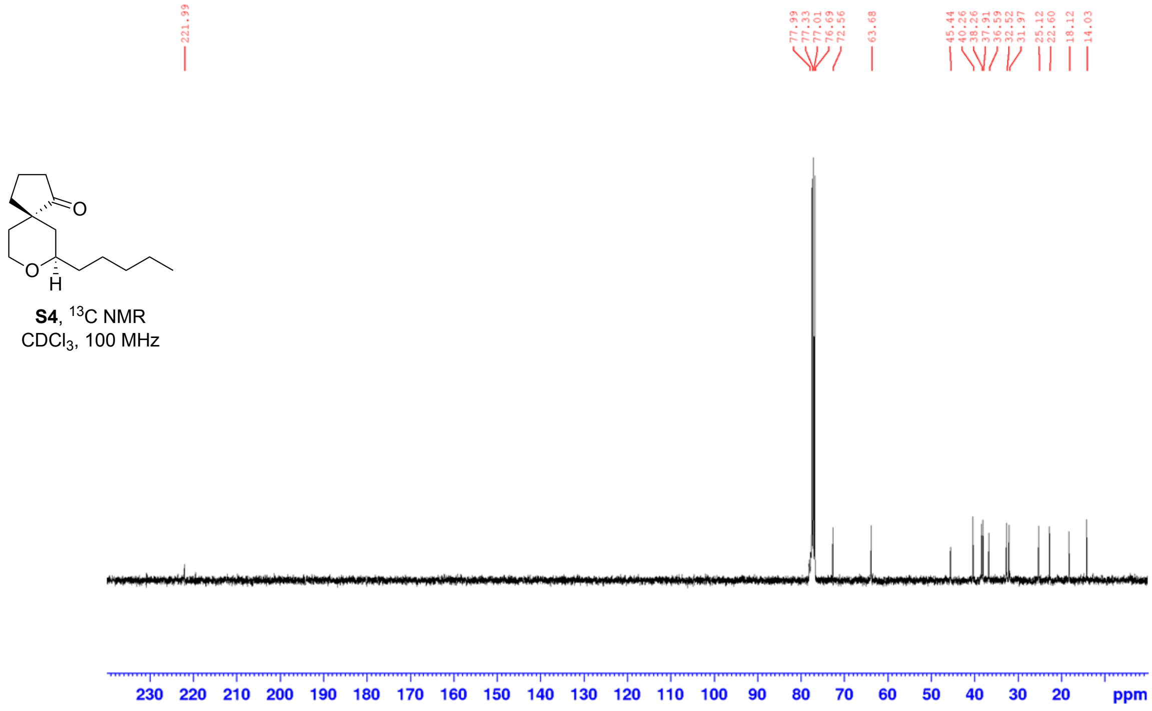

S4, ^{13}C NMR
$CDCl_3$, 100 MHz

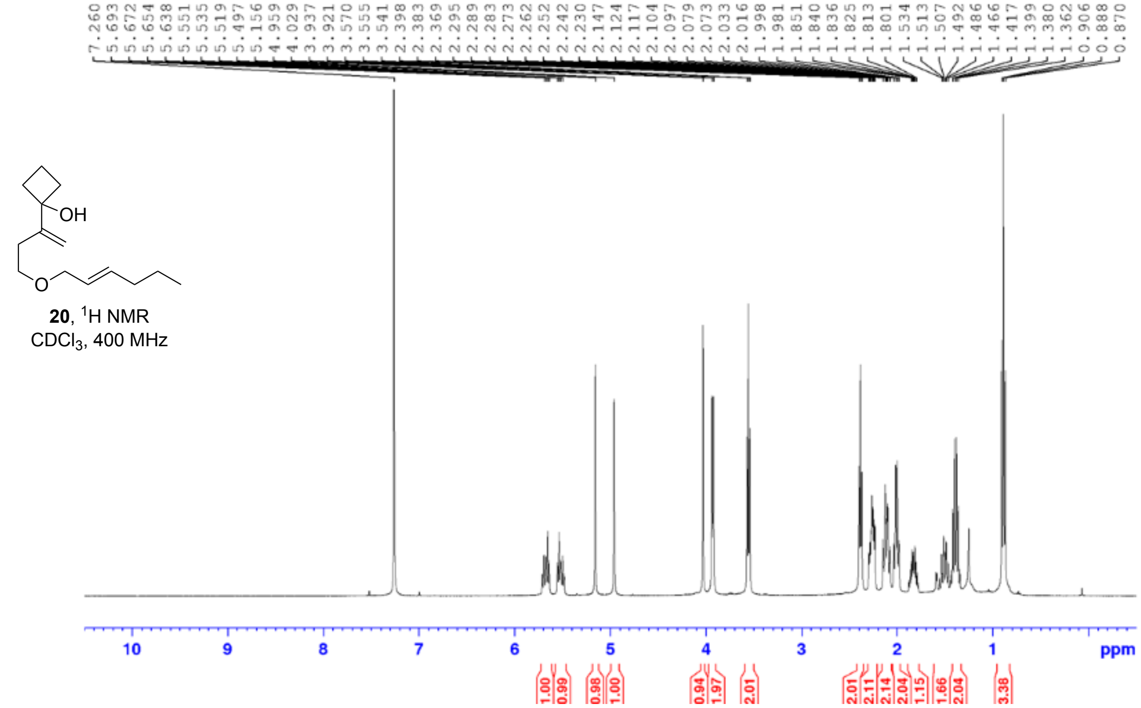

20, ^{1}H NMR
$CDCl_3$, 400 MHz

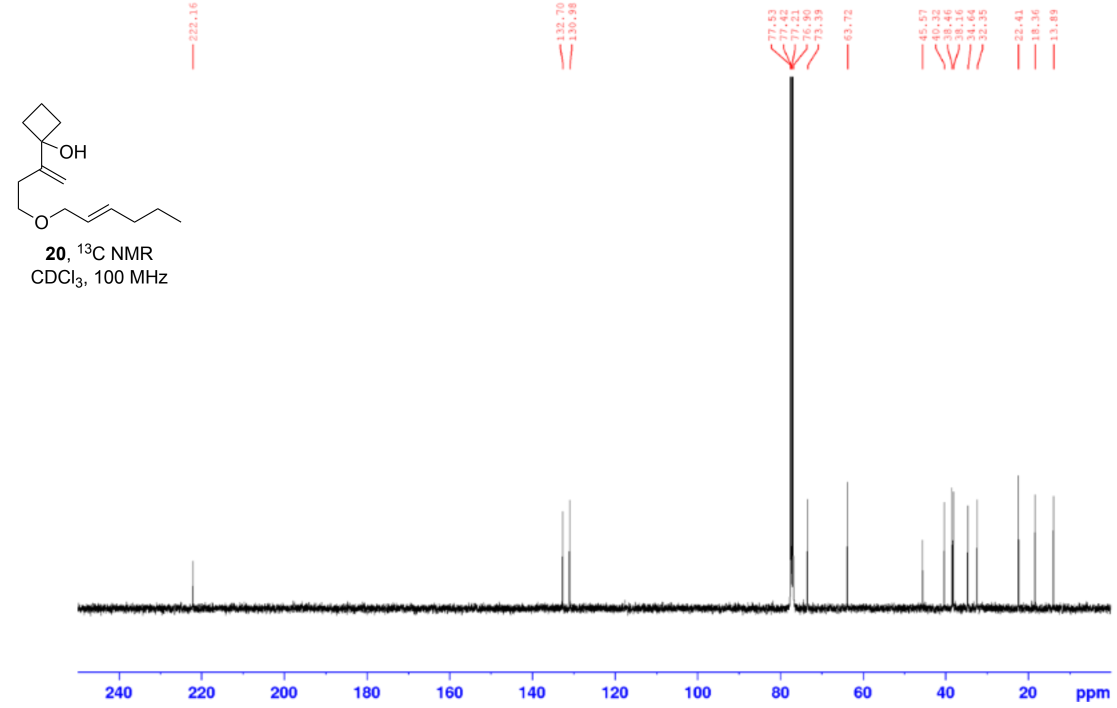

20, ^{13}C NMR
$CDCl_3$, 100 MHz

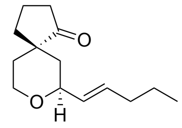

19 *E*, ^{1}H NMR
$CDCl_3$, 400 MHz

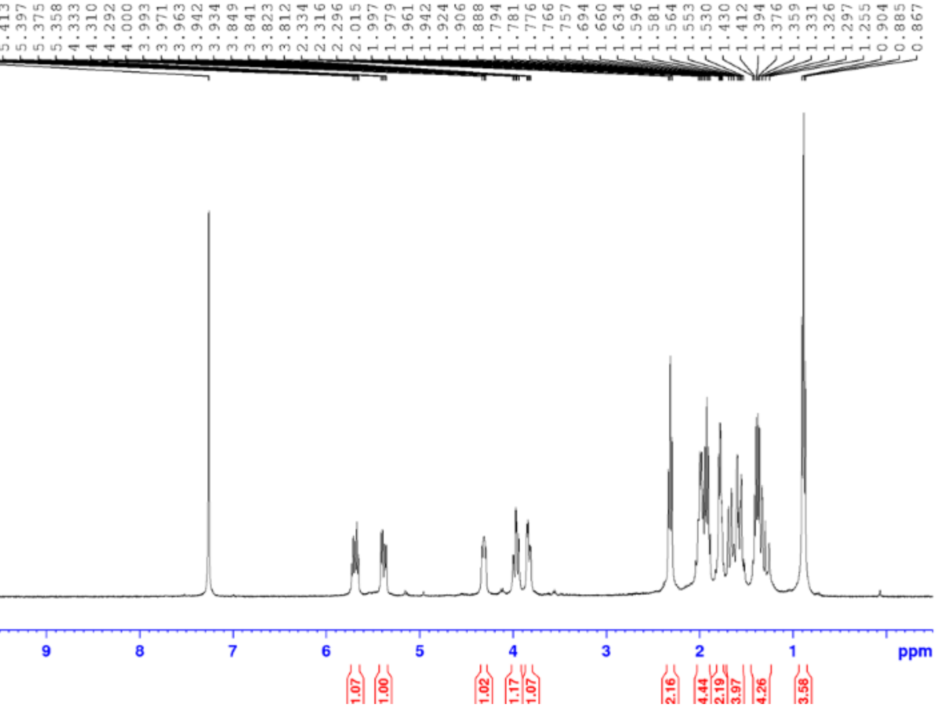

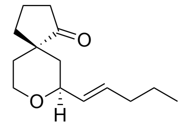

19 *E*, ^{13}C NMR
$CDCl_3$, 100 MHz

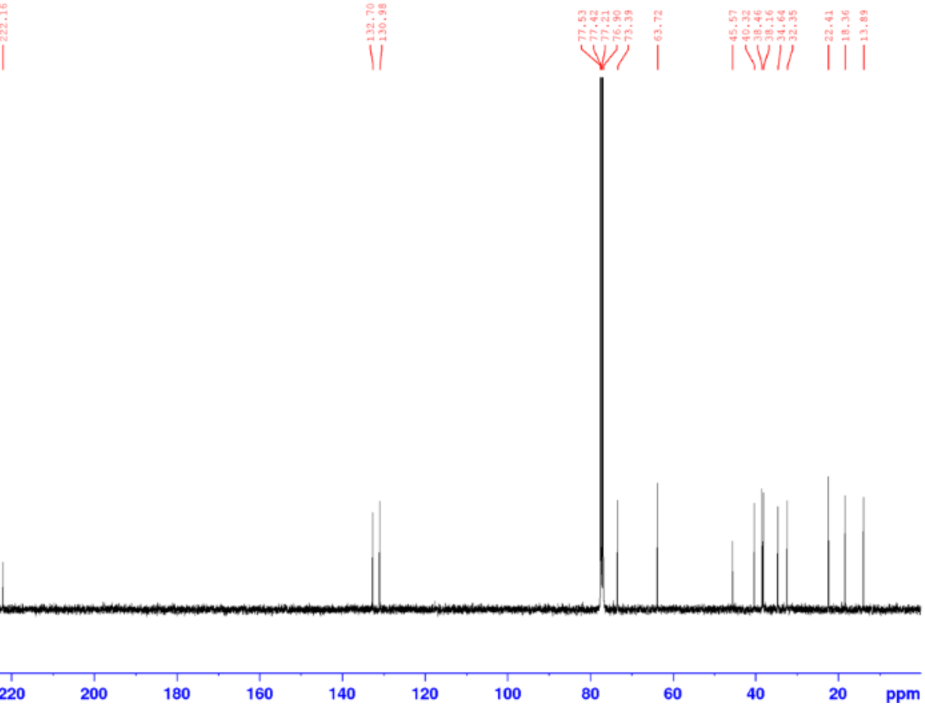

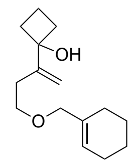

21, ^{1}H NMR
$CDCl_3$, 400 MHz

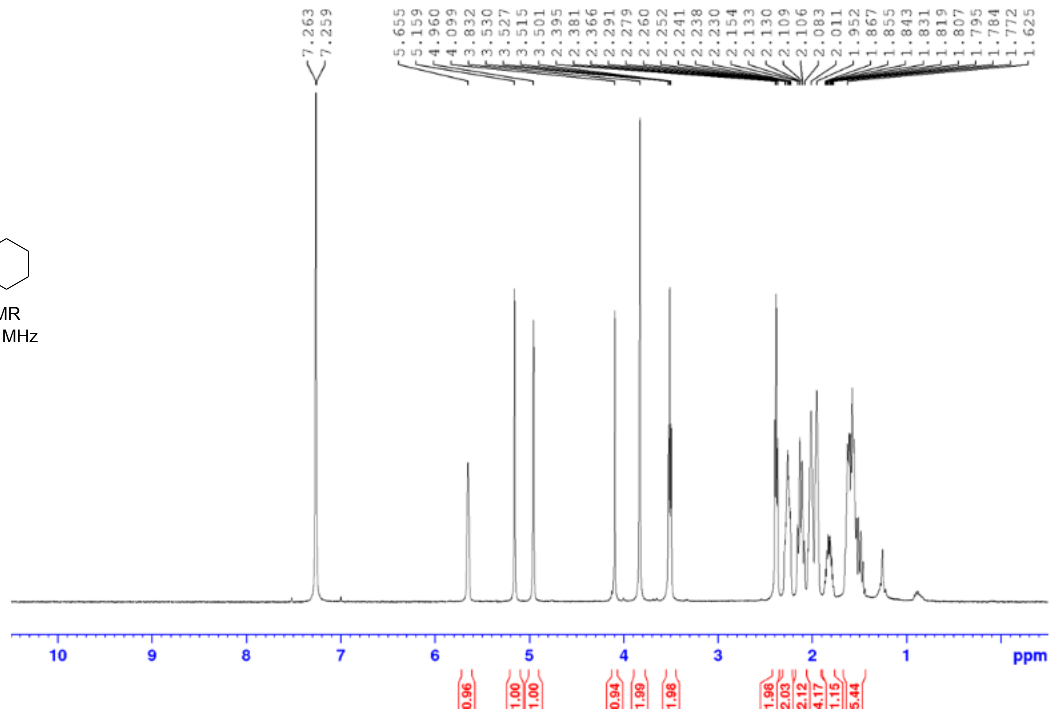

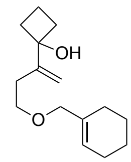

21, ^{13}C NMR
$CDCl_3$, 100 MHz

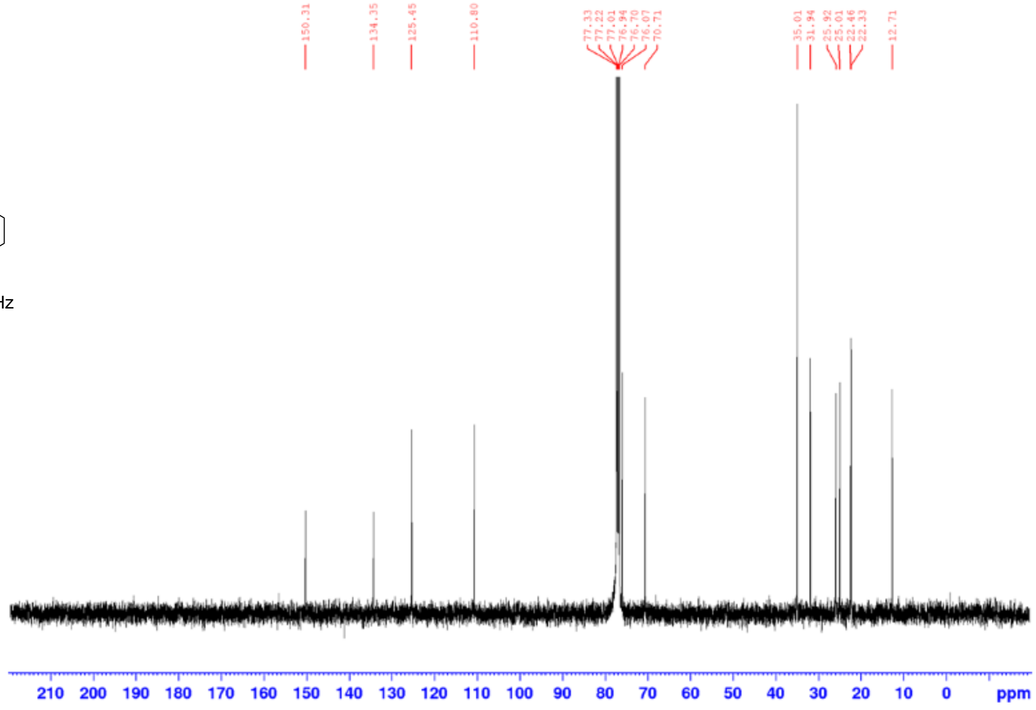

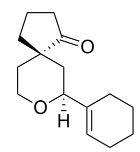

22, ^{1}H NMR
$CDCl_3$, 400 MHz

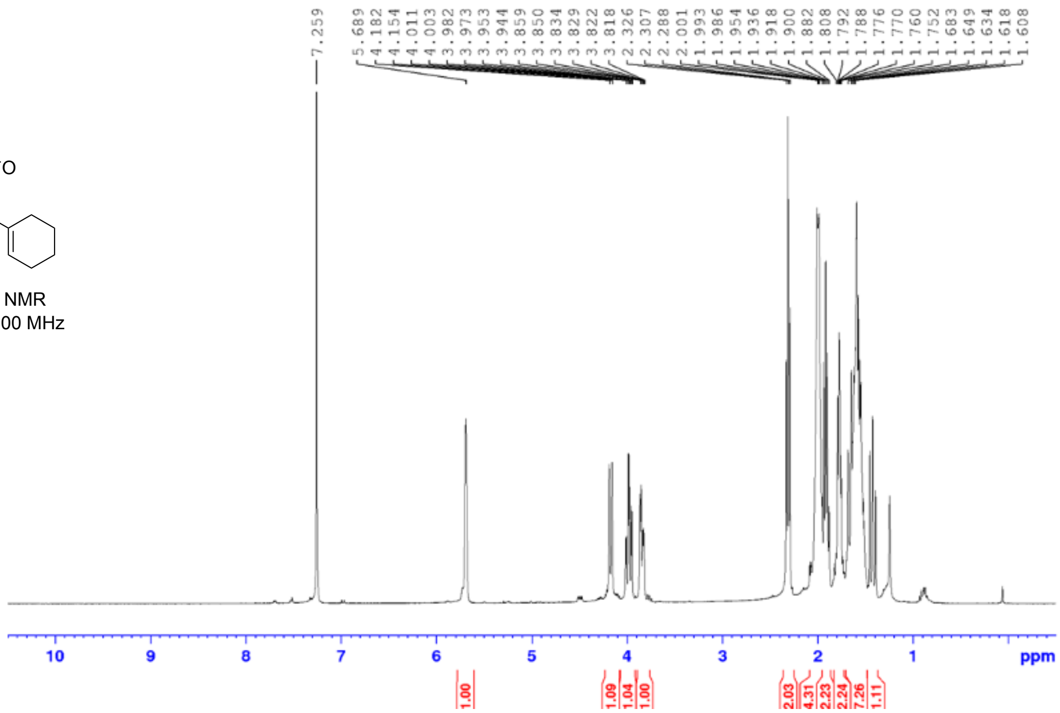

22, ^{13}C NMR
$CDCl_3$, 100 MHz

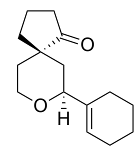

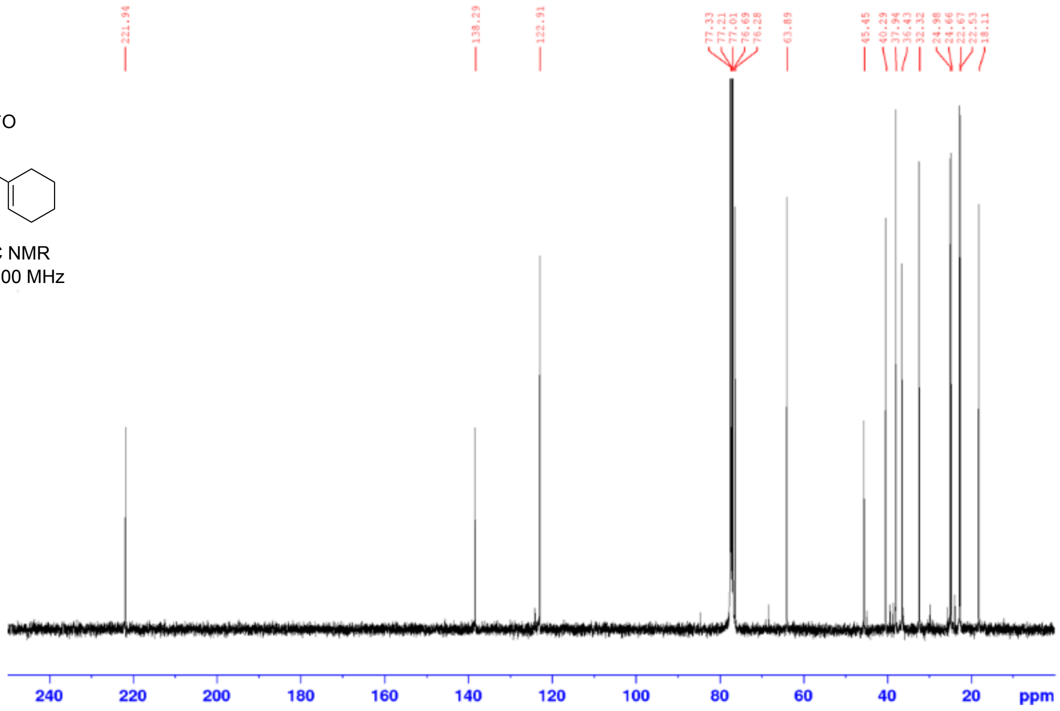

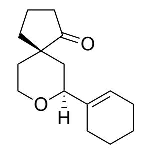

22, ¹H NMR, pure
$CDCl_3$, 300 MHz

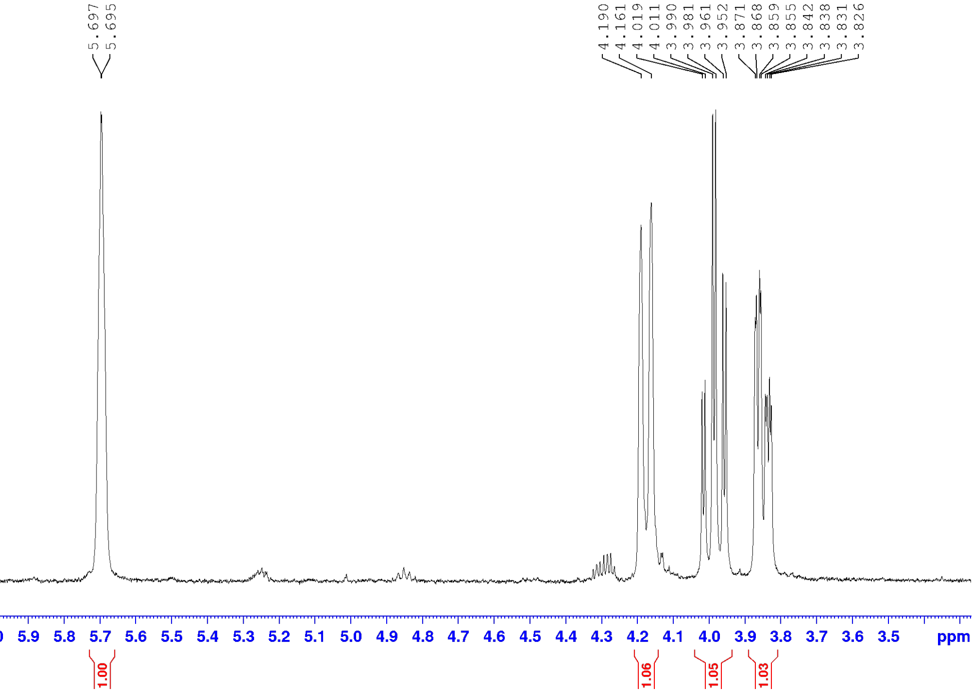

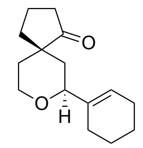

22, ¹H NMR, dr = 9:1
$CDCl_3$, 300 MHz

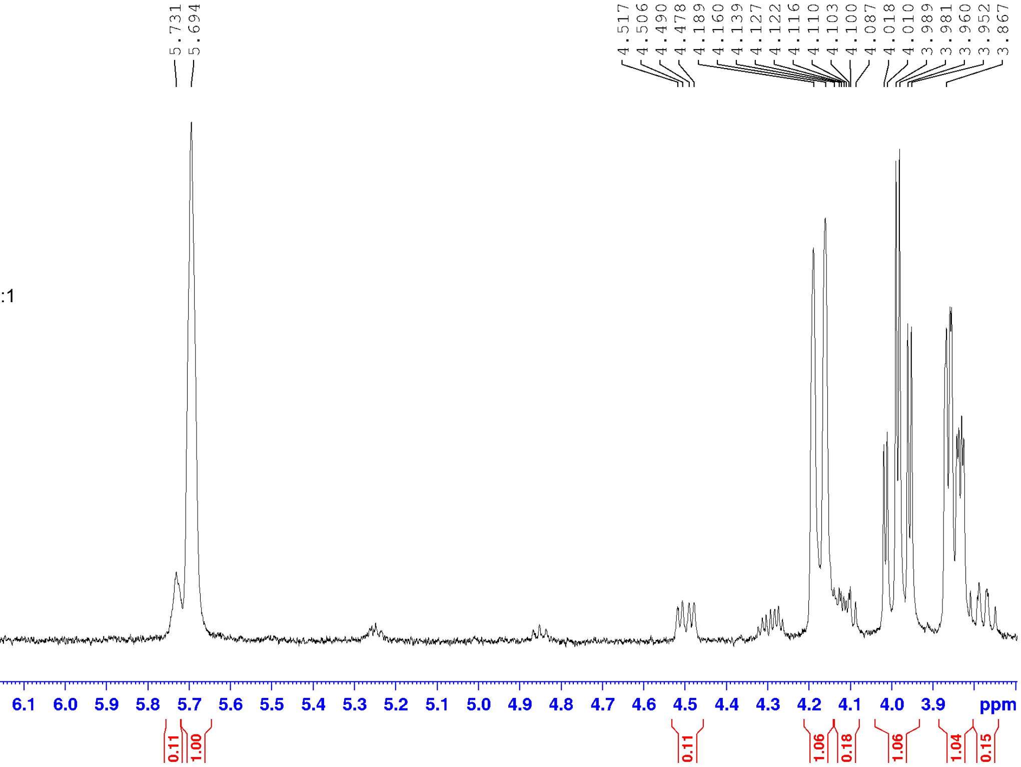

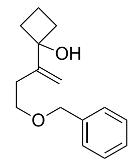

23, ^{1}H NMR
$CDCl_3$, 500 MHz

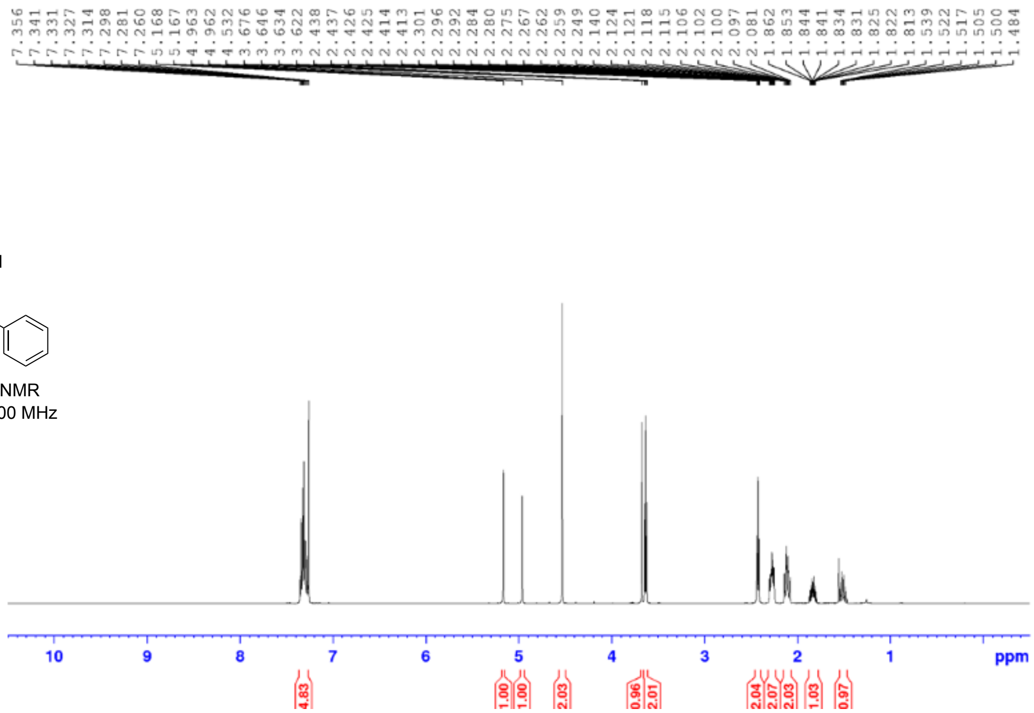

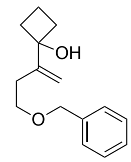

23, ^{13}C NMR
$CDCl_3$, 125 MHz

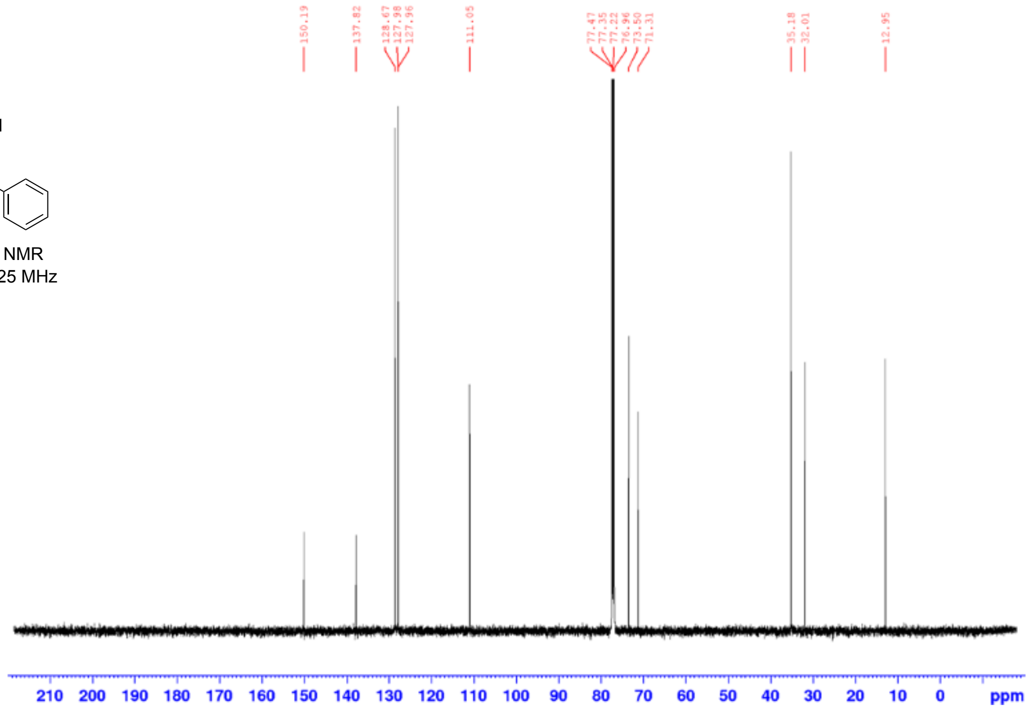

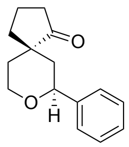

24, ^{1}H NMR
$CDCl_3$, 500 MHz

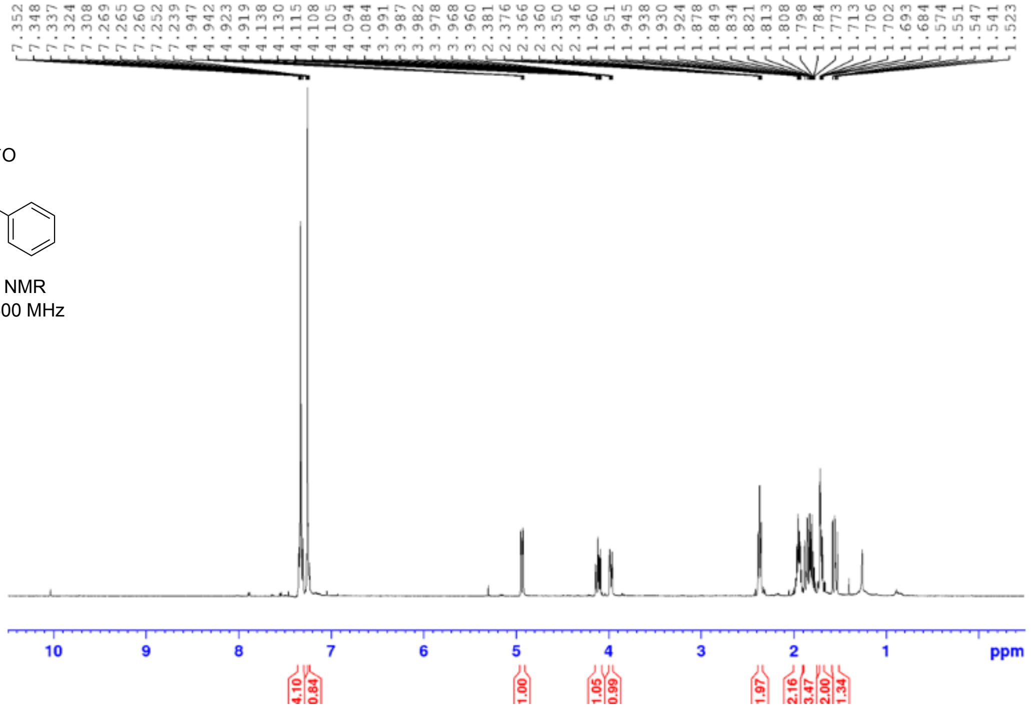

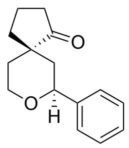

24, ^{13}C NMR
$CDCl_3$, 100 MHz

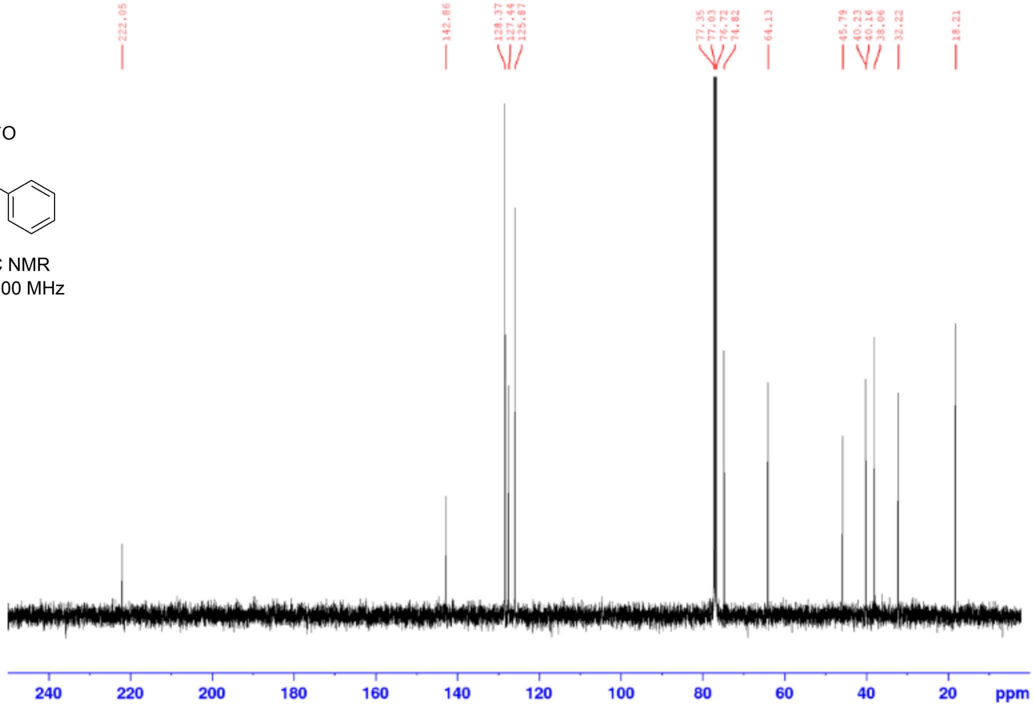

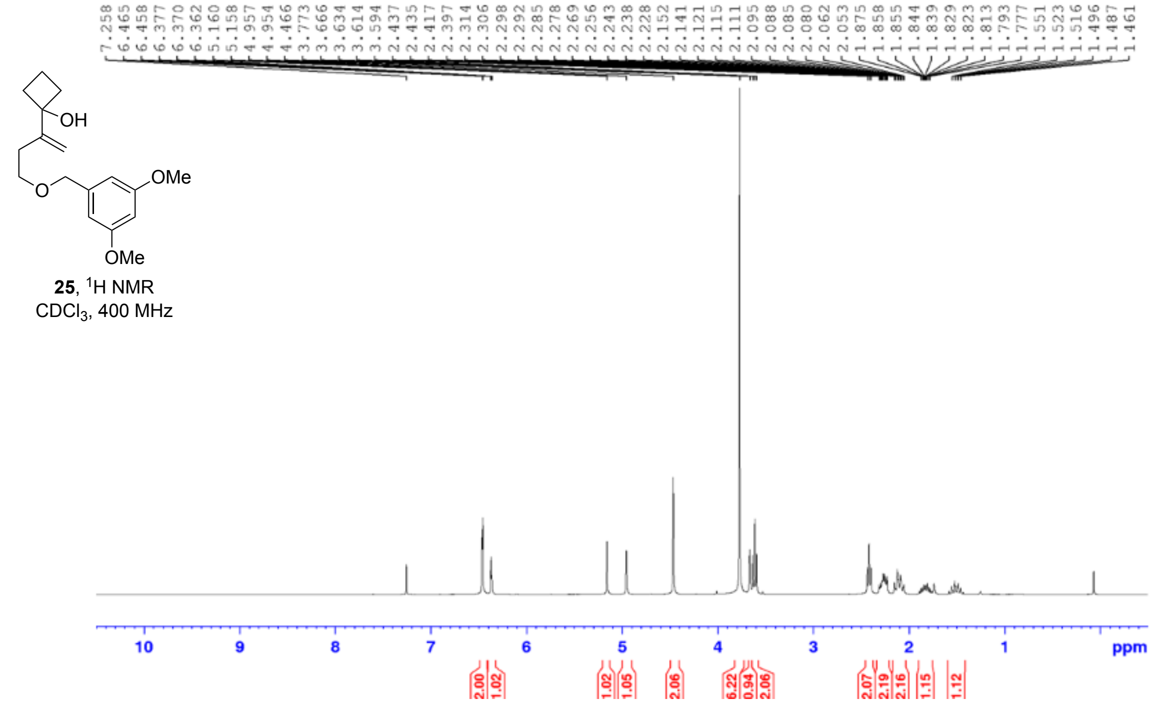

25, ^{1}H NMR
CDCl$_3$, 400 MHz

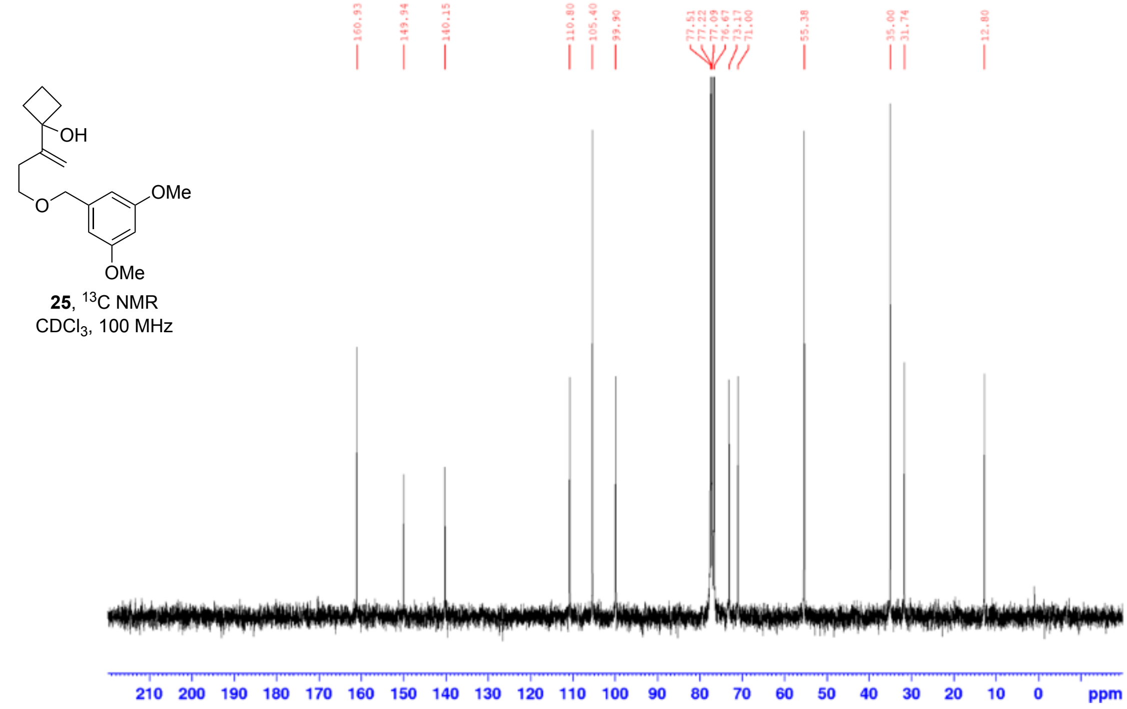

25, ^{13}C NMR
CDCl$_3$, 100 MHz

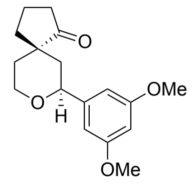

26, ^{1}H NMR
$CDCl_3$, 300 MHz

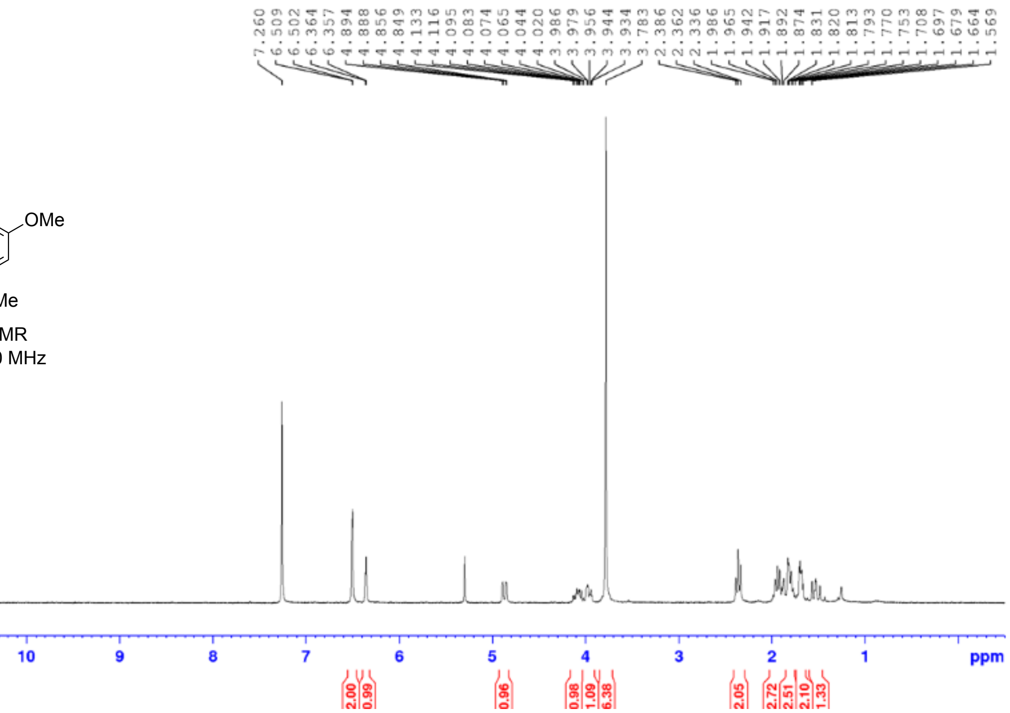

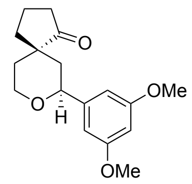

26, ^{13}C NMR
$CDCl_3$, 100 MHz

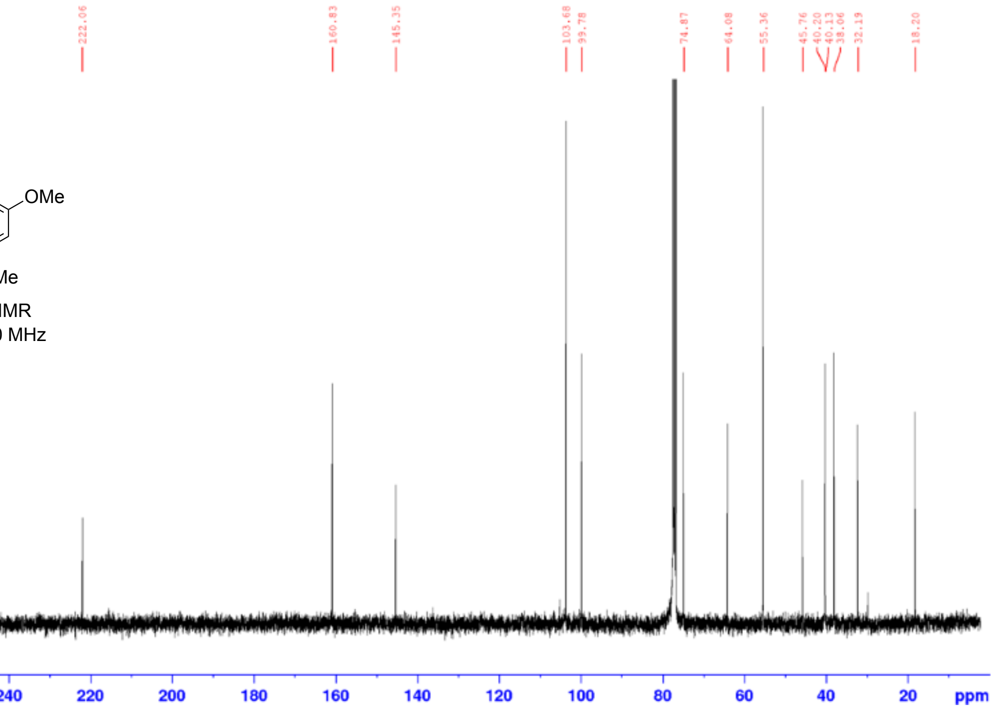

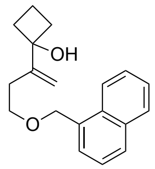

27, ^{1}H NMR
$CDCl_3$, 500 MHz

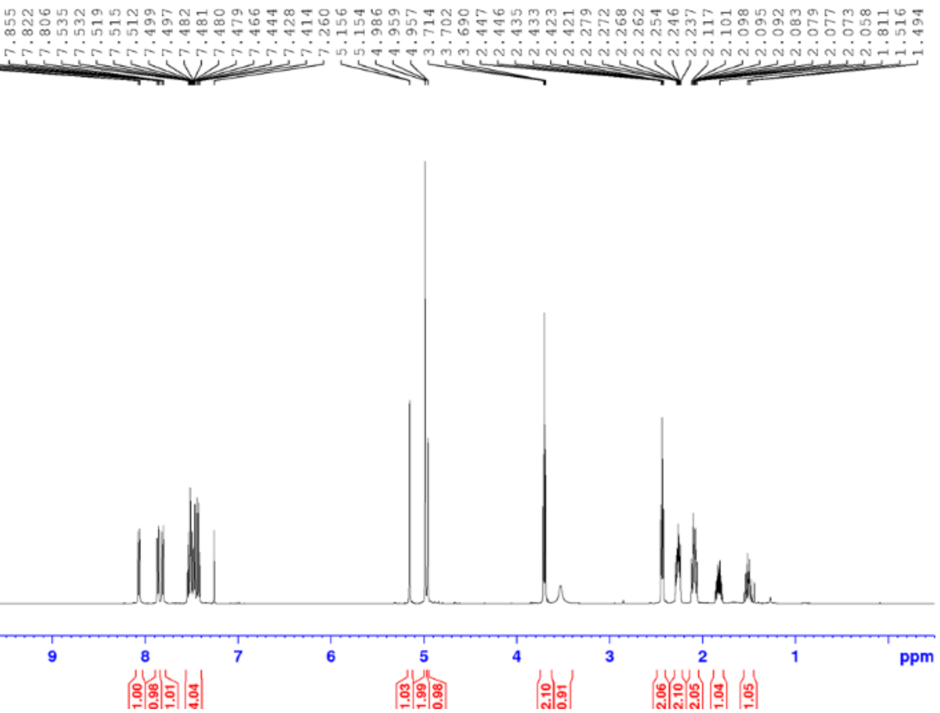

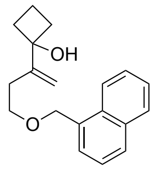

27, ^{13}C NMR
$CDCl_3$, 125 MHz

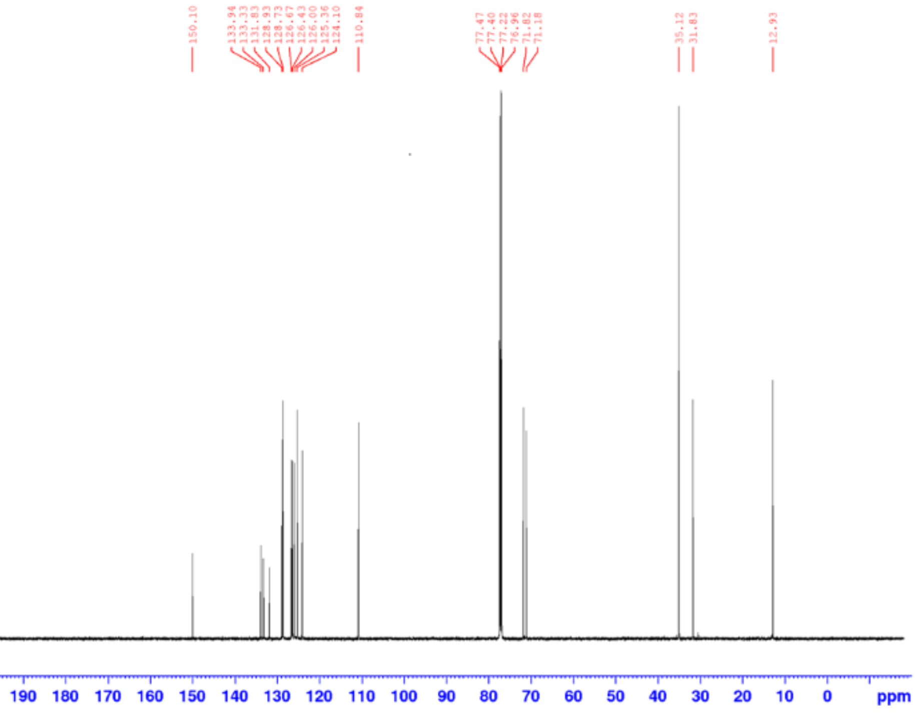

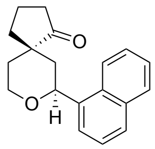

28, ^{1}H NMR
$CDCl_3$, 400 MHz

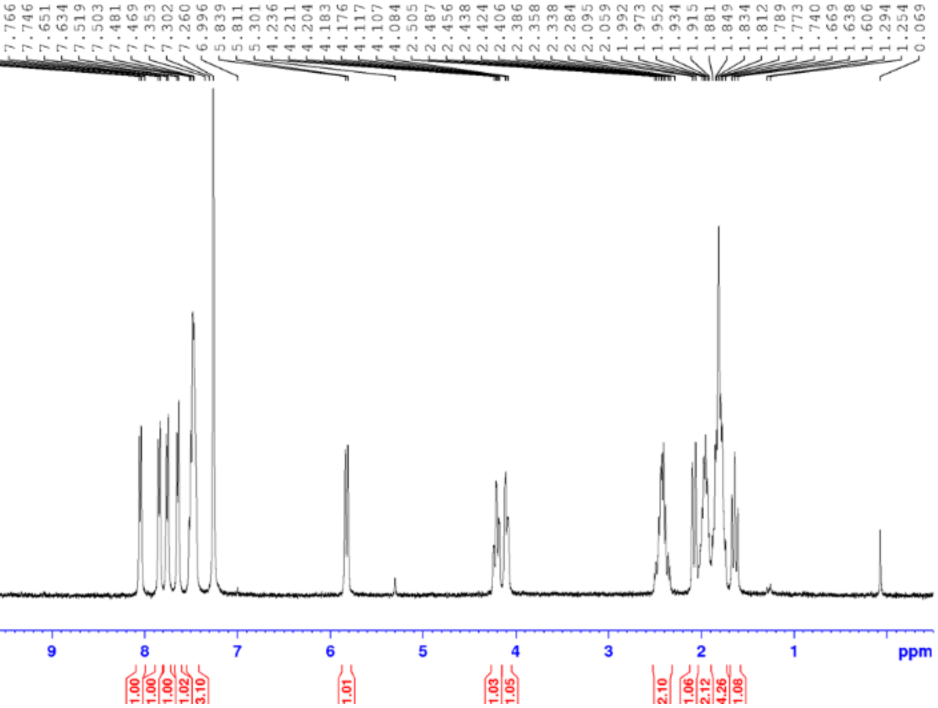

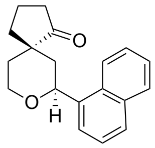

28, ^{13}C NMR
$CDCl_3$, 100 MHz

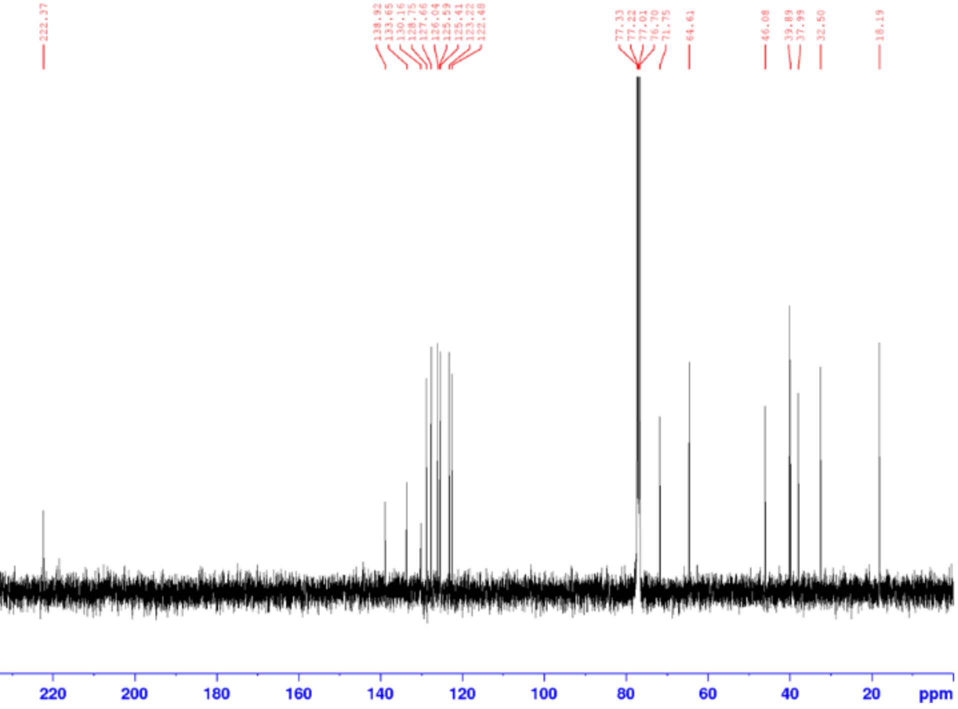

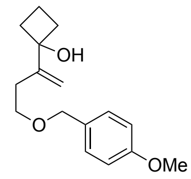

29, ^{1}H NMR
$CDCl_3$, 300 MHz

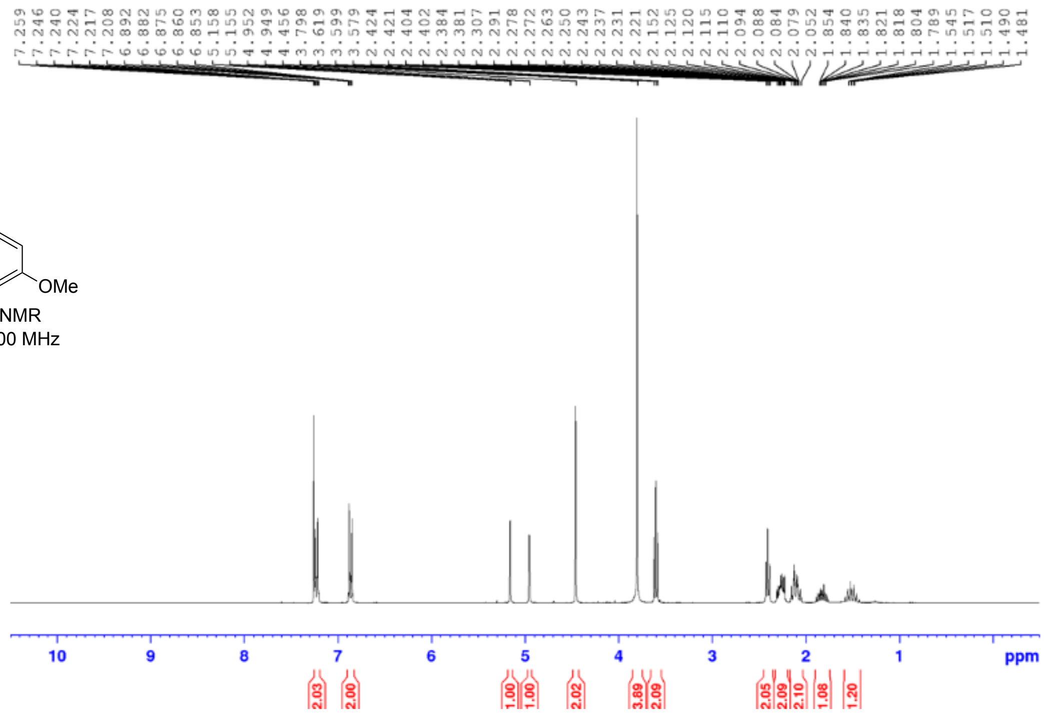

29, ^{13}C NMR
$CDCl_3$, 100 MHz

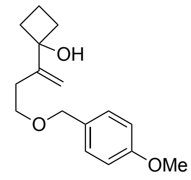

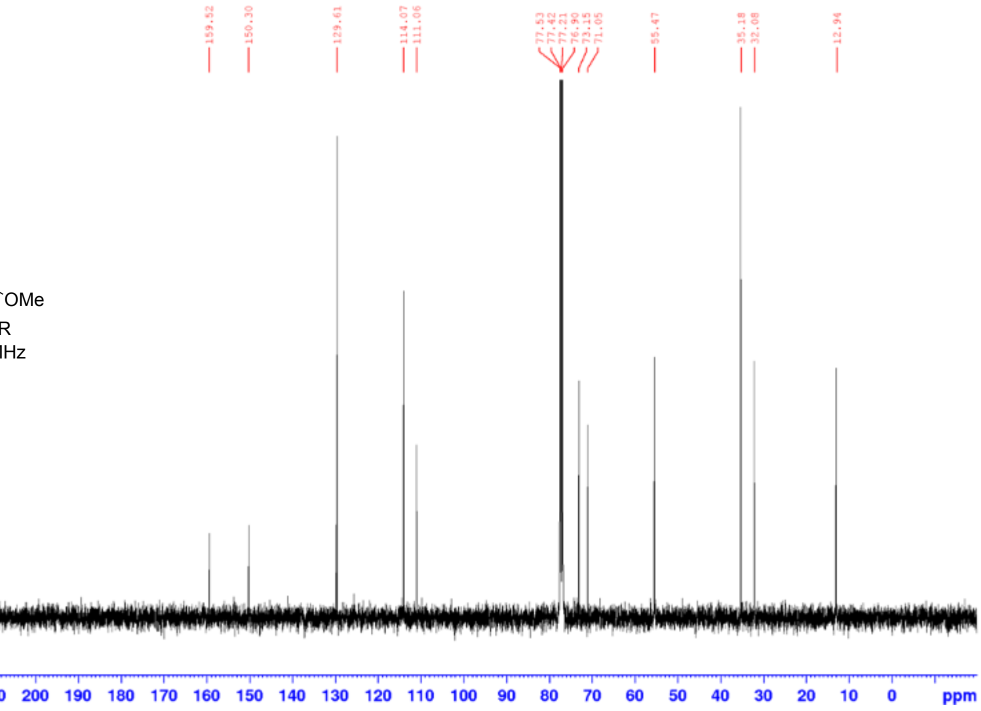

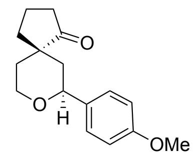

30, ^{1}H NMR
$CDCl_3$, 300 MHz

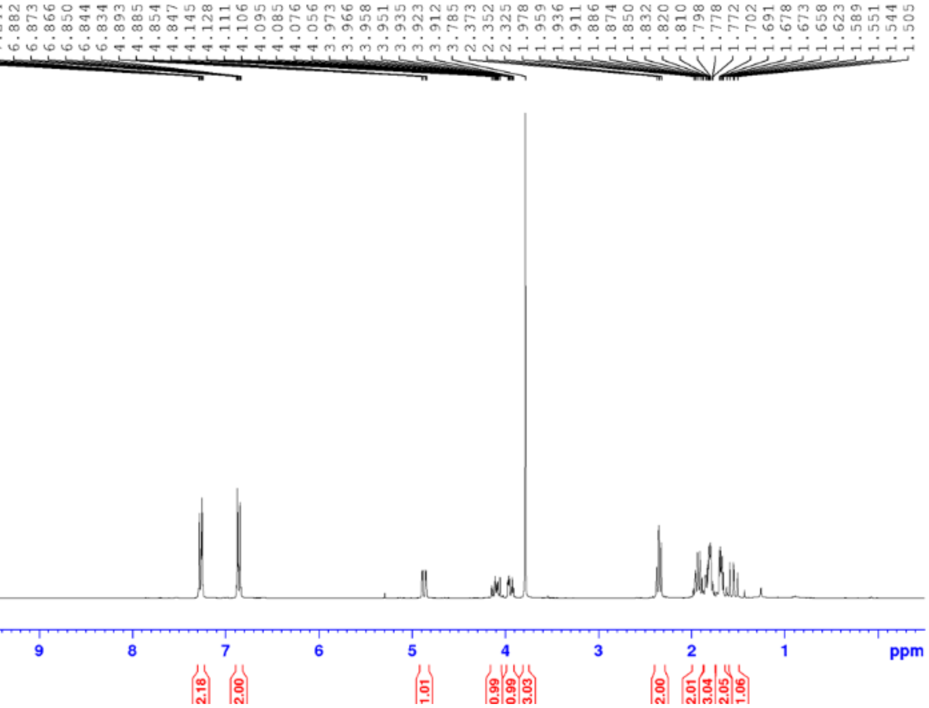

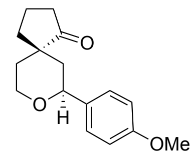

30, ^{13}C NMR
$CDCl_3$, 125 MHz

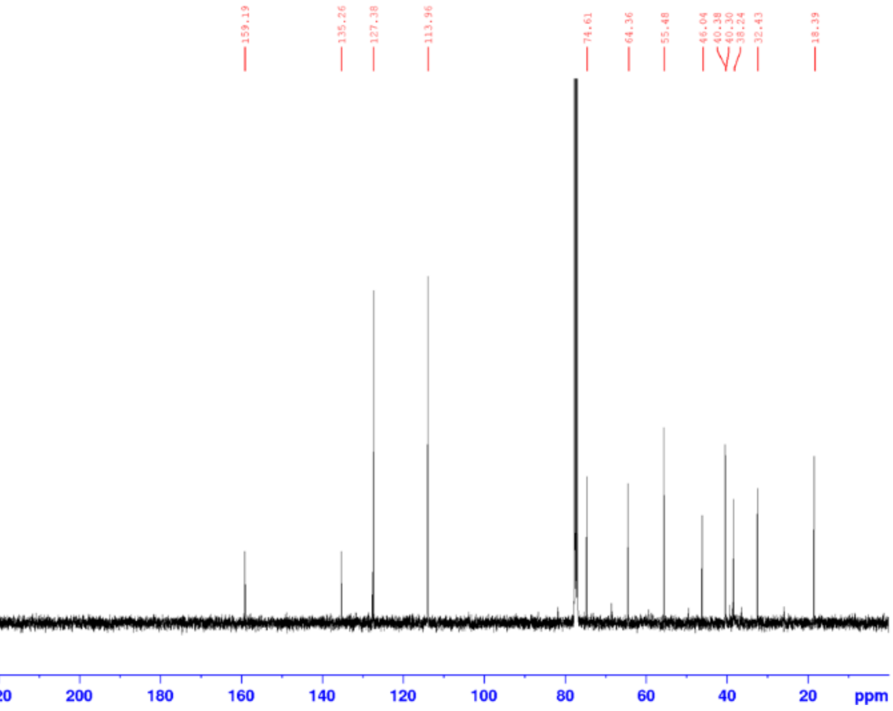

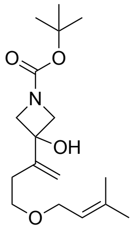

31, ^{1}H NMR
$CDCl_3$, 400 MHz

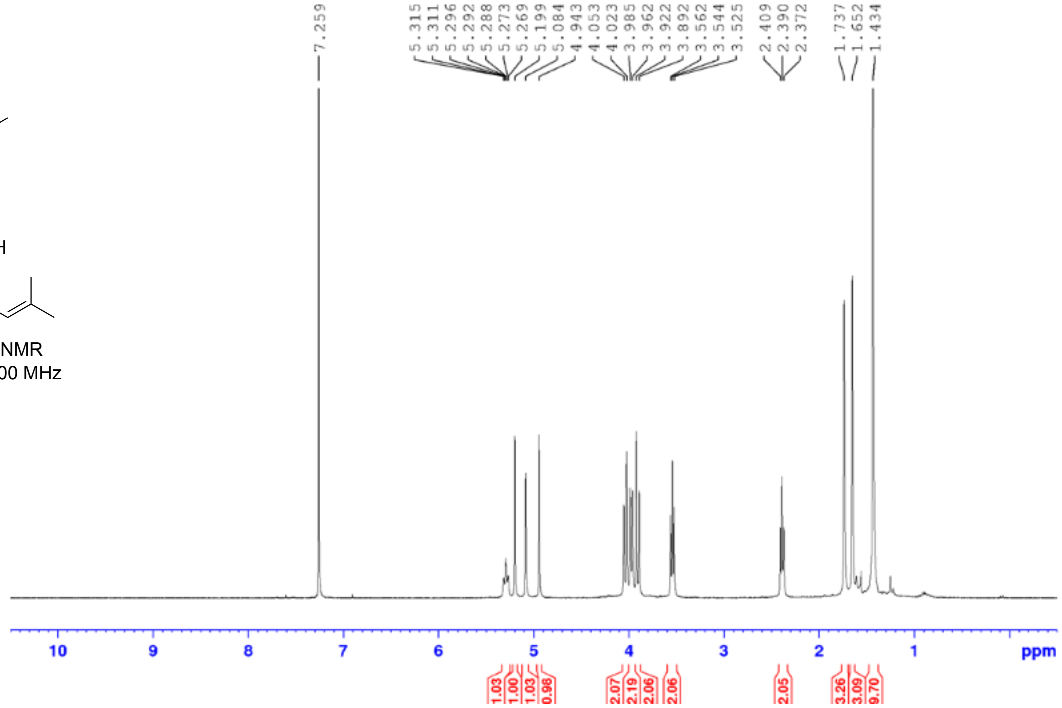

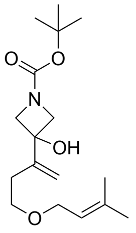

31, ^{13}C NMR
$CDCl_3$, 75 MHz

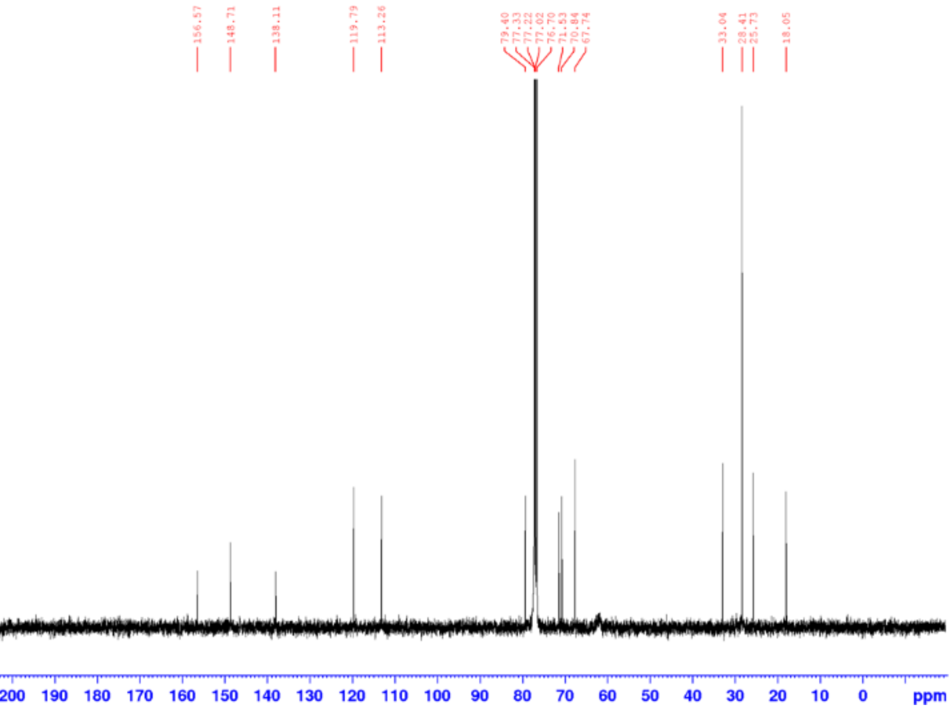

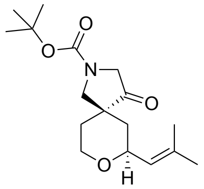

32, ^{1}H NMR
$CDCl_3$, 300 MHz

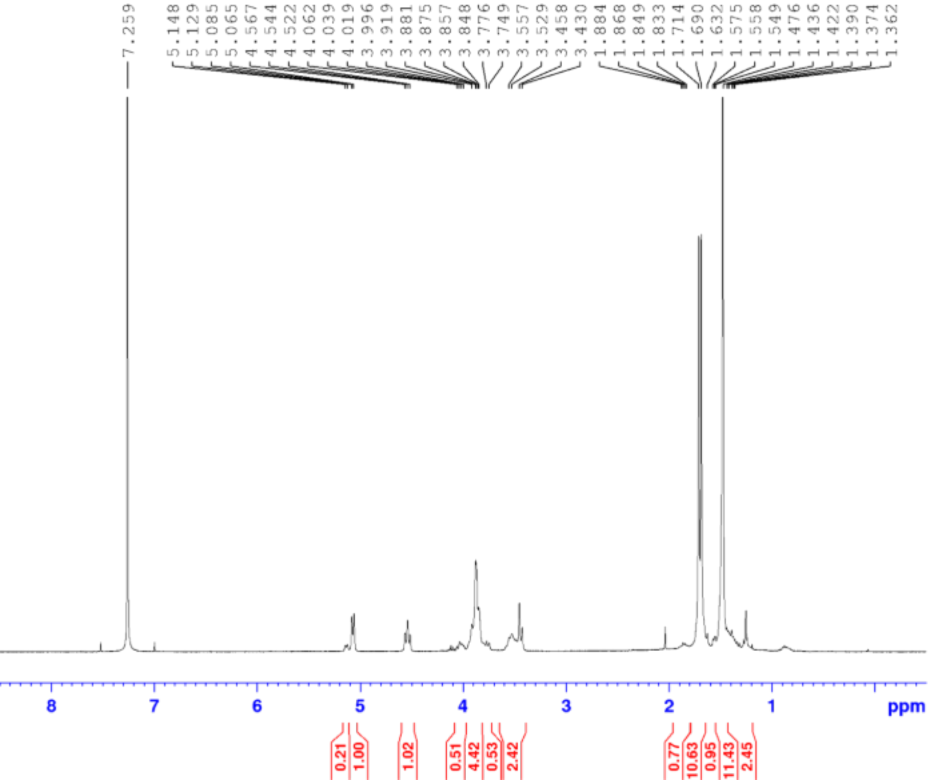

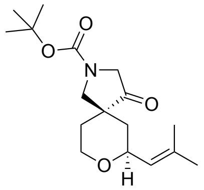

32, ^{13}C NMR
acetone-d6, 100 MHz

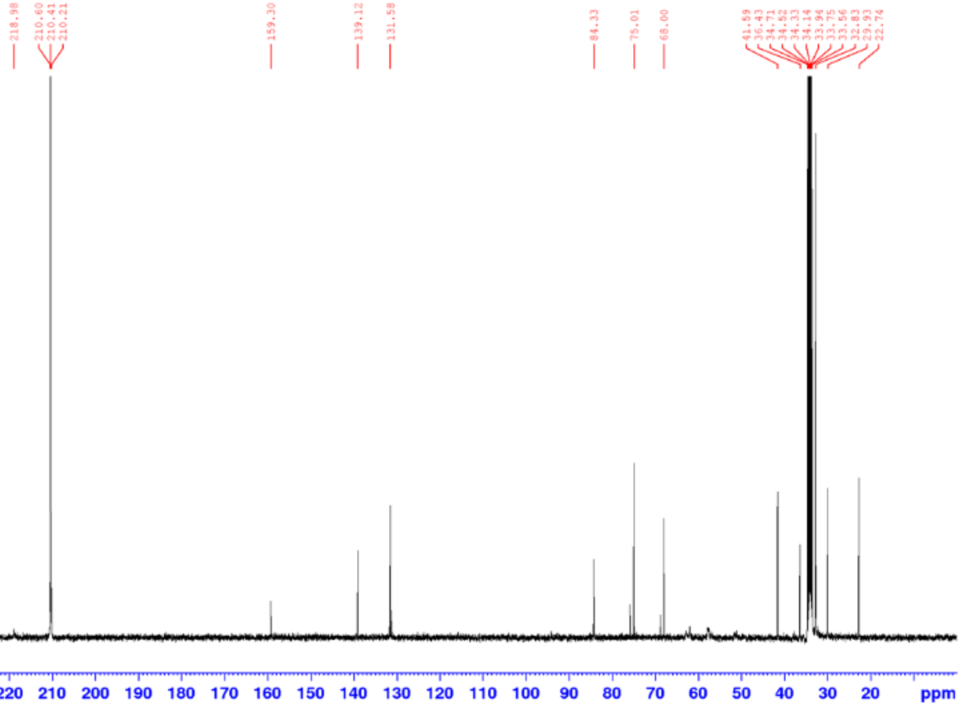

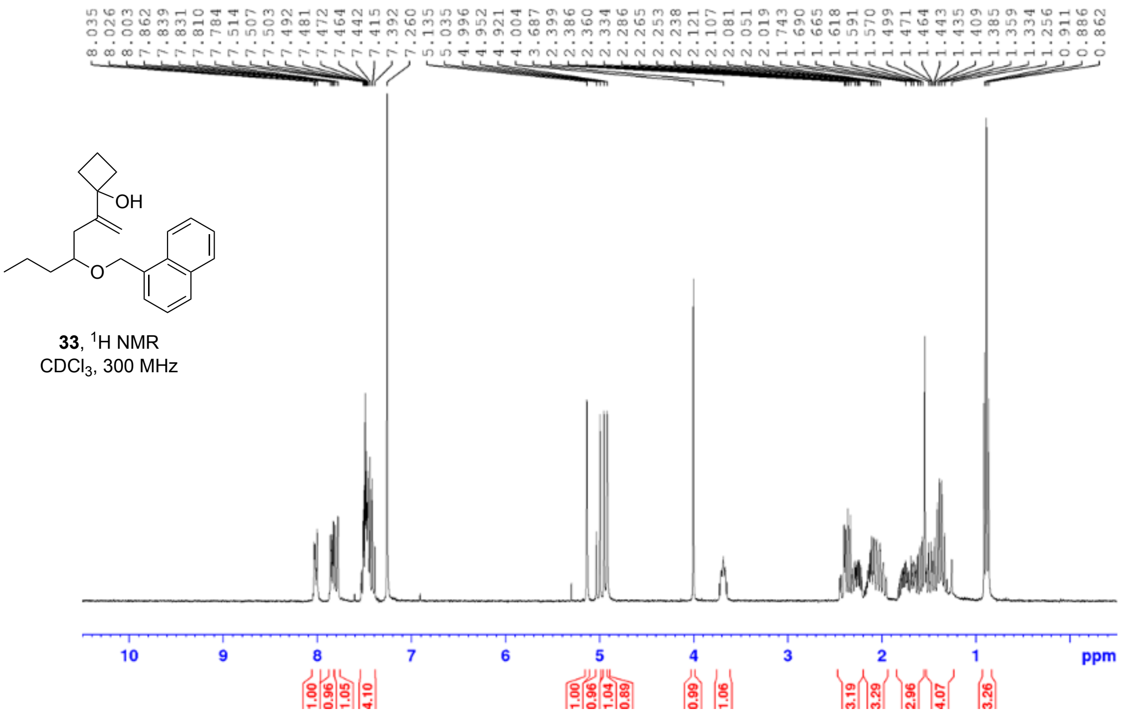

33, ^{1}H NMR
$CDCl_3$, 300 MHz

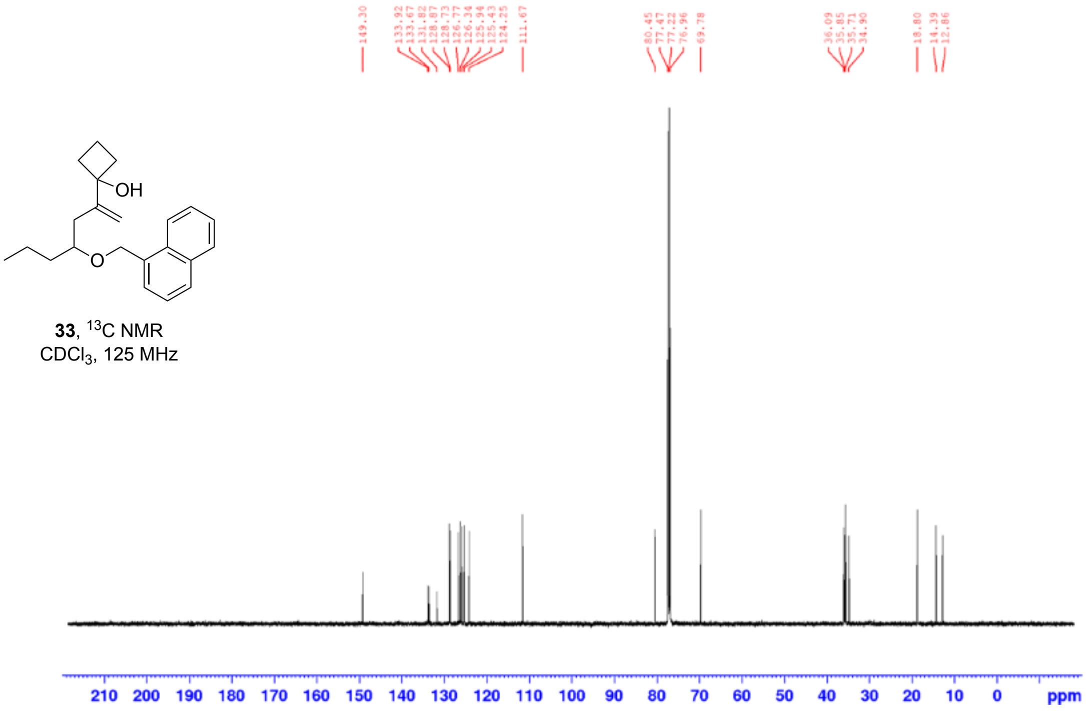

33, ^{13}C NMR
$CDCl_3$, 125 MHz

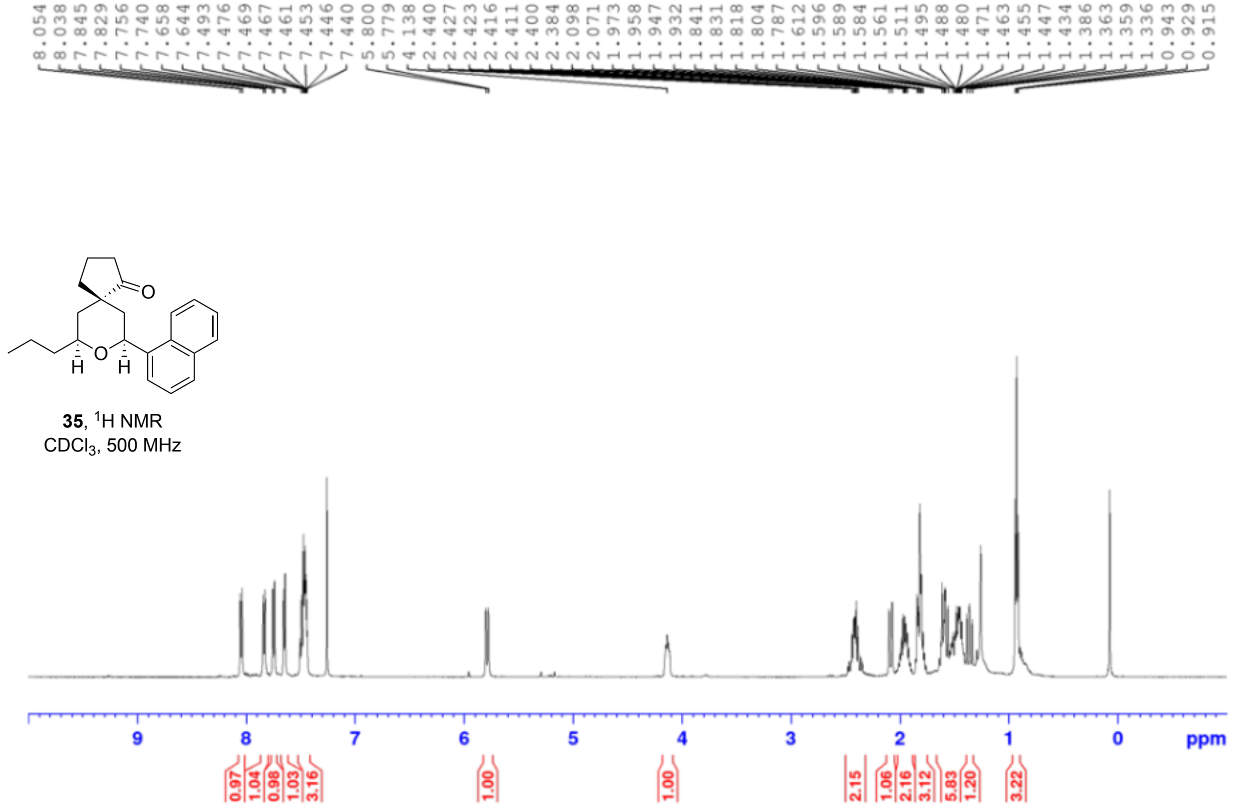

35, ^{1}H NMR
$CDCl_3$, 500 MHz

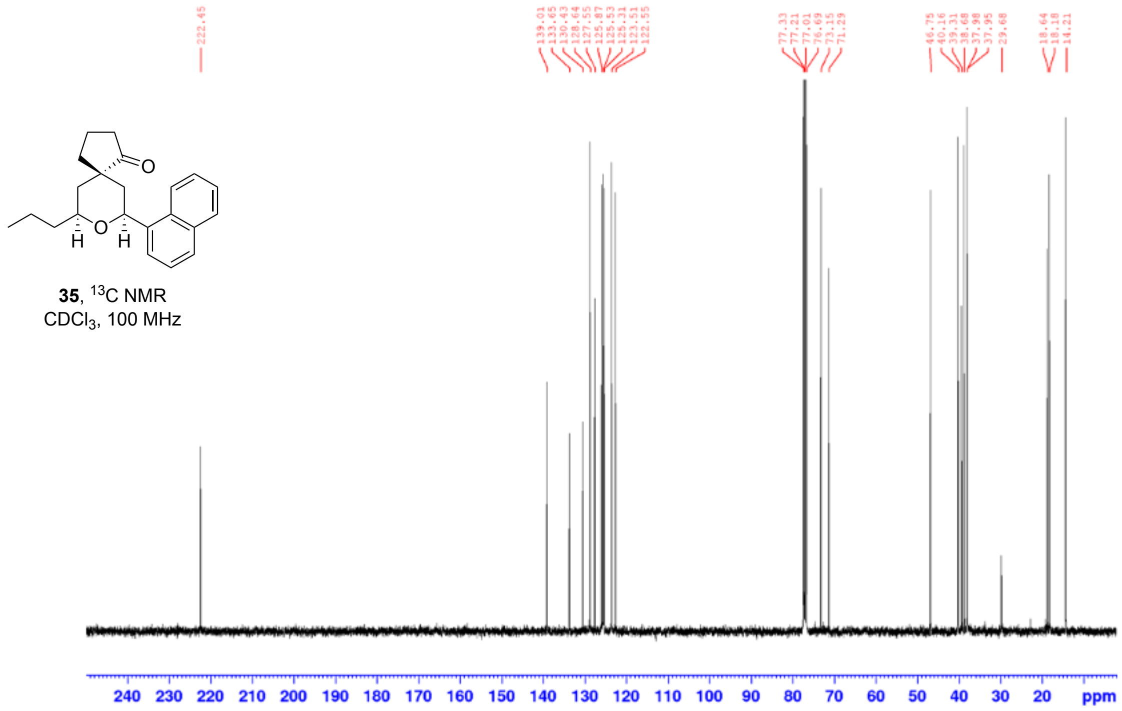

35, ^{13}C NMR
$CDCl_3$, 100 MHz

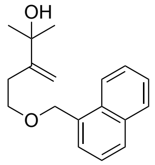

36, ^{1}H NMR
CDCl$_3$, 300 MHz

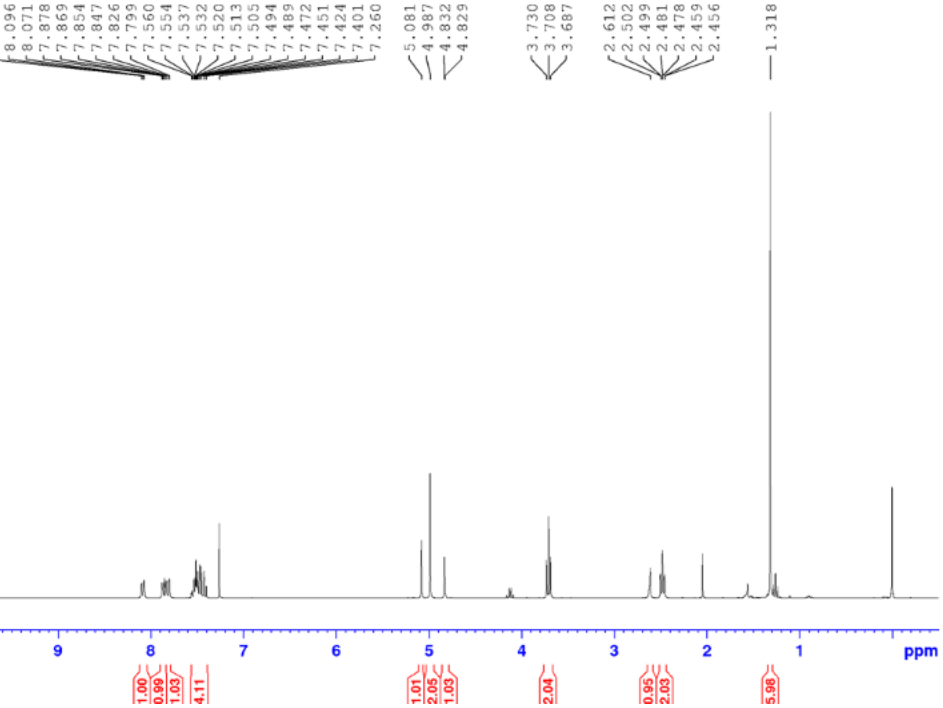

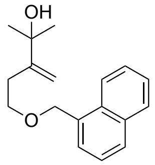

36, ^{13}C NMR
CDCl$_3$, 125 MHz

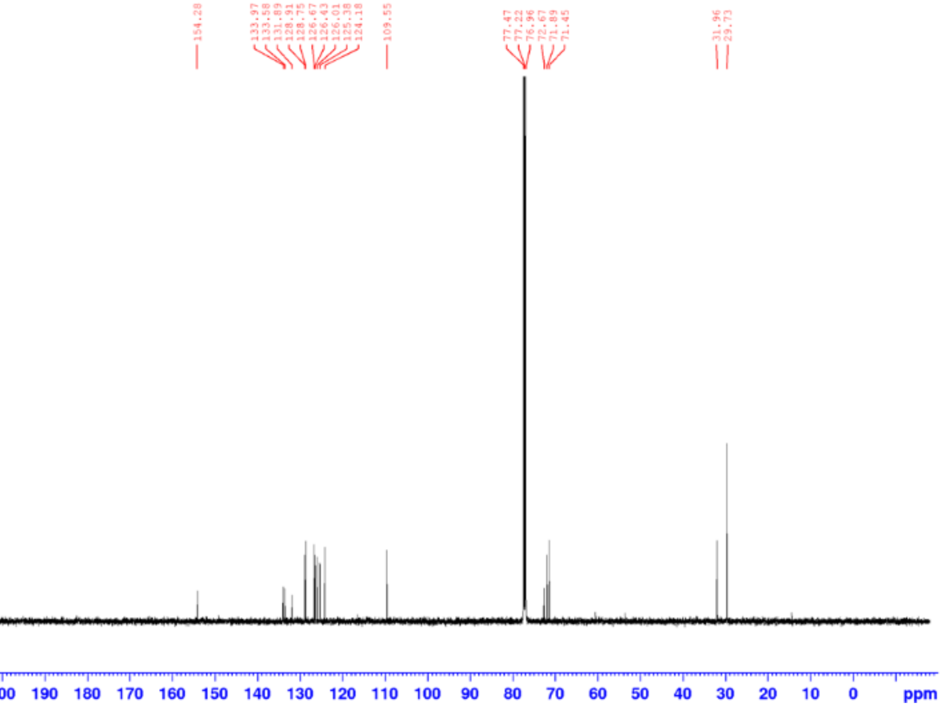

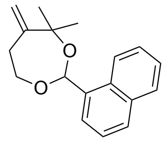

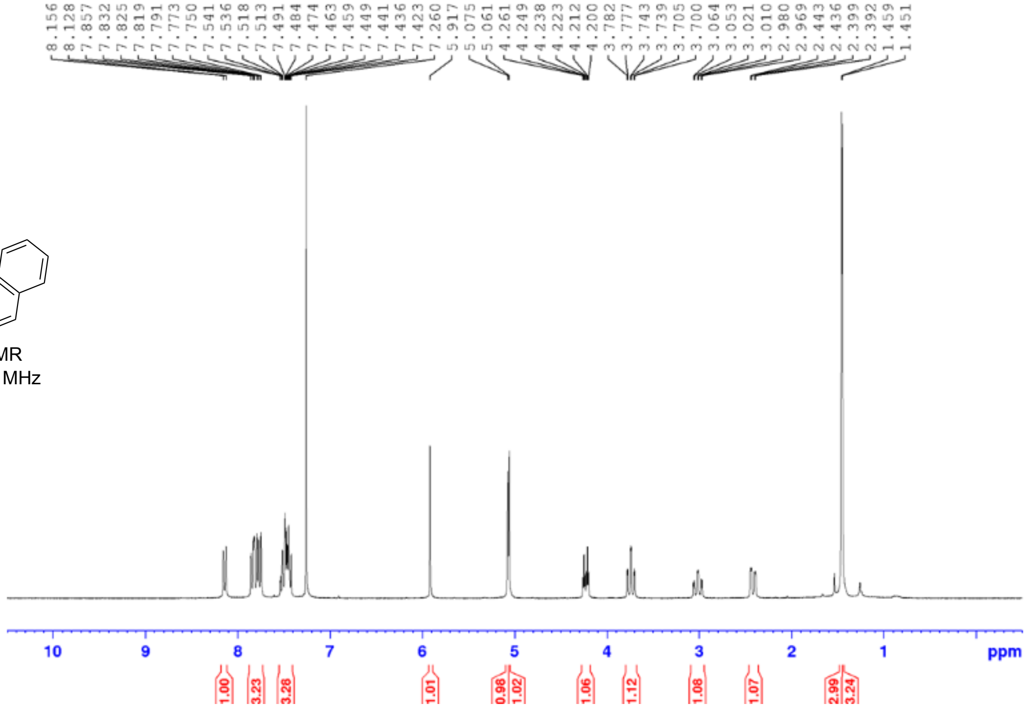

37, ^{1}H NMR
$CDCl_3$, 400 MHz

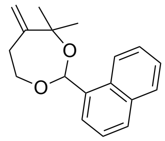

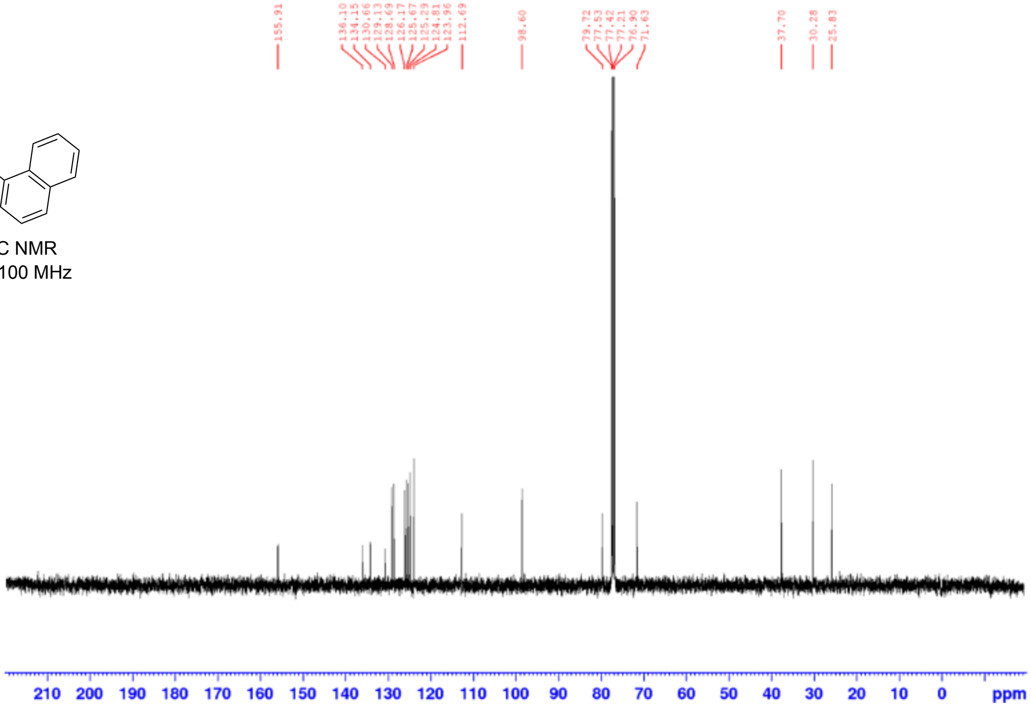

37, ^{13}C NMR
$CDCl_3$, 100 MHz

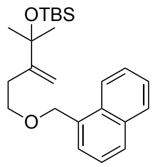

38, ^{1}H NMR
$CDCl_3$, 300 MHz

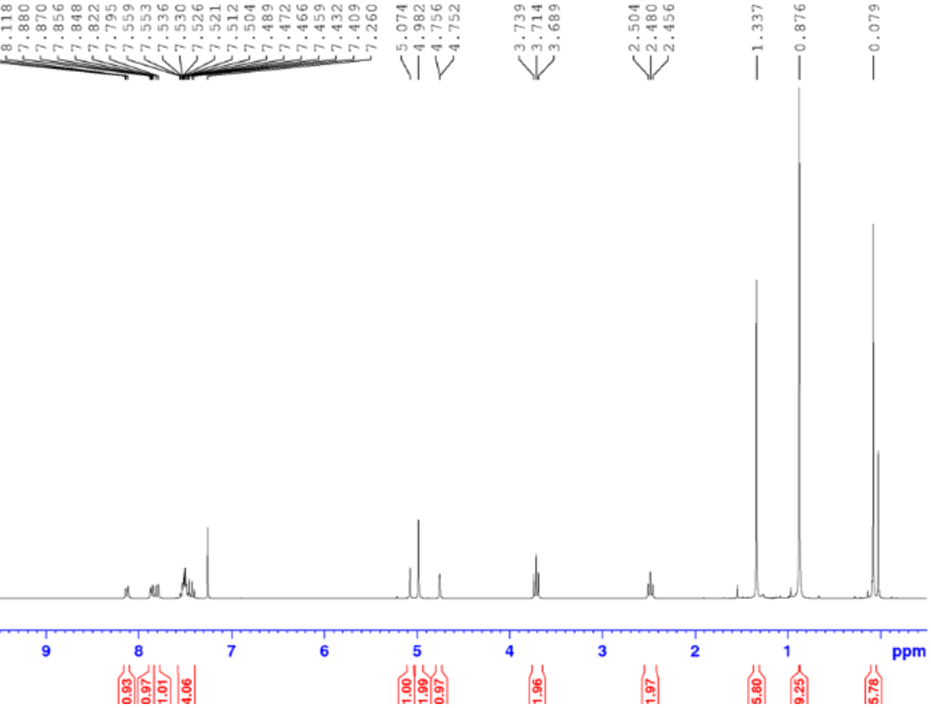

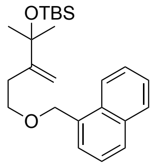

38, ^{13}C NMR
$CDCl_3$, 125 MHz

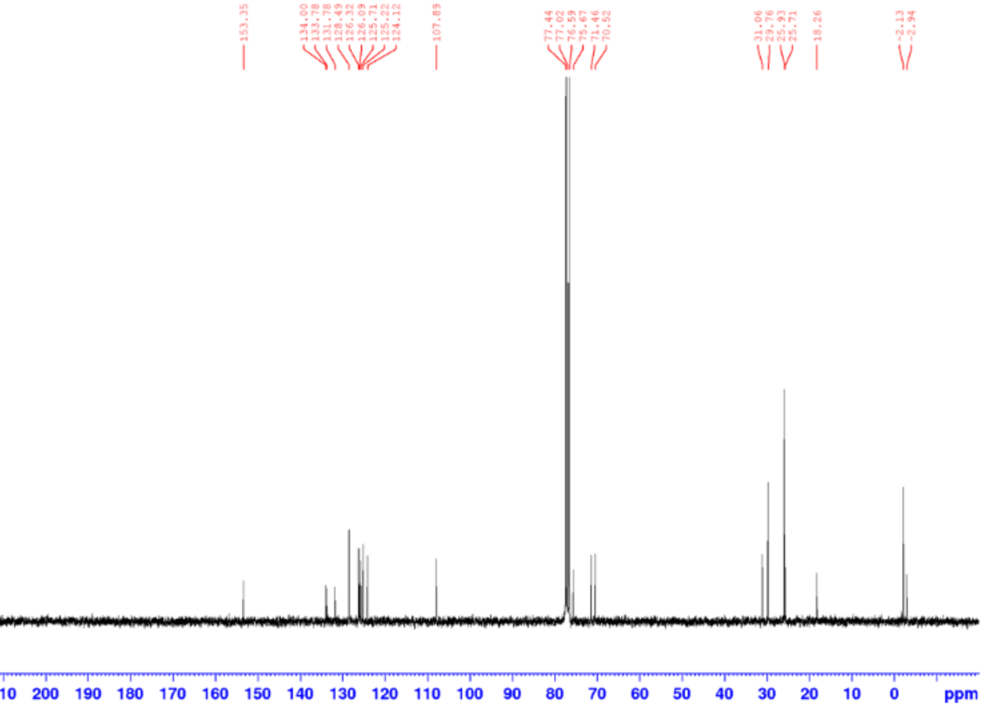

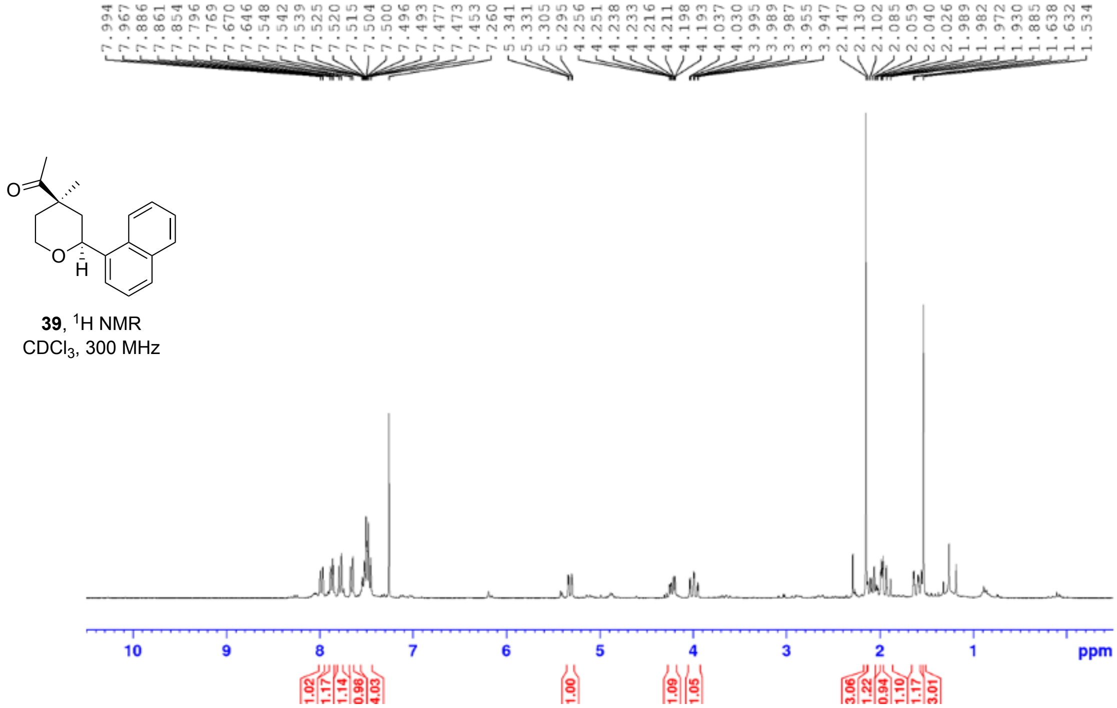

39, ^{1}H NMR
$CDCl_3$, 300 MHz

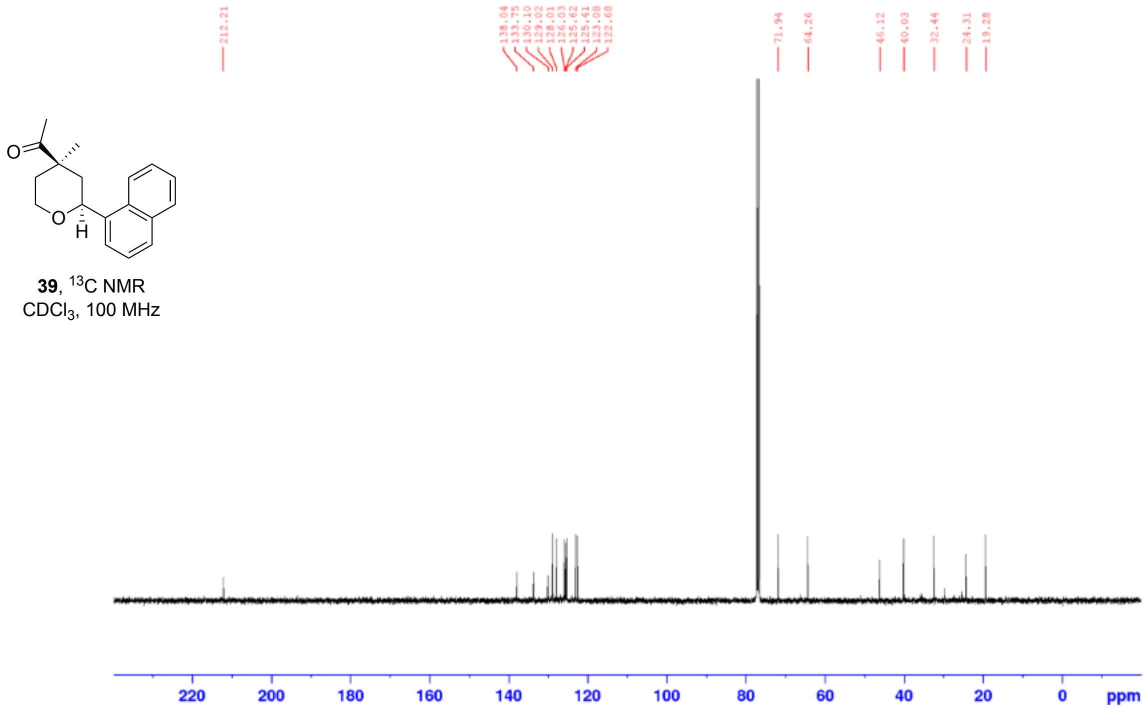

39, ^{13}C NMR
$CDCl_3$, 100 MHz

39 NOESY $CDCl_3$, 500 MHz

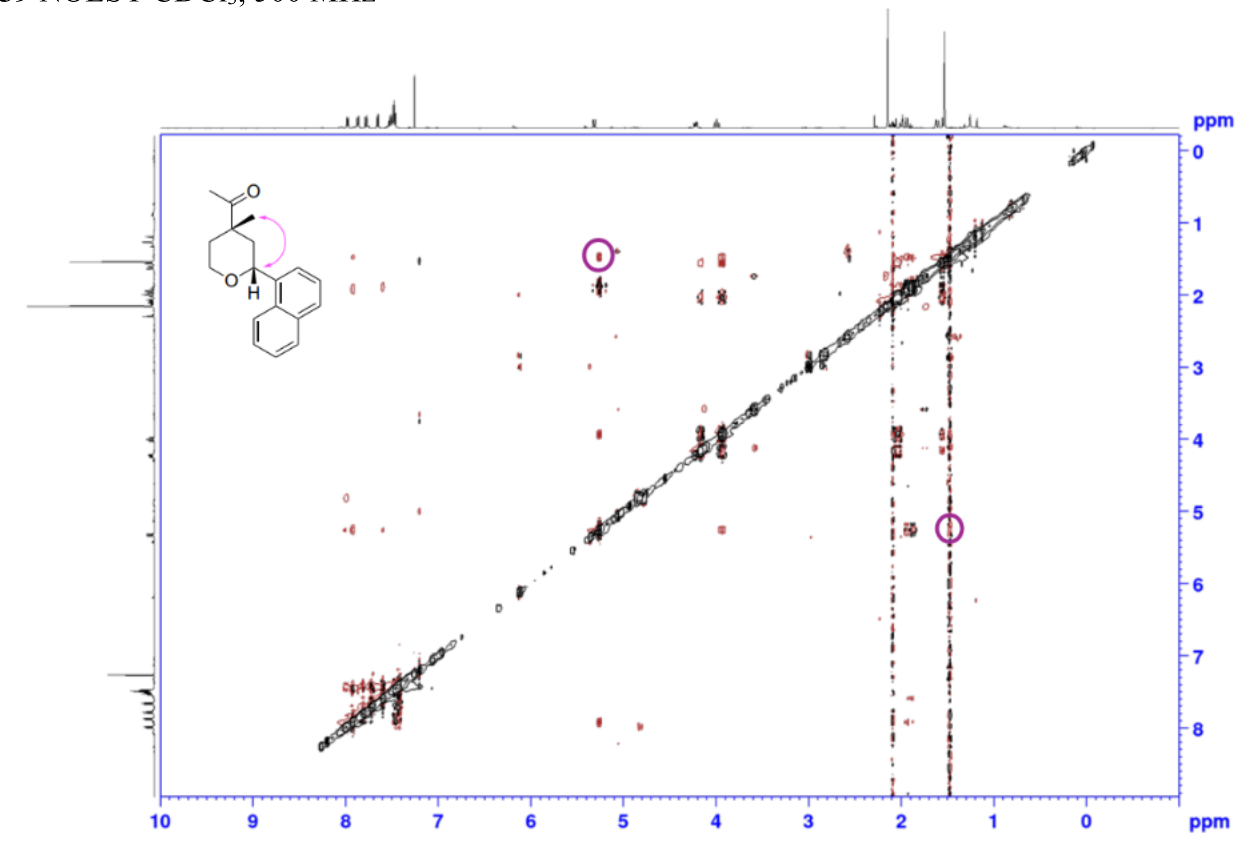

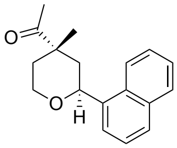

39', ^{1}H NMR
$CDCl_3$, 300 MHz

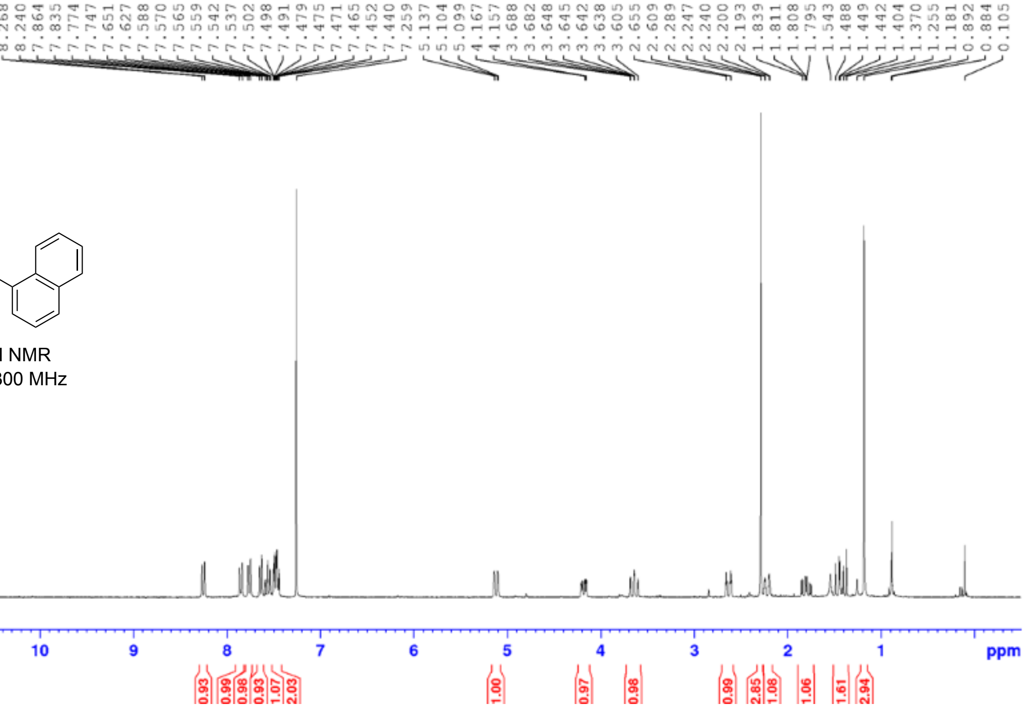

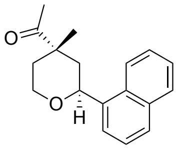

39', ^{13}C NMR
$CDCl_3$, 100 MHz

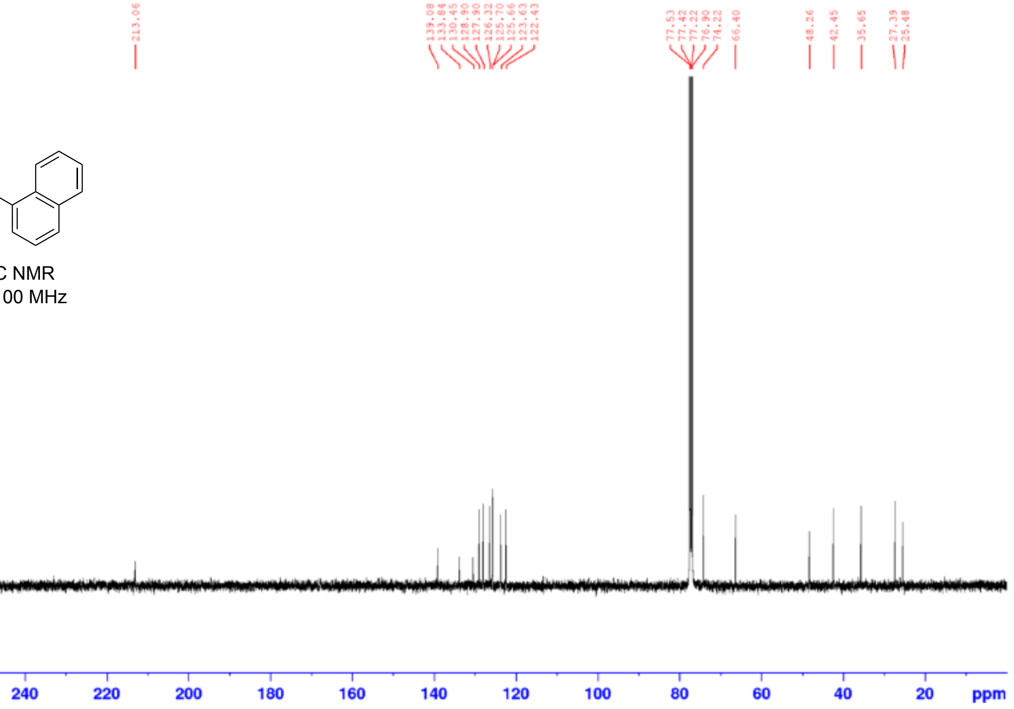

39' NOESY, $CDCl_3$, 500 MHz

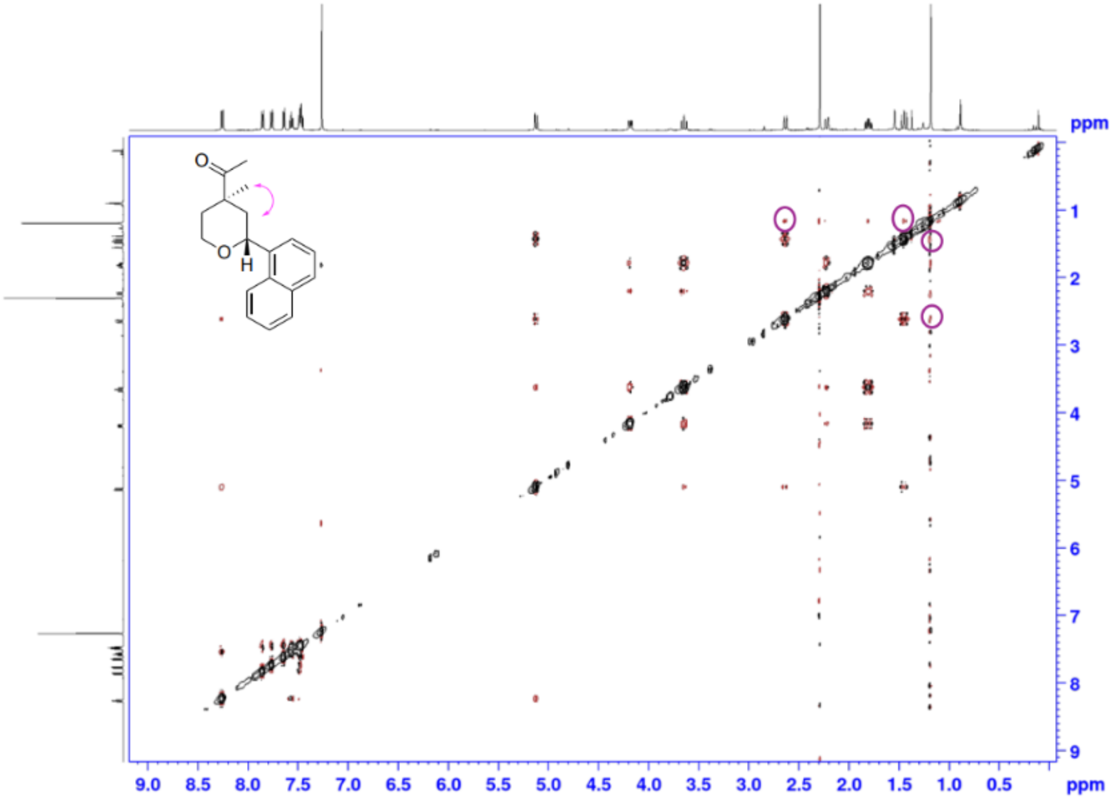

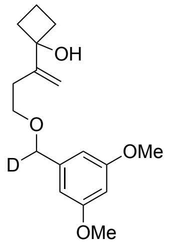

25-D$_1$, ^{1}H NMR
300 MHz, $CDCl_3$

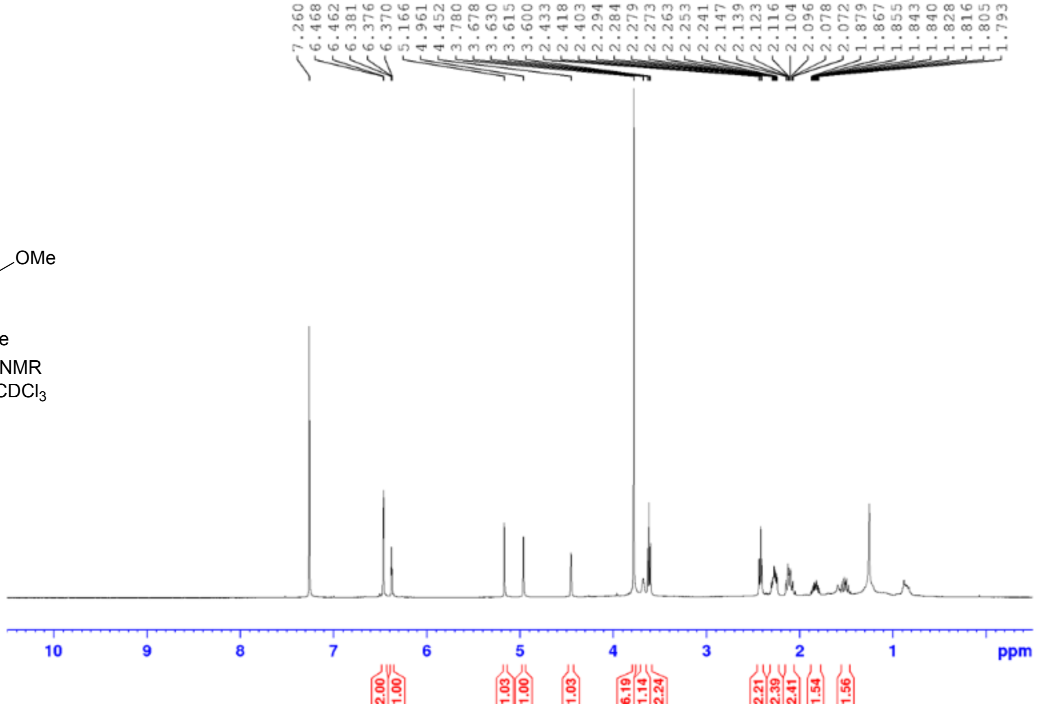

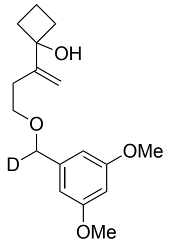

25-D$_1$, ^{13}C NMR
100 MHz, $CDCl_3$

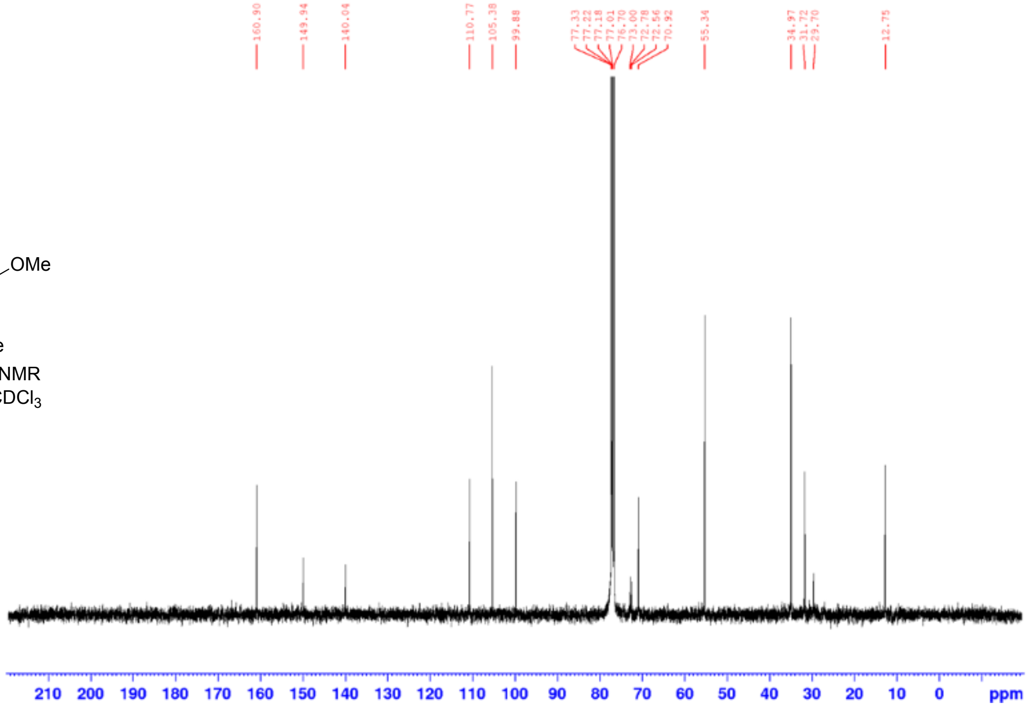

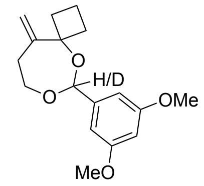

40-H/D, ^{1}H NMR
$CDCl_3$, 400 MHz

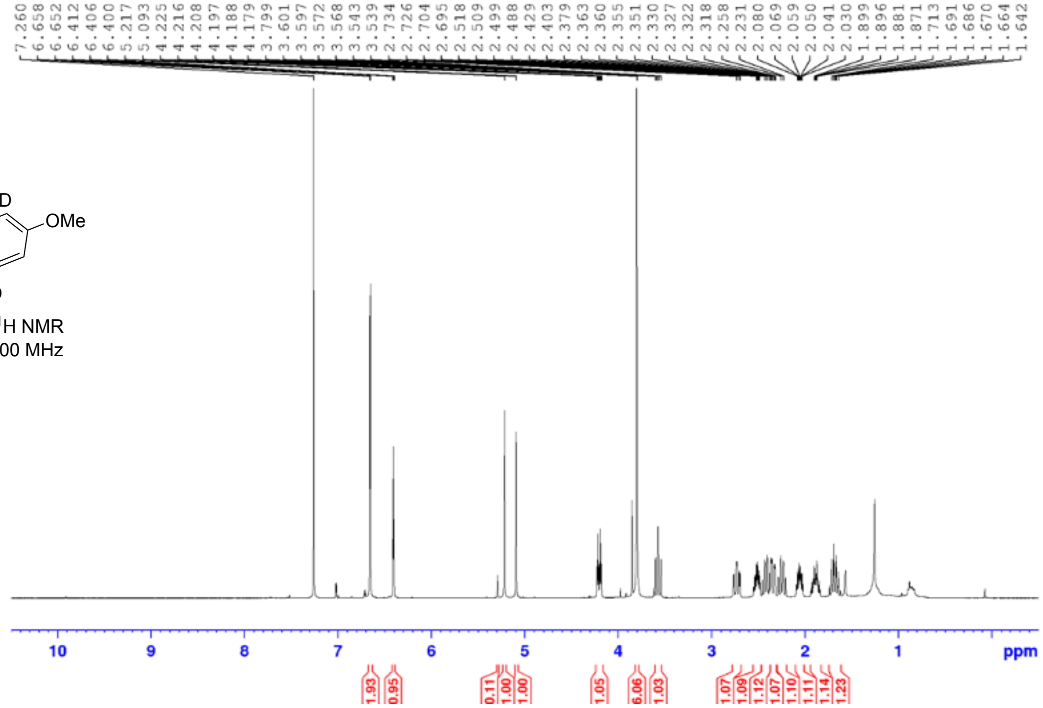

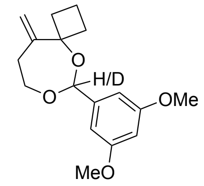

40-H/D, ^{13}C NMR
$CDCl_3$, 100 MHz

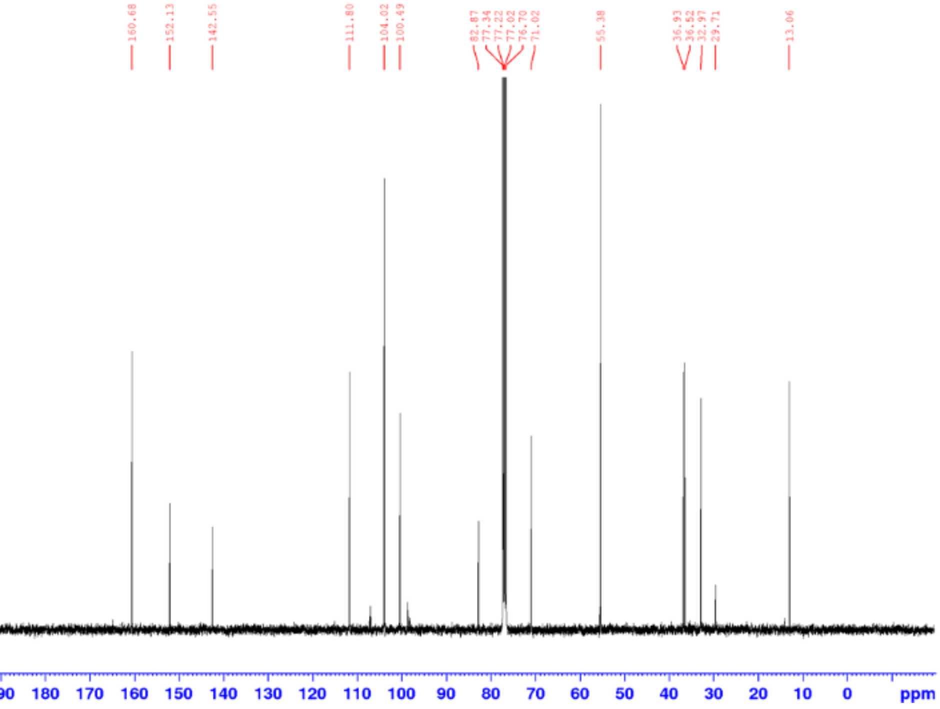

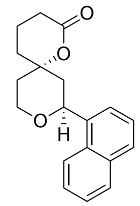

41, ^{1}H NMR
$CDCl_3$, 400 MHz

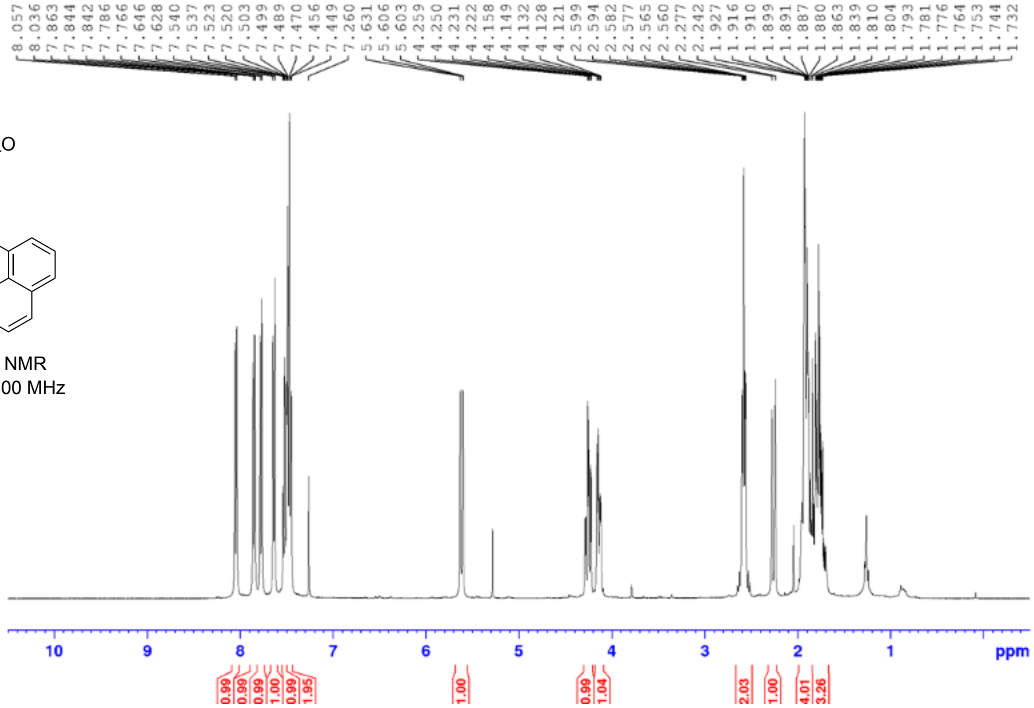

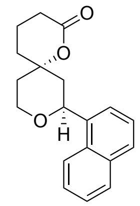

41, ^{13}C NMR
$CDCl_3$, 100 MHz

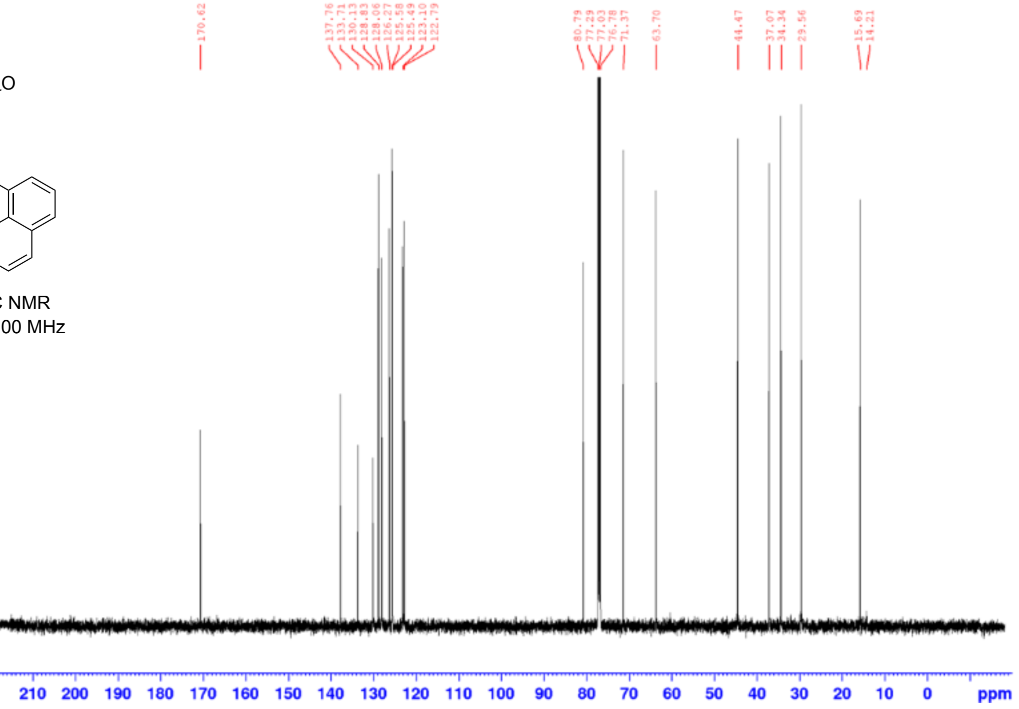

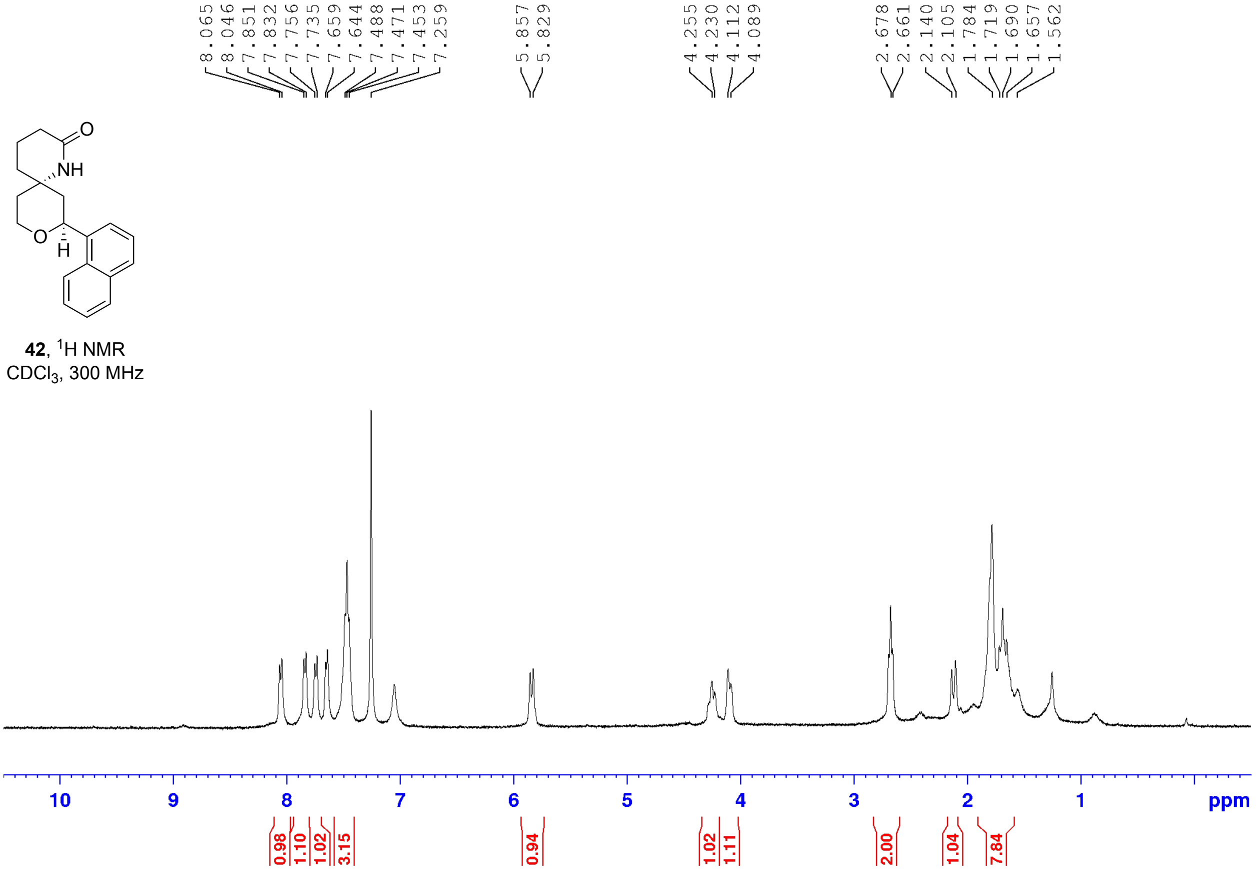

42, ^{1}H NMR
CDCl$_3$, 300 MHz
8.065 8.046 7.851 7.832 7.756 7.735 7.659 7.644 7.488 7.471 7.453 7.259
5.857 5.829
4.255 4.230 4.112 4.089
2.678 2.661 2.140 2.105 1.784 1.719 1.690 1.657 1.562
0.98 1.10 1.02 3.15 0.94 1.02 1.11 2.00 1.04 7.84
10 9 8 7 6 5 4 3 2 1 ppm

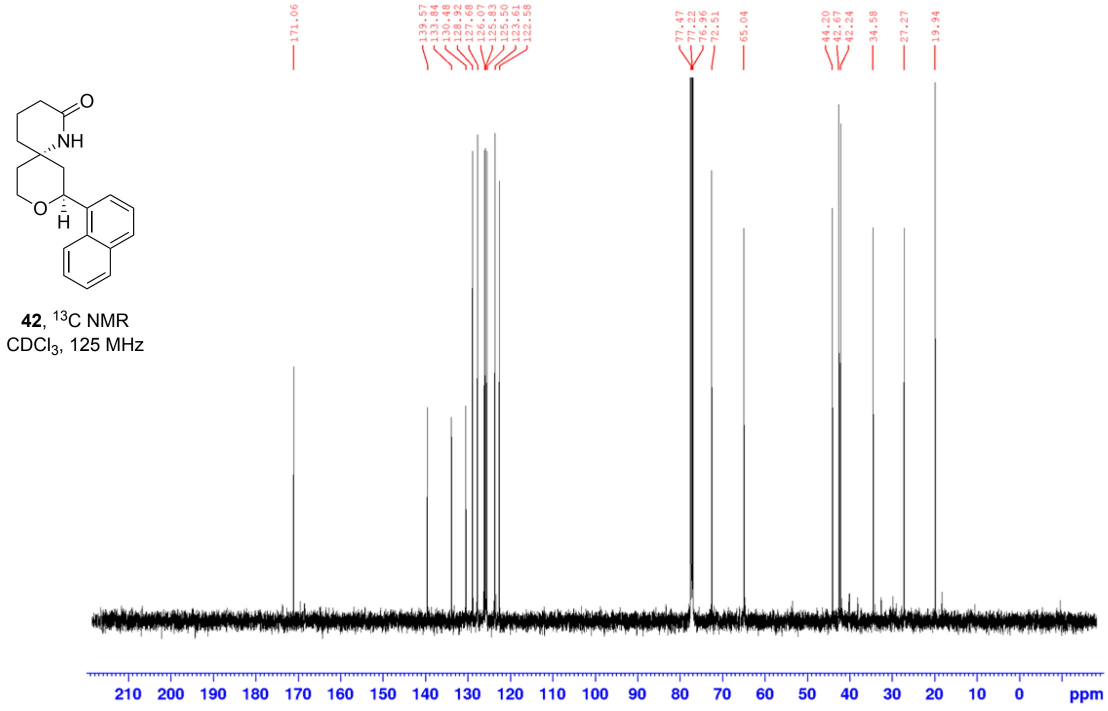

42, ^{13}C NMR
CDCl$_3$, 125 MHz
171.06
139.57 133.84 130.48 128.92 127.68 126.07 125.83 125.50 123.61 122.58
77.47 77.22 76.96 72.51 65.04
44.20 42.67 42.24 34.58 27.27 19.94
210 200 190 180 170 160 150 140 130 120 110 100 90 80 70 60 50 40 30 20 10 0 ppm

References

1) Skotnitzki, J.; Kremsmaier, A.; Keefer, D.; Gong, Y.; deVivie-Riedle, R.; Knochel, P. Stereoselective Csp^3–Csp^2 Cross-Couplings of Chiral Secondary Alkylzinc Reagents with Alkenyl and Aryl Halides. *Angew. Chem., Int. Ed.* **2020**, *59*, 320-324.

2) Race, N. J.; Bower, J. F. Palladium Catalyzed Cyclizations of Oxime Esters with 1,2-Disubstituted Alkenes: Synthesis of Dihydropyrroles. *Org. Lett.* **2013**, *15*, 4616-4619.

3) Hazelden, I. R.; Carmona, R. C.; Langer, T.; Pringle, P. G.; Bower, J. F. Pyrrolidines and Piperidines by Ligand-Enabled Aza-Heck Cyclizations and Cascades of *N*-(Pentafluorobenzoyloxy)carbamates. *Angew. Chem., Int. Ed.* **2018**, *57*, 5124-5128.

4) Tu, W.; Liu, L. Floreancig, P. E. Diastereoselective Tetrahydropyrone Synthesis through Transition-Metal-Free Oxidative Carbon–Hydrogen Bond Activation. *Angew. Chem., Int. Ed.* **2008**, *47*, 4184-4187.
