# Supplementary figures and images for: Development and Mechanistic Studies of Oxidative Prins-Semipinacol Cyclization Reactions
Source: Org Lett. 2026 Apr 13;28(16):5092–6. doi: 10.1021/acs.orglett.6c00814 (PMC13123410; doi:10.1021/acs.orglett.6c00814)

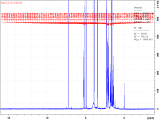

Supplement: Supplementary file 2 [file ol6c00814_si_002.zip › Prins FID Rev/10/HNMR/10/pdata/1/thumb.png]
